# Supplementary material for: Metronidazole and ether derivatives target Helicobacter pylori via simultaneous stress induction and inhibition
Source: Nat Microbiol. 2026 Mar 18;11(4):1049–63. doi: 10.1038/s41564-026-02291-w (PMC13056558; doi:10.1038/s41564-026-02291-w)
Supplement: Supplementary file 1 — Supplementary Figs. 1–26, Tables 1–6, supporting structural information, material and methods and uncropped gels of Supplementary Figs. 27–40. [file 41564_2026_2291_MOESM1_ESM.pdf]

nature microbiology

Supplementary information

https://doi.org/10.1038/s41564-026-02291-w

Metronidazole and ether derivatives target *Helicobacter pylori* via simultaneous stress induction and inhibition

In the format provided by the authors and unedited

Table of Content

Supporting Figures 2

Supplemental Tables 21

Supporting Structural Information 25

Material and Methods 27

1.1 General Remarks for Synthesis 27

1.2 Synthesis 28

1.3 Cloning 41

1.4 Gel-based Activity-based Protein Profiling (ABPP) in HeLa cells 45

1.5 SDS-Page 46

1.6 BCA Assay 46

1.7 Desalting and Filtration 46

1.8 MS Measurement and Data Analysis 47

1.9 Full Proteome Analysis 49

1.10 Binding Site Identification isoDTB Analysis 50

1.11 Intracellular EC_{50} Determination in *H. pylori* 53

1.12 Recombinant Protein Overexpression from *E. coli* 54

1.13 Purification of recombinantly expressed proteins 54

1.14 Intact Protein MS Measurement and Data Analysis 58

1.15 Recombinant Protein Labeling of HpTpx from *E. coli* 59

1.16 Analytical Protein Expression and *in situ* Labeling of HpTpx or Mutants in *E. coli* 59

1.17 Crystallography 60

1.18 Minimal Inhibitory Concentration Assay (MIC Assay) 62

1.19 DNA-based Antioxidant Activity Assay 62

1.20 MTT Assay 62

1.21 Plasma Stability Assay 63

1.22 AGS Adhesion Assay with *H. pylori* 26695 63

1.23 HPLC-MS/MS Analysis for ADME and PK Studies 64

1.24 *In vivo* Efficacy Studies in Murine Model 66

Uncropped Gels of Supplementary Figures 67

Supporting Figures

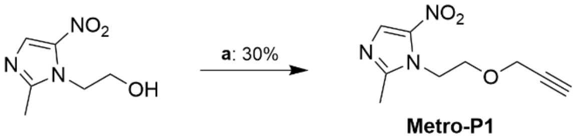

(**a**) 1.20 eq. propargyl bromide, 1.20 eq. Cs_2CO_3, DMF, 50°C, 48 h

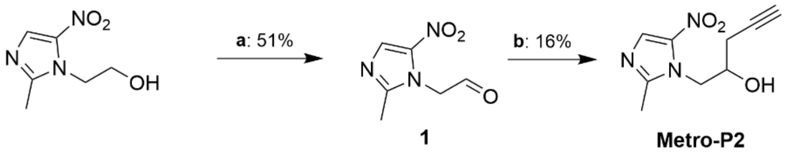

(**a**) $(COCl)_2$, DMSO, NEt_3, CH_2Cl_2, -78°C, 2h, (**b**) propargyl magnesium bromide, THF, r.t., 2h

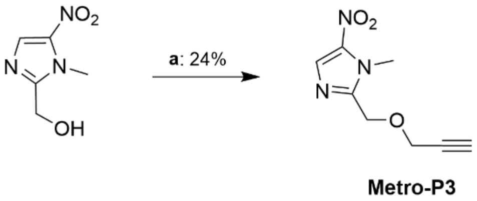

(**a**) 2.40 eq. propargyl bromide, 2.20 eq. Cs_2CO_3, DMF, 80°C, 15h

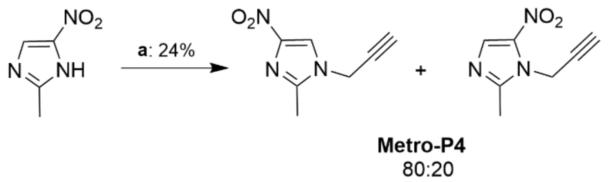

(**a**) 1.20 eq. propargyl bromide, 2.20 eq. K_2CO_3, DMF, 50°C, 26h

Figure S 1: Synthesis routes and reaction conditions for **Metro-P1, Metro-P2, Metro-P3** and **Metro-P4**.

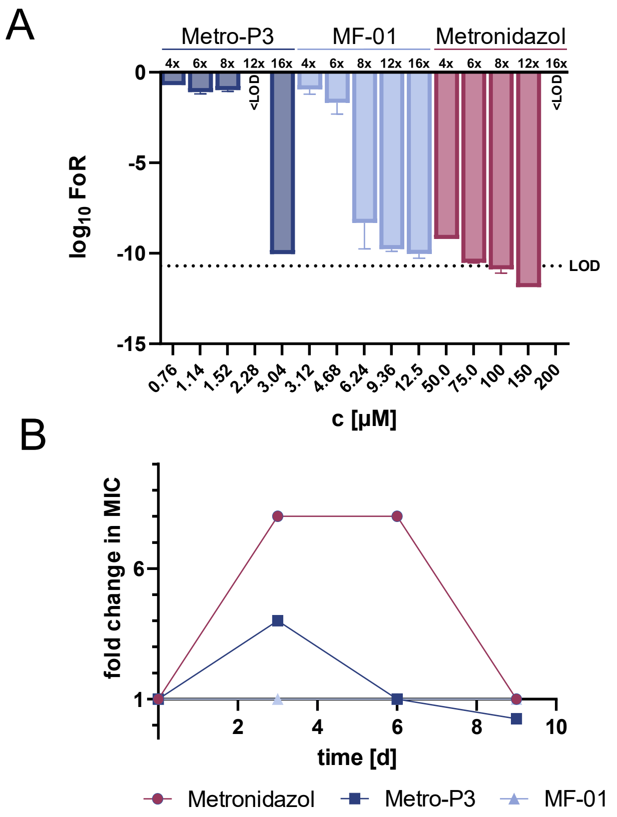

MIC [µM]	1x	4x	6x	8x
Metronidazol	12.5	50	75.0	100
Metro-P3	0.19	0.76	1.14	1.52
MF-01	0.78	3.12	4.68	6.24

Figure S 2: Resistance development assays of *H. pylori* 26695 in the presence of **Metronidazol**, **Metro-P3** and **MF-01** (**A**) Frequency of Resistance was determined by plating bacteria on agar containing 4×, 6×, 8×, 12× and 16× the MIC. Frequencies were calculated as the ratio of colonies on plates containing the compound to those on DMSO control plates. No resistant colonies were detected for **Metronidazol**$_{16x}$ and **Metro-P3**$_{12x}$ (LOD = 2 × 10^{-11}). Experiment was performed in two biological replicates (n_{bio} = 2). (**B**) Serial passaging in the presence of sub-MIC concentrations of antimicrobials. Bacterial growth was severely impaired after 6 days (*H. pylori* transform into a coccoid form under stress which is difficult to cultivate).

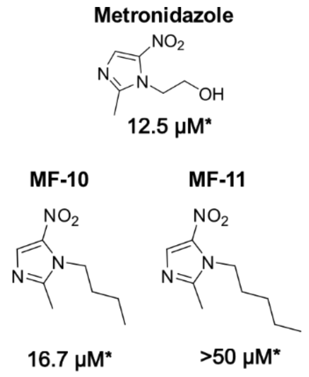

*MIC values in *H. pylori* 26695

Figure S 3: Overview of synthesized aliphatic nitroimidazole derivatives and respective MIC values in H. pylori 26695.

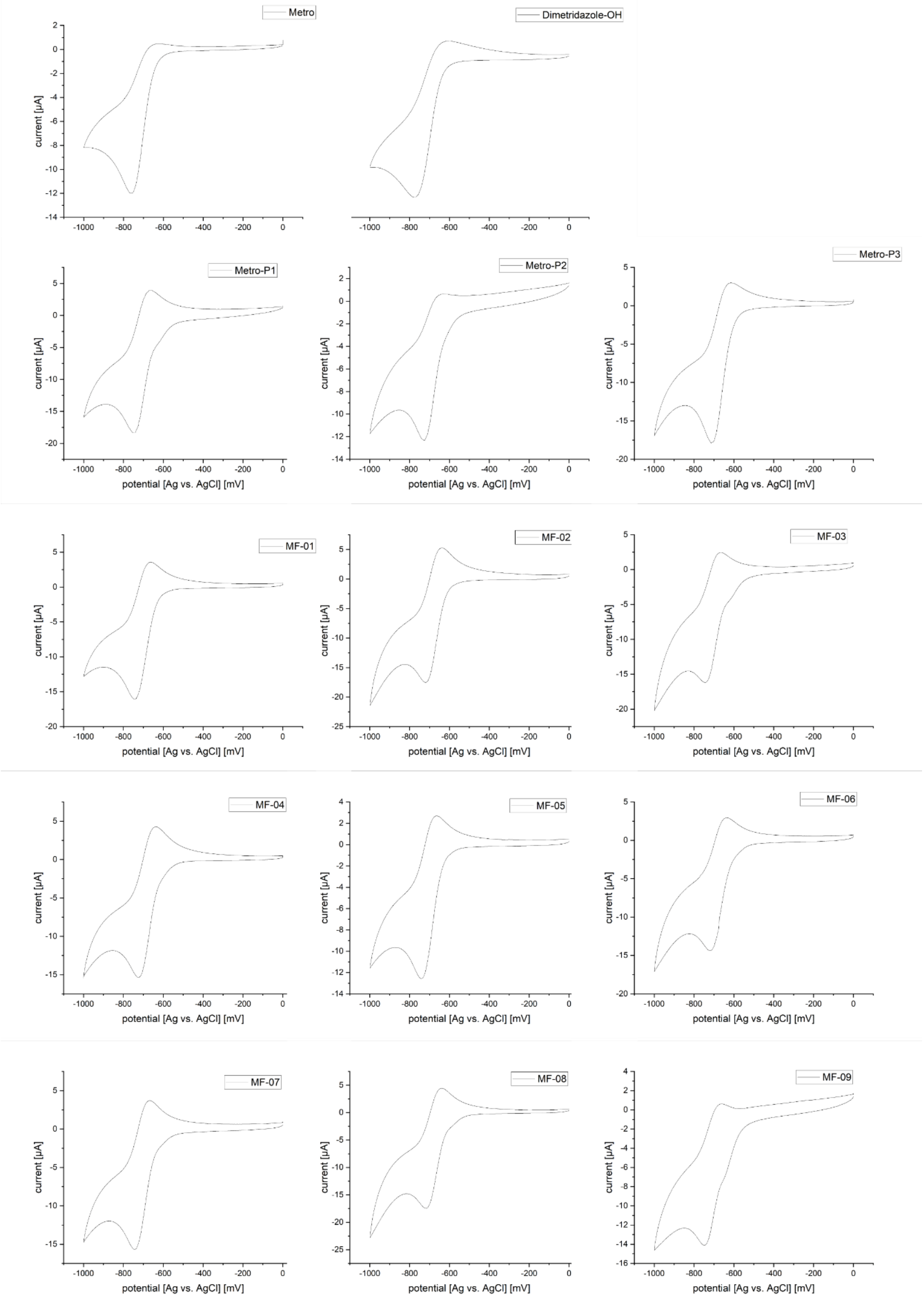

Figure S 4: Cyclic voltammetry (CV) measurements of 5-nitroimidazole compounds. Depicted is the measurement within the respective potential region encompassing the first reductive event from 0 mV to 1000 mV against potential [mV vs Ag/AgCl] for each compound. Generally, the first scan cycle is shown. Cyclic voltammograms were measured and are shown against E(Ag/AgCl). Redox potentials were reported referenced to the normal hydrogen electrode (NHE): E(Ag/AgCl;1 M KCl) = 236 mV vs NHE.

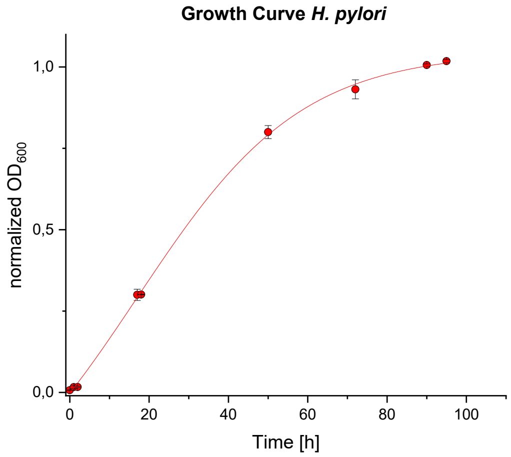

Figure S 5: Growth curve *H. pylori* 26695 under microaerophilic conditions at 37°C in a culture volume of 50 mL BHI + 10% FCS medium inoculated with 1:100 *H. pylori* overnight culture. OD_{600} was normalized to cultivation media (BHI + 10% FCS). Data represents mean values ±s.d of biological replicates (n = 3).

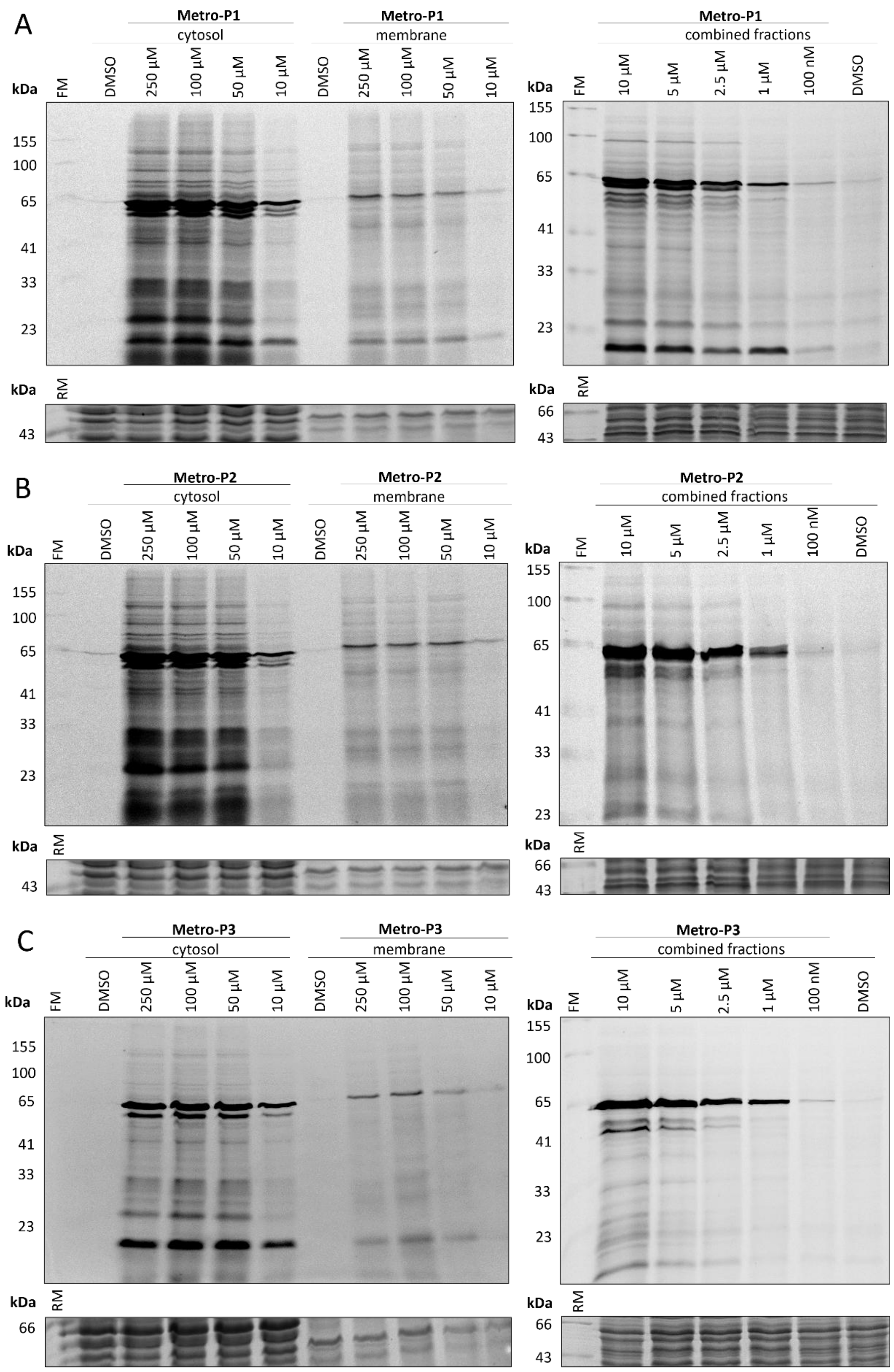

Figure S 6: Gel-based fluorescent ABPP labeling of **Metro-P1** (**A**), **Metro-P2** (**B**) and **Metro-P3** (**C**) in *H. pylori* 26695 (37 °C, 140 rpm, 2 h, microaerophilic conditions). Top: Fluorescence gel after *Click* reaction to rhodamine azide. Bottom: Coomassie-staining as loading control). FM = fluorescent marker, RM = Roti marker. *Click*-Chemistry was performed separately for cytosol and membrane fraction of cell lysate (left) or combined (right).

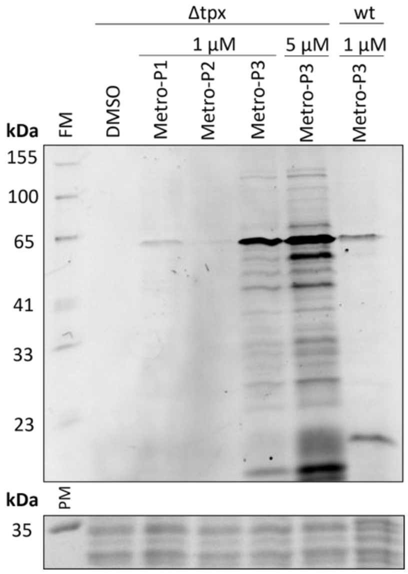

Figure S 7:Gel-based fluorescent ABPP labeling of **Metro-P1**, **Metro-P2** and **Metro-P3** in *H. pylori* 26695 Δ*tpx* and wt strain. Top: Fluorescence gel after Click reaction to rhodamine azide. Bottom: Coomassie-staining as loading control. FM = fluorescent marker, PM = PeqGold marker.

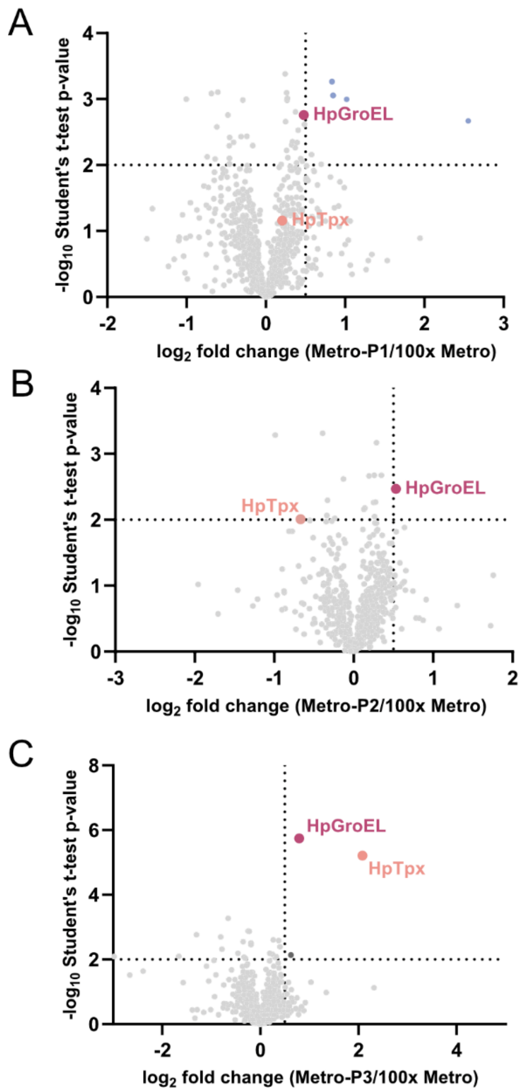

Figure S 8: Resulting volcano plots of MS-based competitive labeling experiments in *H. pylori* 26695 with **Metro-P1** (**A**), **Metro-P2** (**B**) and **Metro-P3** (**C**). Preincubation with metronidazole (100 µM, 100× excess) as competitor for 2 h (37 °C, 140 rpm, microaerophilic cond.) and subsequent incubation with probe (1 µM, 37 °C, 140 rpm, 2 h, microaerophilic cond.). Cut-off lines for p-values < 0.01 and $\log_2$ fold changes >0.5. HpGroEL and HpTpx shown as red and orange dots in all plots. Experiment was performed in biological triplicates (n = 3). Two-tailed Student's t-test was performed for statistical evaluation.

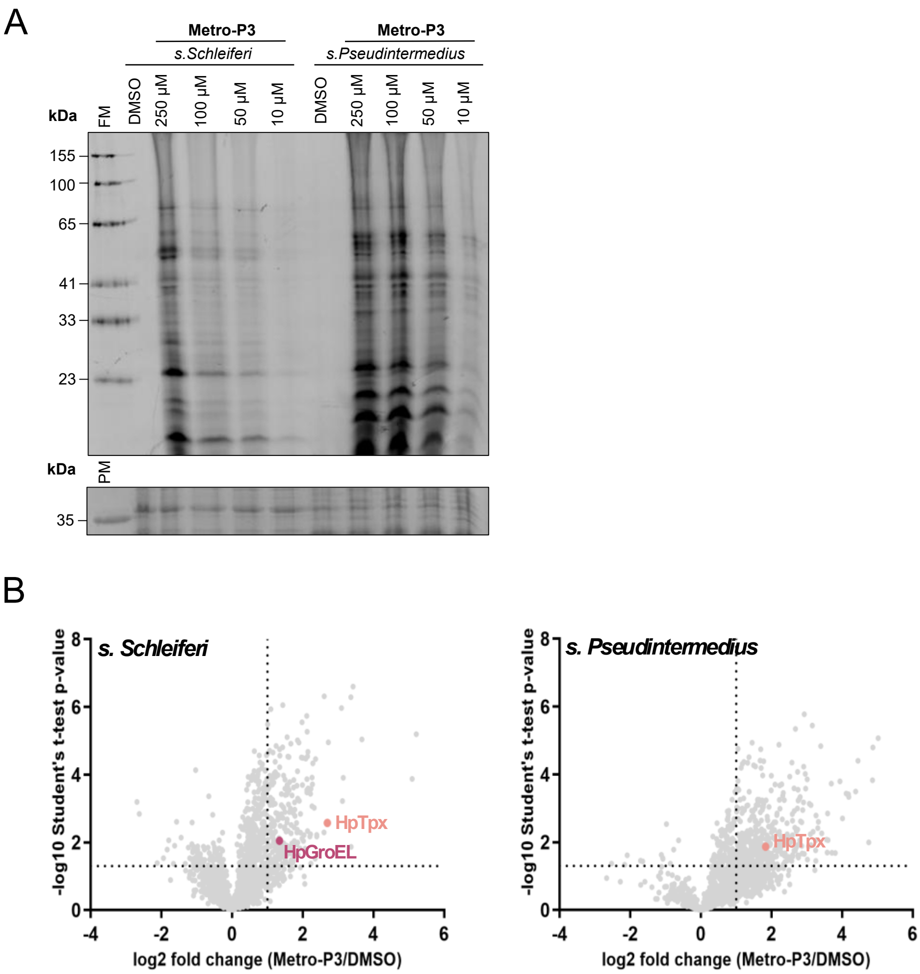

Figure S 9: Labeling experiments of **Metro-P3** in *S. schleiferi* and *S. pseudintermedius*. (**A**) Gel-based fluorescent ABPP labeling. Top: Fluorescence gel after Click reaction to rhodamine azide. Bottom: Coomassie-staining as loading control. FM = fluorescent marker, PM = PeqGold marker. (**B**) Resulting volcano plots of MS-based labelling. Incubation with probe (10 µM, 37 °C, 0 rpm, 2 h, anaerobic cond.). Cut-off lines for p-values < 0.01 and $\log_2$ fold changes >0.5. HpGroEL and HpTpx shown as red and orange dots in all plots. Experiment was performed in biological quadruplicates (n = 4). Two-tailed Student's t-test was performed for statistical evaluation.

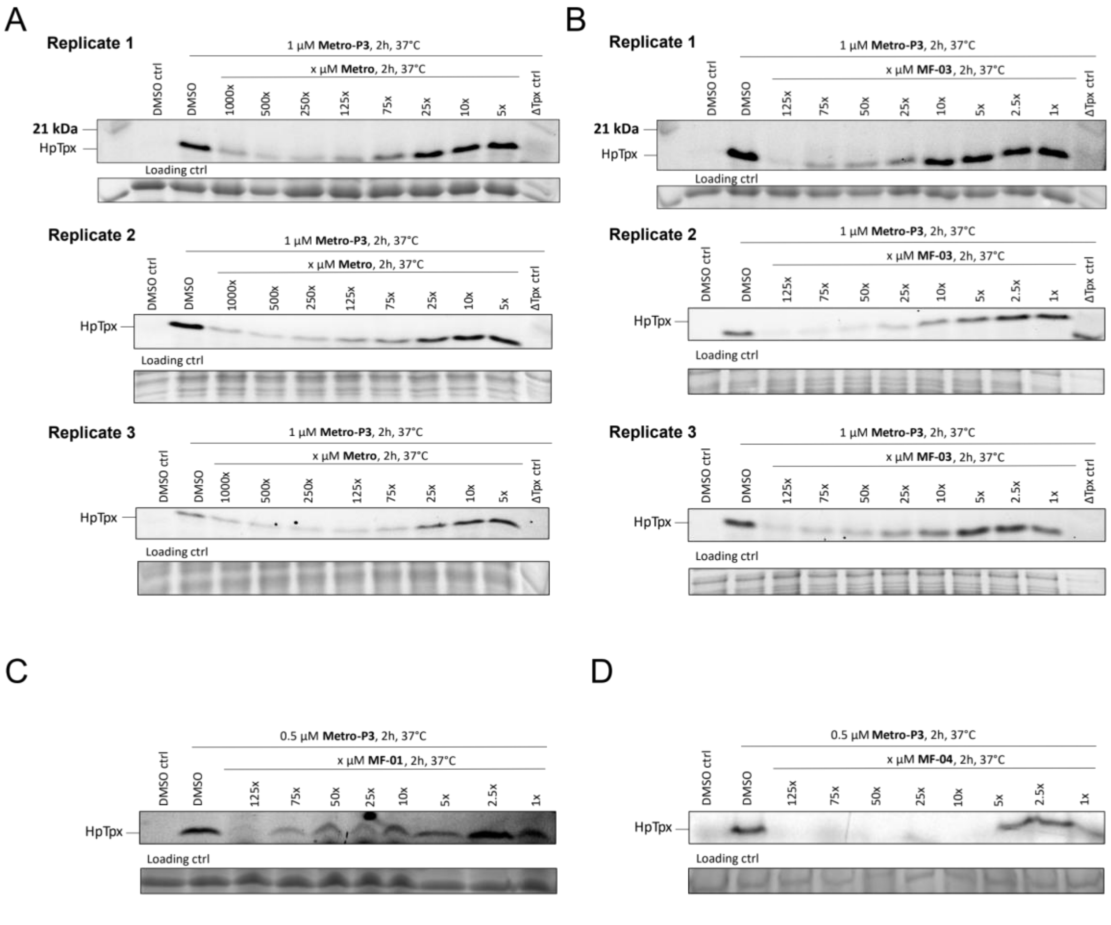

Figure S 10: Biological triplicates of gel-based competitive ABPP labeling studies in *H. pylori 26695 (*and Δ*tpx* as control) for apparent EC_{50} determination of HpTpx: Preincubation with different metronidazole (**A**), **MF-03** (**B**), **MF-01** (**C**) or **MF-04** (**D**) concentrations (37 °C, 140 rpm, 2 h, microaerophilic cond.) and subsequent incubation with probe (**Metro-P3**) (1 or 0.5 µM, 37 °C, 140 rpm, 2 h, microaerophilic cond.). Top: Fluorescence detection of SDS gel after *Click*-reaction with rhodamine azide. Bottom: Coomassie-staining of SDS gel (loading control).

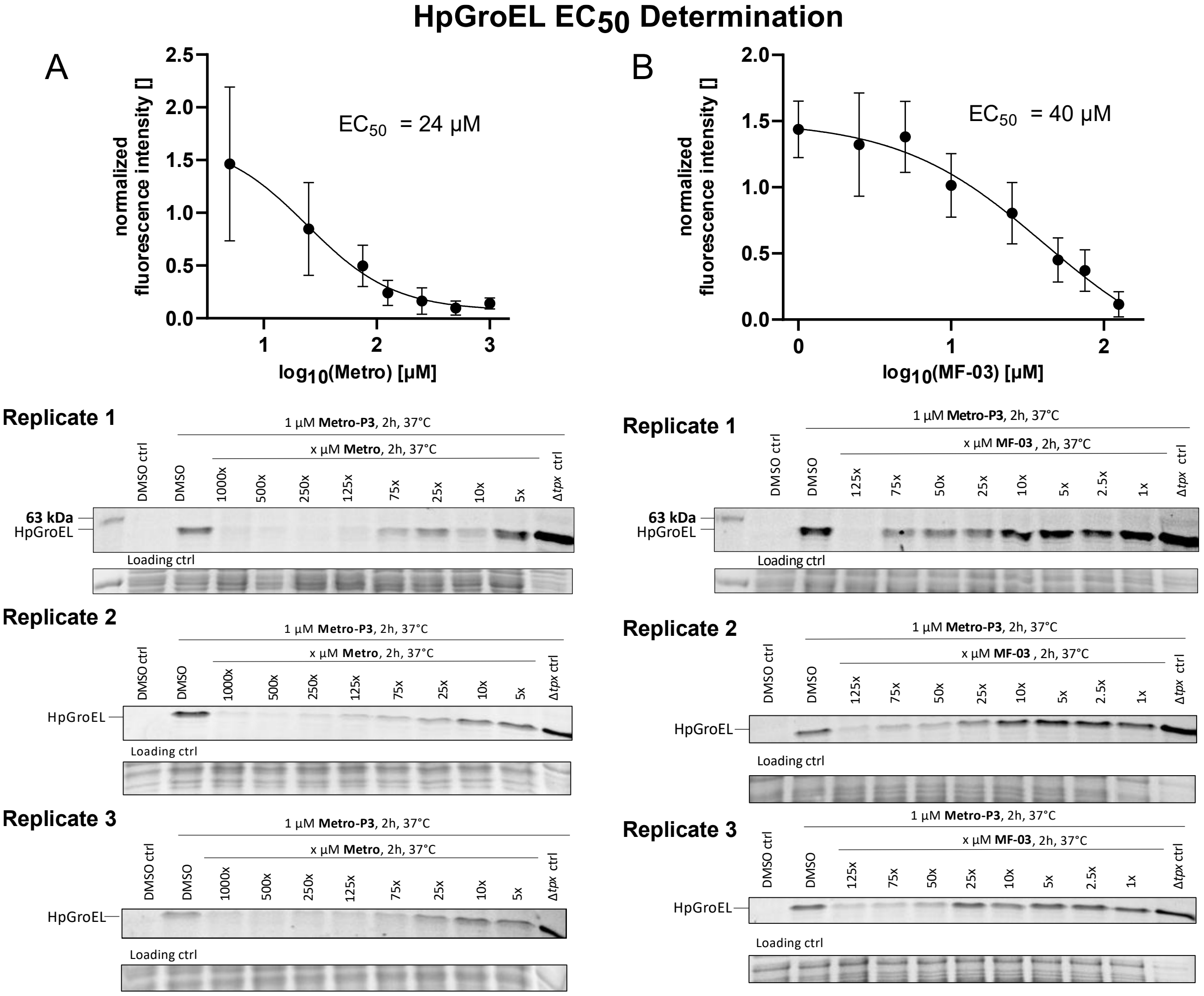

Figure S 11: Gel-based in situ competitive labeling in *H. pylori* 26695 for apparent EC_{50} value determination of HpGroEL. Pre-treatment of *H. pylori* 26695 with various Metronidazole (1× – 1000×, **A**) or **MF-03** (1× – 125×, **B**) concentrations for 2 h prior to the incubation with **Metro-P3** (1 µM, 2 h). Bacteria were harvested, lysed, clicked to rhodamine azide and fluorescent bands were visualized by SDS-Page. Top: fluorescence, bottom: Coomassie staining as loading control. Band intensities of HpGroEL were quantified with *ImageJ*[1]. Values were normalized to band intensities without addition of competitor and DMSO control was subtracted as baseline. Data shown represent mean values ± s.d. of 3 biological replicates (n = 3). Apparent relative EC_{50} values were determined using non-linear regression (log(inhibitor) vs. response - Variable slope (four parameters)).

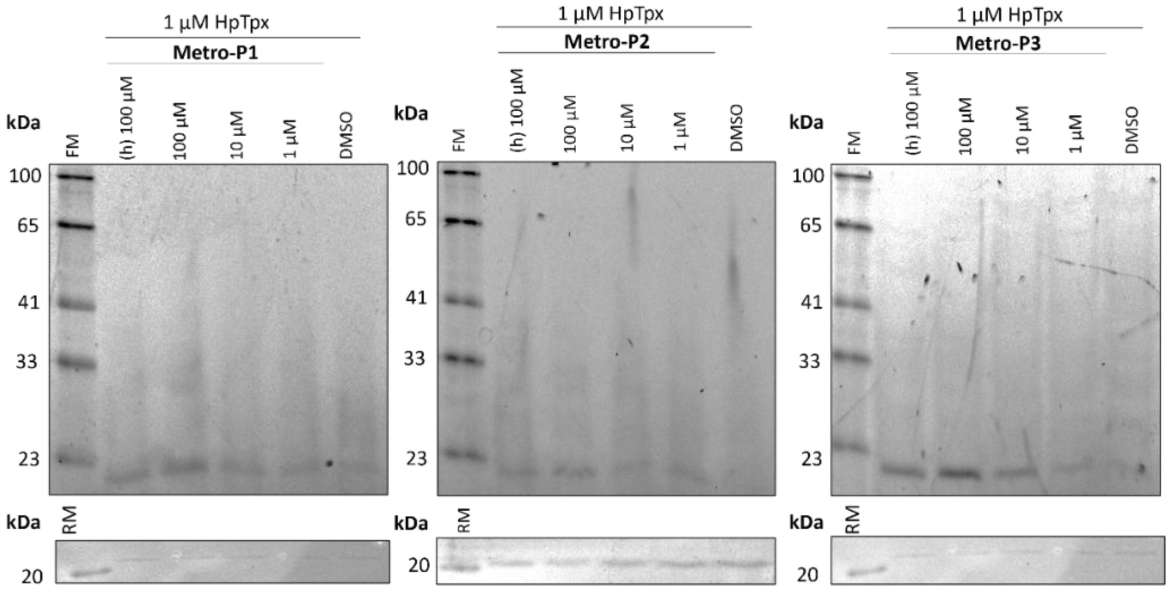

Figure S 12: Gel-based fluorescent labeling of recombinantly expressed and purified HpTpx **in vitro**. HpTpx (1 µM) was incubated with varying concentrations of **Metro-P1** (left), **Metro-P2** (center), **Metro-P3** (right) for 1 h at 37°C (200 rpm, aerobic) prior to Click reaction and SDS Page. Heat shock (h) was performed at 95°C for 20 min before incubation with probe. Slight background labeling observed for all conditions including heat shock and DMSO control. Top: fluorescence gel, bottom: Coomassie gel as loading control. FM= fluorescent marker, RM = Roti Marker.

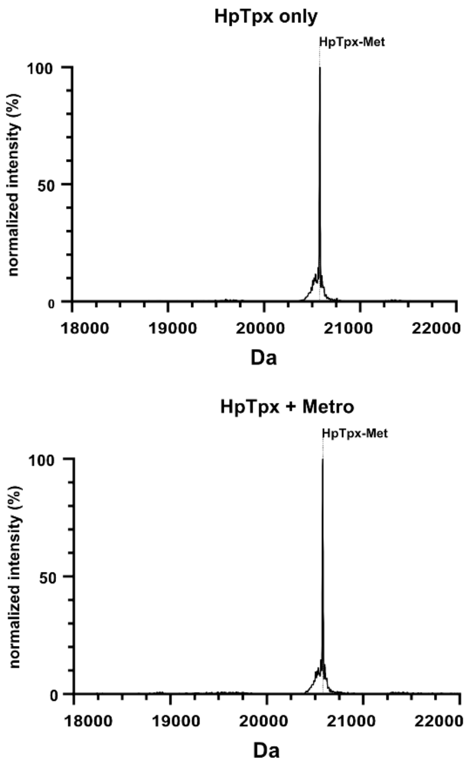

Figure S 13: Intact Protein MS (IP-MS) of recombinant labeling of HpTpx with Metronidazole (or **Metro-P1**, **Metro-P2**, **Metro-P3**) *in vitro*. Recombinantly expressed HpTpx (10 µM) was incubated with 50 µM compound for 1 h at 37°C before IP-MS analysis. Protein mass for HpTpx was found to be 20 580 Da corresponding to Strep-tagged HpTpx without start-methionine (HpTpx-Met). No mass shift was observed. Found mass with addition of compound: 20 580 Da (HpTpx-Met). Result is exemplarily shown for incubation with Metronidazole, identical results were observed for **Metro-P1**, **Metro-P2** and **Metro-P3** (data not shown).

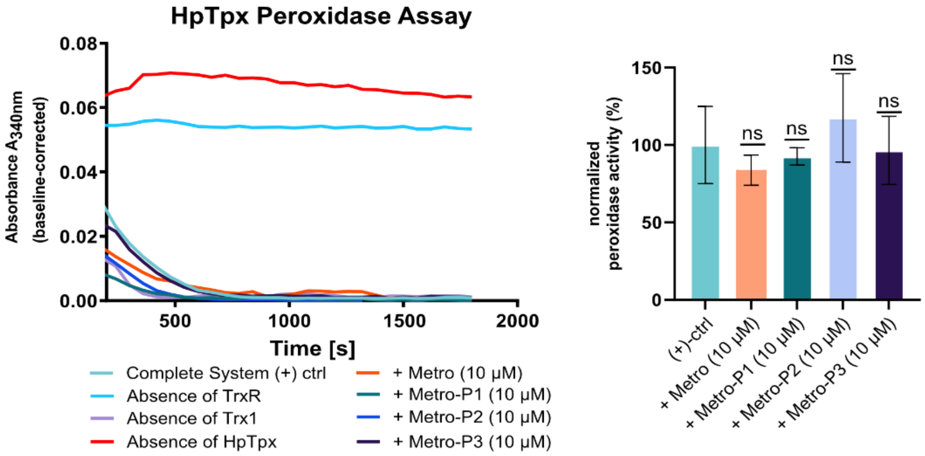

Figure S 14: HpTpx peroxidase assay with and without pre-incubation (30 min, r.t.) of HpTpx with Metronidazole (Metro), **Metro-P1**, **Metro-P2** and **Metro-P3** (10 µM). Absorbance at 340 nm was measured over time and baseline-corrected to reaction buffer (left). Normalized activity of HpTpx w or w/o addition of compound. was determined from initial slopes via simple linear regression of time-dependent absorbance measurements and normalized to the Complete system (+) ctrl (HpTpx activity 100%). Bar chart represents the mean ± SEM. of three technical replicates per condition (right). Two-tailed Student's t test was performed for statistical evaluation. n.s. = not significant, p-value > 0.05.

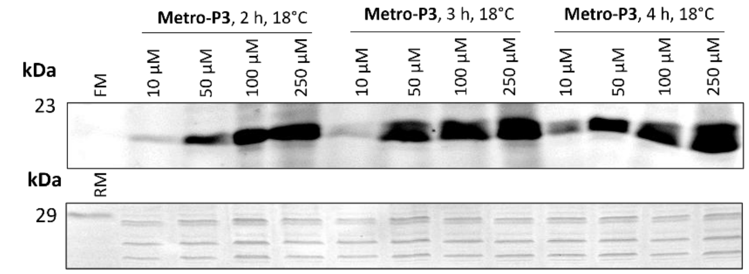

Figure S 15: Test gel expressions of HpTpx in *E. coli* to find suitable conditions to express enzymes with increasing degree of **Metro** or **Metro-P3** modification. After protein expression, bacteria were harvested, lysed and clicked to rhodamine azide prior to fluorescence SDS-Page. Top: fluorescence gel with band belonging to HpTpx. Bottom: coomassie gel as loading control. FM = fluorescent marker, RM = Roti marker.

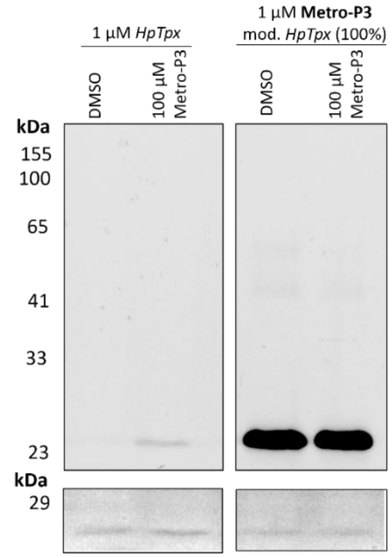

Figure S 16: Comparison of gel-based fluorescent labeling of recombinantly expressed and purified HpTpx (left) or with addition of **Metro-P3** during protein overexpression (1 mM, 18 h, o/n, right). HpTpx (1 µM) or modified HpTpx (100% **Metro-P3**) were incubated with **Metro-P3** (100 µM) or DMSO for 1 h at 37°C (200 rpm, aerobic) prior to Click chemistry and SDS Page. Top: fluorescence gel, bottom: Coomassie gel as loading control.

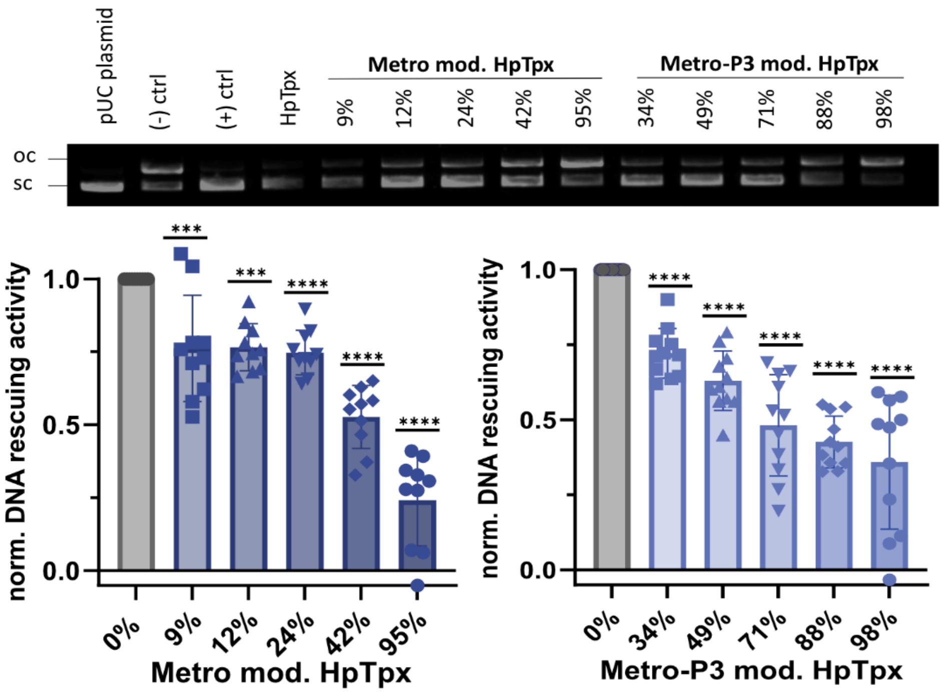

Figure S 17: DNA-based antioxidant activity assay of HpTpx. Top: Agarose gel of one biological replicate shown for evaluation of HpTpx ability to circumvent DNA damage. oc = open circular; sc = supercoiled form of DNA. Addition of EDTA during reaction served as positive control. Bottom: DNA rescuing activity was determined as the ratio of sc/oc form of DNA. Band intensities were quantified by *ImageJ*[1]. Obtained ratios were normalized to positive control ratio (100% activity) and negative control (0% activity) was subtracted as baseline. Graphs represent mean values ± s.d. of 10-12 biologically independent experiments (**Metro**: n = 10, **Metro-P3**: n = 12). Statistical significance was determined by ordinary one-way ANOVA with multiple comparisons (no correction) to control column (0%). p-values: >0.05 (ns), < 0.05 (*), < 0.01 (**), < 0.001 (***), < 0.0001 (****).

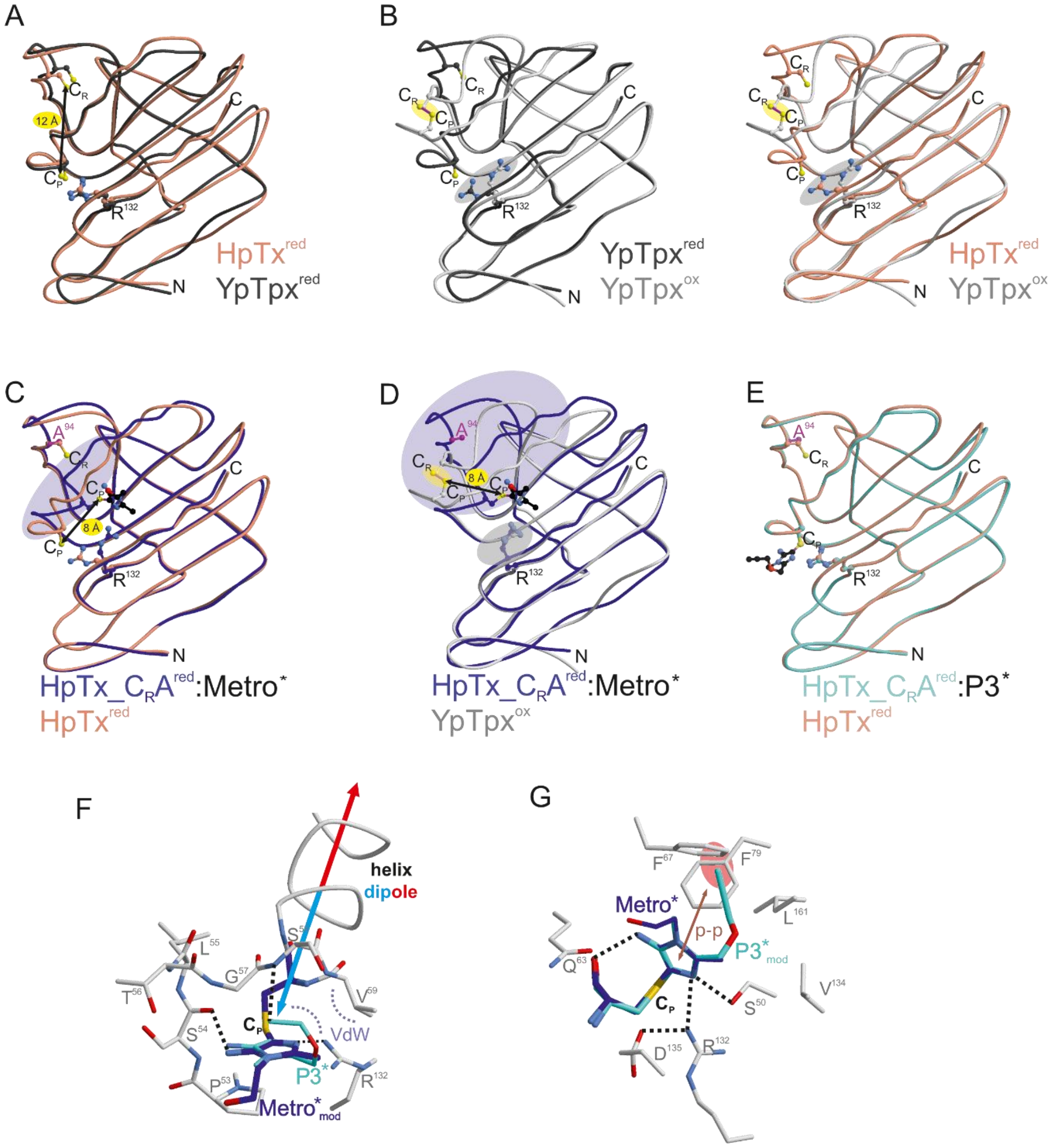

Figure S 18: **Comparison of *H. pylori* thiol peroxidase structures**. (**A**) Superposition of $HpTpx^{red}$ (orange, PDB ID 9F5V) and $YpTpx^{red}$ (dark grey, PDB ID 2XPD)[2] reveals high structural similarities. The active site cysteines are positioned 12 Å apart. The strong coordination of C_P to Arg132 favors a thiolate ion, thereby enhancing its redox-active nucleophilicity. (**B**) *Left panel:* The comparison between $YpTpx^{red}$ (grey) and $YpTpx^{ox}$ (white, PDB ID 3ZRD)[2] illustrates significant structural rearrangements within the active site upon disulfide formation (pink) between C_P and C_R. Notably, the side chain of Arg132 is flipped by 90° in the oxidized variant. *Right panel:* Superposition of $YpTpx^{ox}$ with $HpTpx^{red}$. (**C**) The overlay of HpTpx_C_RA:**Metro*** (purple, PDB ID 9F64) with $HpTpx^{red}$ elucidates major structural rearrangements at helix α1 (light blue) around the ll lines

C_P region, while the mutated $C_R{}^A$ site maintains the same conformation as observed in the apo structure (rmsd 0.9 Å, 94% C^{α}-atoms). Notably, the distance between C_P and C_RA (pink) is decreased to 8 Å. (**D**) The comparison between HpTpx_C_RA:Metro* and $YpTpx^{ox}$ demonstrates major structural differences around C_P and C_R. Interestingly, the Arg132 side chain adopts a similar conformation in the two structures, but no longer interacts with the sulfur atom of C_P. (**E**) The HpTpx_C_RA:**Metro-P3*** complex (**P3***, cyan, PDB ID 9F65) adopts the same conformation as apo $HpTpx^{red}$ (rmsd 0.4 Å, 99% C^{α}-atoms,) and presents the inhibitor surface exposed at the C_P site. (**F**) **Metro*** (carbon atoms in purple) is modelled into the HpTpx_C_RA:**Metro-P3*** complex according to **Figure 4D**, bottom. While **Metro**$^*_{mod}$ nicely fits into the substrate binding pocket, interactions with protein side chains are less pronounced compared to **Metro-P3***. In addition, the absence of the propargyloxy group prevents **Metro*** from being stabilized by the dipole moment of helix α1 (Ser58NH). (**G**) In HpTpx_C_RA, modelled **Metro-P3*** (P3$^*_{mod}$, carbon atoms in cyan) is superimposed on **Metro*** (see **Figure 4D**, top). However, the non-physiological specificity pocket in HpTpx is too small for the propargyloxy residue and severe clashes with Phe67 (highlighted in red) prevent this arrangement. * amine form of respective compounds upon reduction.

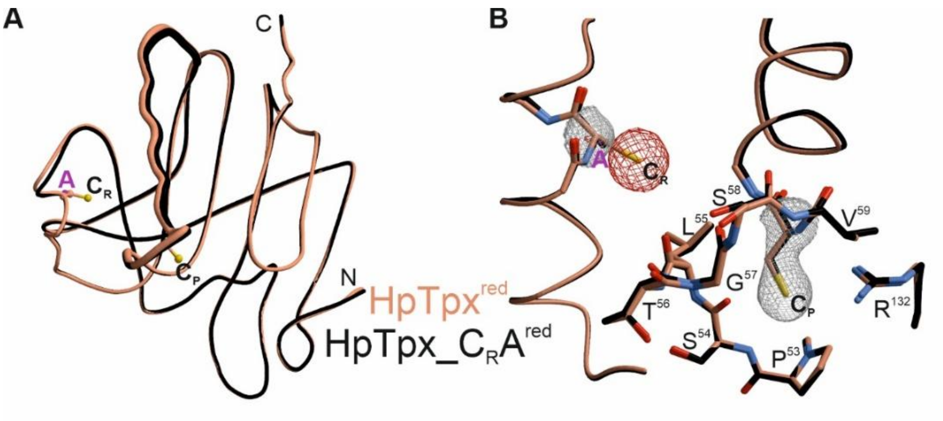

Figure S 19: Structural comparison of *H. pylori* thiol peroxidase (HpTx) wild type and C94A variant. (**A**) Superposition of HpTpx^{red} (orange, PDB ID: 9F5V) and HpTpx_C_RA^{red} (black, PDB ID: 9F65; Ala94 in magenta) reveals high structural similarity, with an RMSD of 0.3 Å over 99% of C_α atoms. (**B**) A close-up view of the active sites shows that all residues adopt identical positions. The $2F_o$-F_c electron density map (grey mesh, contoured to 1.0σ) is displayed for C_p and Ala94 in HpTpx_C_RA^{red}. The F_o-F_c map (red mesh, contoured at −3.0σ) clearly indicates the absence of the sulfur atom when Cys94 is modelled in the HpTpx_C_RA^{red} structure.

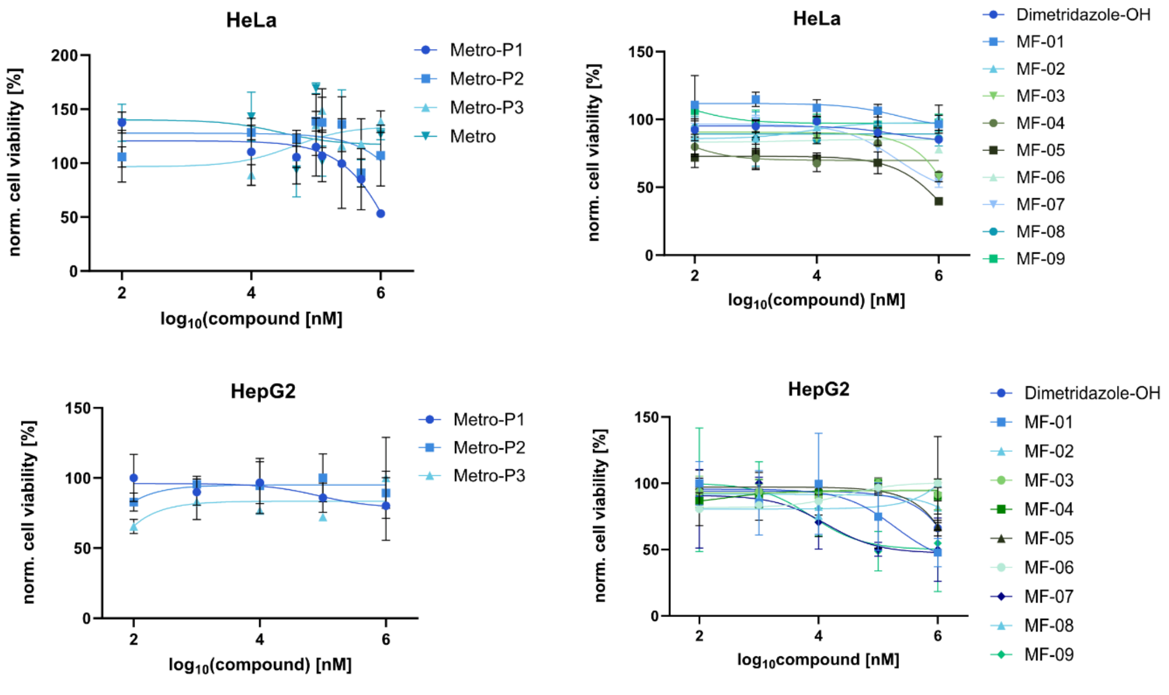

Figure S 20: IC_{50} toxicity evaluations of 5-nitroimidazole compounds in HeLa cells (top) and HepG2 cells (bottom). Values were normalized to DMSO treated cells. Data shown represent mean values ± s.d of technical triplicates (n = 3). Curves were fitted using non-linear regression (log(inhibitor) vs. response - Variable slope (four parameters)) revealing no IC_{50} values for all compounds.

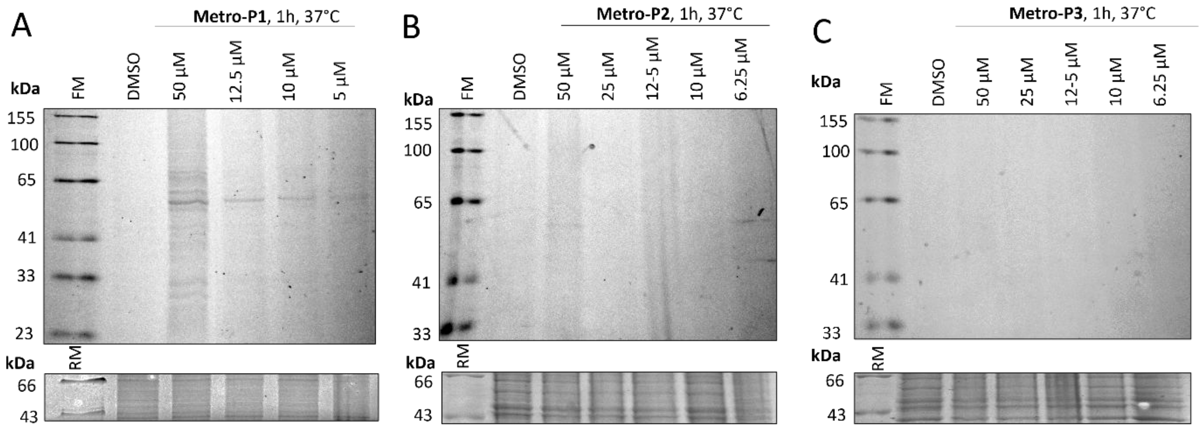

Figure S 21: Gel-based ABPP labeling experiments with varying concentrations of **Metro-P1** (**A**), **Metro-P2** (**B**) and **Metro-P3** (**C**) in HeLa cells. Incubation was performed for 1 h at 37 °C (5% CO_2). Top: Fluorescence detection of SDS gel after Click reaction with rhodamine azide. Bottom: Coomassie-staining of SDS gel (loading control). FM = fluorescent marker, RM = Roti marker.

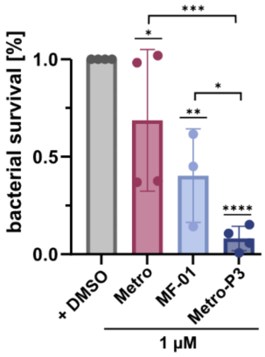

Figure S 22: Eradication of H. pylori 26695 in a gastric adenocarcinoma (AGS) adhesion assay with **Metro**, **MF-01** and **Metro-P3** (1 µM). For normalized values, CFUs were normalized to DMSO growth control. Bar charts represent mean values ± s.d. of 3 (**MF-01**) or 4 (**Metro-P3, Metro, DMSO**) biologically independent experiments with three technical replicates per experiment (n_{bio} = 3). Statistical significance was determined on normalized data with ordinary one-way ANOVA with multiple comparisons (no correction). p-values: >0.05 (ns), < 0.05 (*), < 0.01 (**), < 0.001 (***), < 0.0001 (****).

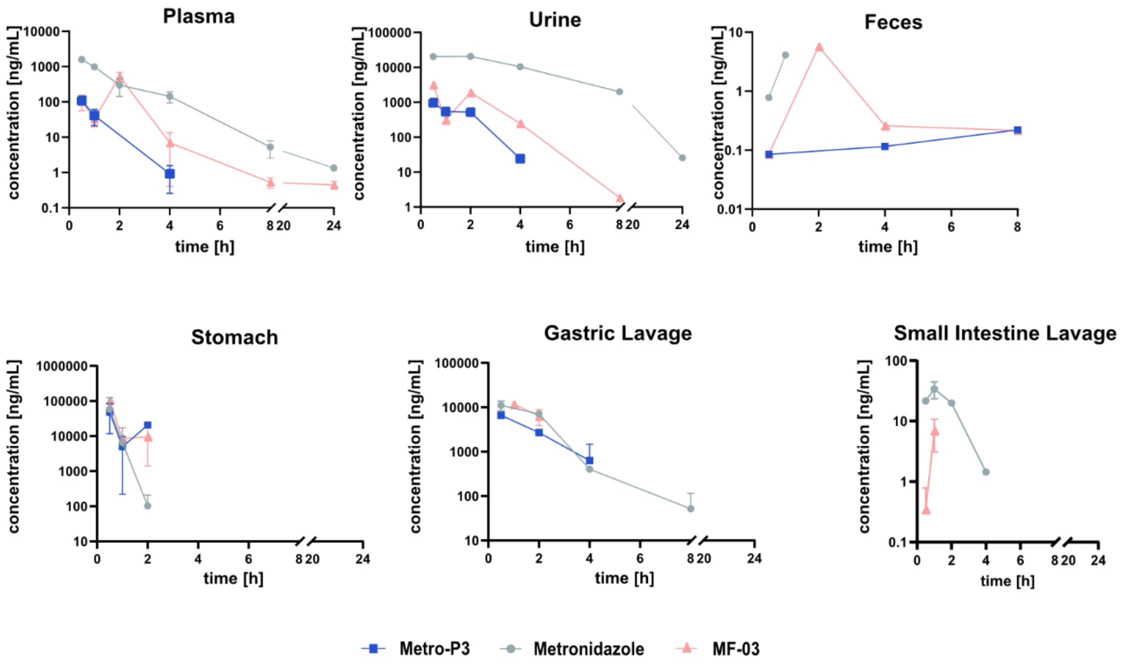

Figure S 23: Focused Pharmacokinetic (PK) data of **Metronidazole** (MIC = 2140 ng/mL), **Metro-P3** (MIC = 40 ng/mL) and **MF-03** (MIC = 80 ng/mL) in plasma, urine, feces and stomach (dose: 7.2 mg/kg PO, n = 3 mice per compound). Graph represents mean ± s.d. of 3 replicates (n_{bio} = 3), where applicable. Gastric and small intestine lavages were performed with isotonic NaCl.

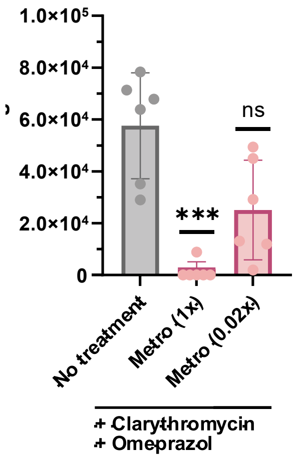

Figure S 24: In vivo efficacy studies in C57BL/6 mice infected with *H. pylori* SS1 strain and treatment with different regimens: No treatment (n = 6), standard triple therapy consisting of clarithromycin (7.15 mg/kg/day) and omeprazol (400 µmol/kg/day) with Metro (14.2 mg/kg/day, n = 6) or low dose of Metro (0.02 × Metro = 0.30 mg/kg/day ; n = 6). Bar charts represent mean values ± s.d. Statistical significance was determined by nonparametric Kruskal-Wallis test with multiple comparisons (no correction) to control column (no treatment). p-values: > 0.05 (ns), < 0.001 (***). The limit of detection (LOD) for this assay was calculated as 12.7 CFU/mg, corresponding to one colony on the lowest dilution plated.

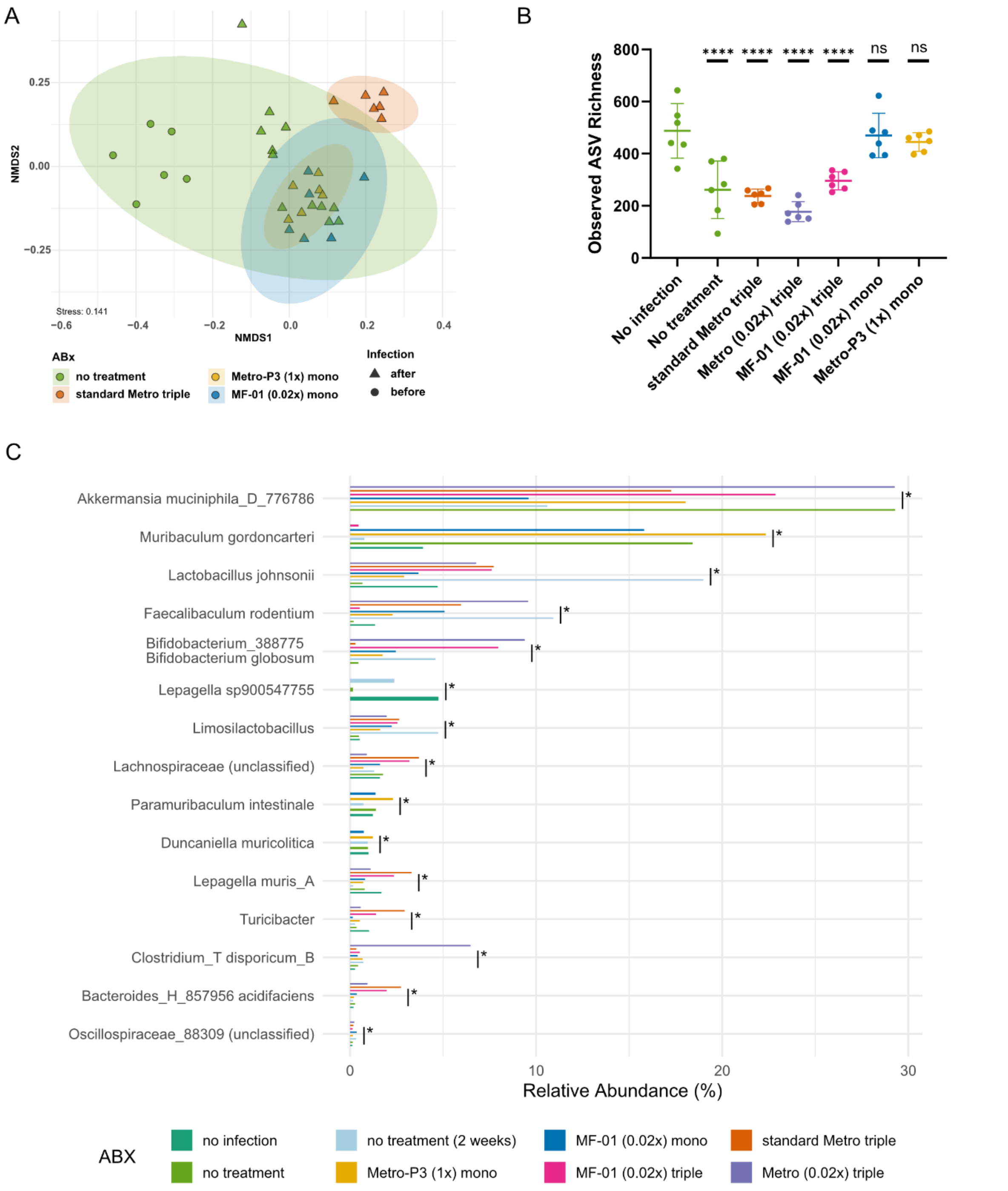

Figure S 25: Fecal microbiome studies of C57BL/6 mice infected with *H. pylori* SS1 strain and treatment with different regimens: No infection (n = 6), No treatment (n = 6), standard triple therapy consisting of clarithromycin (7.15 mg/kg/day) and omeprazol (400 μmol/kg/day) with **Metro** (14.2 mg/kg/day, n = 6), low dose of **Metro** (0.02 × Metro = 0.30 mg/kg/day; n = 6), low dose of **MF-01** (0.02 × Metro = 0.30 mg/kg/day; n = 6), mono therapy with low dose of **MF-01** (0.02 × Metro = 0.30 mg/kg/day; n = 6), mono therapy with **Metro-P3** (14.2 mg/kg/day; n = 6). (**A**) NMDS ordination of fecal microbiome profiles based on Bray–Curtis dissimilarity. Each point represents a fecal sample, colored by treatment group. Distances reflect community similarity; ellipses indicate 95% confidence intervals for each group. Pairwise PERMANOVA p−values: no treatment vs standard Metro triple: 0.001; no treatment vs MF-01 (0.02x) mono: 0.013; no treatment vs Metro-P3 (1x) mono: 0.006; standard Metro triple vs MF-01 (0.02x) mono: 0.002; standard Metro triple vs Metro-P3 (1x) mono: 0.002; MF-01 (0.02x) mono vs Metro-P3 (1x) mono: 0.002 (**B**) observed ASV richness. Statistical significance was determined by nonparametric Kruskal-Wallis test with multiple comparisons (no correction) to control column (no treatment). p-values: > 0.05 (ns), < 0.0001 (****). (**C**) Differentially abundant species. p-values: < 0.05 (*).

Supplemental Tables

Table S 1: MIC values of **Metro** and **Metro-P3** in (moderately) resistant clinical isolates of *H. pylori* 26695 (n = 3). MIC values were determined in three biological replicates (n = 3). For MIC values given as a range, the higher MIC value was observed in one biological replicate.

H. pylori **Clinical isolate**	**MIC [µM]**	
	**Metro**	**Metro-P3**
18	31.3-62.5	62.5-125
23	125	125
25	15.6	0.48
27	31.3-62.5	1.95
31	62.5	125
32	31.25	0.48
42	125	7.8
46	62.5	15
52	31.3-62.5	3.9-7.8
57	31.3-62.5	0.48
126	125	62.5
129	125	62.5-125
143	15.6-31.25	0.48
162	62.5-125	15.6-31.3

Table S 2: Measured MIC values of **Metronidazole**, **Metro-P1**, **Metro-P2**, **Metro-P3** and **Metro-P4** in different bacterial strains: *H. pylori* (microaerophilic), *C. difficile* (anaerobic), *E. coli* (aerobic), *S. aureus* (aerobic) in three biological replicates (n = 3).

MIC values	***H. pylori***	***C. difficile***	***E. coli***	***S. aureus***
**Metronidazole**	12.5 µM	1 µM	>1 mM	>1 mM
**Metro-P1**	390 nM	6.25 µM	>1 mM	>1 mM
**Metro-P2**	3.13 µM	1 µM	>1 mM	>1 mM
**Metro-P3**	195 nM	6.25 µM	>1 mM	>1 mM
**Metro-P4**	50 µM	n.d.	>1 mM	>1 mM
Dimetridazole		500 nM		
MF-01		6.25 µM		
MF-02		6.25 µM		

Table S 3: Summarized mean values of *in vitro* data of synthesized 5-nitroimidazole compounds. MIC values were determined in three biological replicates (n = 3). For MIC values given as a range, the higher MIC value was observed in one biological replicate. Reduction potential E_{red} was measured against normal hydrogen electrode (NHE). MTT assays were performed in technical replicates (n = 3) in two different cell lines. Plasma stability assays were performed in 3 biological replicates (n=3).

**Compound**	**MIC (*H. pylori* 26695)**		**MIC *(H.pylori* PMSS1)**	**E_{red} (NHE) [mV]**	**MTT IC_{50} (HeLa)**	**MTT IC_{50} (HepG2)**	**plasma stability [t=28h]**
**Dimetridazole-OH**	3.13 µM	0.49 µg/mL	n.d.	-536	n.d.	n.d.	n.d.
**Metro**	12.5 µM	2.14 µg/mL	3.125 µM	-530	> 1 mM	n.d.	94%
**Metro-P1**	390 nM	80 ng/mL	< 97 nM	-516	> 1 mM	> 1 mM	62%
**Metro-P2**	3.13 µM	0.65 µg/mL	780 nM - 1.56 µM	-489	> 1 mM	> 1 mM	84%
**Metro-P3**	195 nM	40 ng/mL	195 nM - 390 nM	-479	> 1 mM	> 1 mM	43%
**MF-01**	780 nM	140 ng/mL	< 97 nM	-506	> 1 mM	> 1 mM	38%
**MF-02**	390 nM	70 ng/mL	195 nM - 390 nM	-484	> 1 mM	> 1 mM	0%, ($t_{1/2}$ = 3.8 h)
**MF-03**	390 nM	80 ng/mL	< 97 nM	-508	> 1 mM	> 1 mM	81%
**MF-04**	390 nM	70 ng/mL	390 nM	-489	> 1 mM	> 1 mM	0% ($t_{1/2}$ < 2 h)
**MF-05**	390 nM	80 ng/mL	< 97 nM	-506	> 1 mM	> 1 mM	>99%
**MF-06**	390 nM	80 ng/mL	390 nM	-482	> 1 mM	> 1 mM	>99%
**MF-07**	390 nM	80 ng/mL	< 97 nM	-508	> 1 mM	> 1 mM	87%
**MF-08**	390 nM	80 ng/mL	390 nM	-482	> 1 mM	> 1 mM	33%
**MF-09**	1.56 - 3.13 µM	75 ng/mL	390 nM	-511	> 1 mM	> 1 mM	81%

Table S 4: ADME profiles of six derivatives **Metro-P1**, **Metro-P3**, **MF-01**, **MF-02**, **MF-03** and **MF-07**. PPB = plasma protein binding. Blue = compounds with sufficient threshold values.

	Mouse half-life micro-somes [min]	Mouse clearance [µl/min /mg protein]	Human half-life micro-somes [min]	human clearance [µl/min/mg protein]	mouse plasma half-life [min]	Human plasma half-life [min]	PPB Mouse [%]	PPB Human [%]
**Metro-P1**	17.3	80.0	11.2	124.0	> 240	> 240	66.2	68.9
**Metro-P3**	> 60	< 23	> 60	< 23	> 240	> 240	45.1	50.0
**MF-01**	> 60	< 23	> 60	< 23	> 240	> 240	76.7	74.4
**MF-02**	> 60	< 23	> 60	< 23	> 240	> 240	62.6	61.8
**MF-03**	> 60	< 23	> 60	< 23	> 240	> 240	75.5	69.4
**MF-07**	43.3	32.0	57.8	24.0	> 240	> 240	75.3	72.5

Table S 5: *H. pylori* MS-based ABPP proteins hits for **Metro-P1** (1 µM), **Metro-P2** (1 µM) and **Metro-P3** (1 µM) selected based on an enrichment ratio of $\log_2$ >1.5 and p-value of <0.01 and additionally filtered for q-values of <0.05. Fold Enr. = Fold Enrichment, $\log_2$(difference); Sign. = Significance, $-\log_{10}$(p-value). Two-tailed Student's t test (probe vs DMSO control) was performed for statistical evaluation. Each condition was performed in biological triplicates (n = 3).

Probe	UniProt	Protein Name (Gene Name)	Fold Enr.	Sign.
**Metro-P1**	O25096	NH_3-dependent NAD^+ synthetase (*nadE*)	4.00	4.29
	O25151	Thiol peroxidase (*tpx*)	1.73	3.19
	O25874	Aminopyrimidine aminohydrolase	1.23	2.73
	P42383	60 kDa chaperonin	1.08	3.62
**Metro-P2**	O25096	NH_3-dependent NAD^+ synthetase (*nadE*)	3.33	3.98
	O25356	7-cyano-7-deazaguanine synthase	1.04	2.62
	P42383	60 kDa chaperonin	1.04	3.46
**Metro-P3**	O25151	Thiol peroxidase (*tpx*)	4.27	5.49
	P42383	60 kDa chaperonin (*groEL*)	1.17	4.21

Table S 6: Measured MIC values of **Metronidazole**, **Metro-P3** and **MF-01** in different anaerobic bacterial strains of the murine microbiome: *Bacteroides caecimuris* (I48), *Muribaculum intestinale* (YL27) (anaerobic), *Enterococcus faecalis* (KB1), *Bifidobacterium animalis* (YL2) in two biological replicates (n = 2).

	**Gram-negative**		**Gram-positive**	
**MIC [µM]**	*B. caecimuris* I48	*M.intestinale* YL27	*E. faecalis* KB1	*B. animalis* YL2
**Metro**	6.25 - 12.5	0.78	>50	>50
**Metro-P3**	25	3.13	>50	>50
**MF-01**	25	1.56	>50	>50

Table S 7: Plasmids used within this work.

Plasmid	Description	Source
**pDONR201**	Gateway Donor vector, attP1, attP2, KanR, pUC ori, CmR, ccdB	*Invitrogen*
**pET-55-dest-HpTpx**<br>**pET-55-dest-HpTpx-C60A**<br>**pET-55-dest-HpTpx-C94A**<br>**pET-55-dest-HpTpx-C60A-C94A**	attB1, attB2, AmpR, lacI, pUC ori, T7 promotor, Strep-tag II sequence (Nterminal), HpTpx cloned into attR1 and attR2 sites of pET-55-DEST, expression clone	This study
**pET-28a(+)**	KanR, lacI, pUC ori, T7 promoter, MCS	*Twist Bioscience*
**pET-28a(+)-HpGroEL**	KanR, lacI, pUC ori, T7 promoter, HpGroEL with TEV cleavage site an C-terminal His tag cloned into pET28a(+) using NcoI and XhoI restriction sites	This study
**pOND708**	derivative of pBlu-SK-alt with homologous regions to *hydA* and *mdaB* flanking *rpsL-cat*	Debowski et al[3]
**pTpx_rpsL-cat**	derivative of pOND708 with homologous regions to the upstream and downstream DNA sequence of *tpx* gene flanking *rpsL-cat*	This study

Supporting Structural Information

SSI1

To elucidate the molecular mechanism of Metro and Metro-P3 inhibition, we solved high-resolution structures of HpTpx alone and with both ligands. The protein was heterologously expressed in *Escherichia coli* and purified by StrepTag affinity and size exclusion chromatography. While the oxidized conformation of HpTpx did not crystallize, we first determined the structure of the reduced state (HpTpx^{red}) at 1.75 Å resolution (PDB ID 9F5V, **Table S17**). HpTpx^{red} forms a homodimer with two identical catalytic centers and, similar to the thiol peroxidase (Tpx) from *E. coli*[4], each subunit is composed of a five-stranded β-sheet flanked by four α-helices. A structural homology search using the DALI server[5] identified Tpx from *Yersinia pseudotuberculosis* (YpTpx) as the best hit[2] (PDB ID: 2XPD, Z-score = 24.3, backbone root mean square deviation (rmsd) = 1.2 Å, 93% C$^{\alpha}$-atoms, sequence identity 39%). In HpTpx^{red}, classified as an atypical 2-Cys Tpr, the peroxide-binding Cys60 (C_P) is located at the N-terminal part of helix α1 (**Figure S17A**). The strong coordination of C_P to Arg132 reduces the pK_a value at the sulfur atom by stabilizing the thiolate ion and increasing its redox-active nucleophilicity. During catalysis, C_P reacts covalently with hydroperoxides to form a cysteine-sulfenic acid adduct (C_P-SOH). The subsequent condensation of C_P-SOH occurs via the resolving Cys94 residue (C_R) in helix α2, resulting in HpTpx^{ox} with an intramolecular disulfide bridge between C_P and C_R (**Figure S17B**).

Next, the structure of HpTpx in complex with Metro was determined. However, inhibition requires a stepwise reduction of the nitro group in Metro prior to nucleophilic aromatic substitution by HpTpx. To this end, we have heterologously expressed a C94A mutant (HpTpx_C_RA) in the presence of **Metro** and demonstrate that only the reduced ligand (**Metro***) is bound to the variant. The crystal structure of HpTpx_C_RA in complex with **Metro*** displays the inhibitor fully defined in the F_o-F_c electron density map at 1.75 Å resolution (PDB ID 9F64, **Table S16**), which forms an irreversible thioether bond with the C_P residue (**Figure 4D**, top). While the superposition of HpTpx_C_RA:Metro* with HpTpx^{red} exhibits identical conformations in the mutated Ala94 region (rmsd 0.9 Å, 94% C$^{\alpha}$-atoms, **Figure S17C**), significant structural rearrangements occur in the ligand bound structure at the catalytic center of the C_P residue. Helix α1 is shortened by two turns, distorted, and C_P is shifted by 8 Å compared to HpTpx^{red}. Intriguingly, **Metro*** generates a well-defined specificity pocket at the active site that is absent in HpTpx^{red} as well as in YpTpx^{ox} (**Figure S17D**).[2] The interaction between the protein and **Metro*** is mediated by rearrangements of the aromatic amino acids Phe67, Phe79, and Tyr158 that stabilize the ligand's imidazole scaffold by π-π stacking (**Figure 4D,** top). Notably, Arg132 remains close to C_P and forms strong hydrogen bonds with Asp135-O$^{\varepsilon 1}$ as well as Ser50-O$^{\gamma}$. Furthermore, Arg132 and Ser50 are H-bonded to the sp^2-hybridized N3 nitrogen atom of **Metro***. The hydroxyethyl side chain at N1 interacts with Gln63$^{\varepsilon 1}$, while the methyl group at C2 engages in van-der-Waals contacts with Val134, Pro158, and Leu161. A prominent hydrogen bond, formed between the generated amine group of **Metro*** at C4 and the carbonyl oxygen of C_P, provides a rationale for the inhibition of HpTpx^{red} by the ligand. This coordination is impossible with a nitro group and explains why Metro only acts as an HpTpx inhibitor upon its reduction.

While **Metro** and **Metro-P3** share high structural similarities, the latter compound is a 60-fold more potent antibiotic. We therefore solved the crystal structure of HpTpx_C_RA with **Metro-P3*** (nitro group reduced to amine) at 1.95 Å resolution (PDB ID 9F65, **Table S16**). As expected, the ligand forms a covalent thioether bond with C_P (**Figure 4D**, bottom), but surprisingly, HpTpx

retains apo conformation (rmsd 0.4 Å, 99% C^{α}-atoms, **Figure S17E**). Subtle shifts allow for hydrogen bonds between the side chain of Arg132 and the main chain atom Ile152O as well as the sp^2-hybridized N3 nitrogen of **Metro-P3***. The methylene moiety within the propargyloxy side chain of **Metro-P3*** interacts via van-der-Waals contacts with the aliphatic residues Val59 and Leu153. The nucleophilicity of the propargyl group may be enhanced via the helix dipole (α1) moment of Ser58NH (distance 3.2 Å) and aligns the entire inhibitor within the spacious substrate binding pocket. Most importantly, however, the robust hydrogen bonds formed between **Metro-P3***-NH_2 and the carbonyl oxygen of Ser54O explain why the prodrug **Metro-P3** with its nitro group cannot act as an inhibitor of HpTpx.

Although **Metro*** and **Metro-P3*** form the same irreversible thioether bond with C_P in the native state of $HpTpx^{red}$, the different ligand coordination in HpTpx_C_RA:**Metro*** versus HpTpx_C_RA:**Metro-P3*** is highly unexpected (**Figure 4E**). While the HpTpx_C_RA:**Metro*** complex depicts substantial structural rearrangements in helix α1, such conformational changes are absent in HpTpx_C_RA:**Metro-P3***. The modeling of **Metro*** in the HpTpx_C_RA:**Metro-P3*** structure demonstrates a precise fit within the substrate binding pocket (**Figure S15F**). However, the less pronounced interactions of **Metro*** with enzyme residues and the absence of the propargyloxy group, crucial for a prominent helix-dipole interaction in HpTpx_C_RA:**Metro-P3***, are consistent with its phenotypic 60-fold enhanced biological activity. Conversely, the rigid and bulky propargyloxy group of **Metro-P3*** fails to be accommodated into the binding pocket of **Metro*** due to severe clashes with Phe67 (**Figure S17G**).

Intriguingly, the diverse binding modes of **Metro-P3*** and **Metro*** provide valuable molecular insights into HpTpx catalysis. In the former complex, the C_P and C_R residues maintain the same positions as in the $HpTpx^{red}$ structure (**Figure S17E**), even though C_P forms a thioether bond with **Metro-P3***. Notably, the propargyloxy group of the inhibitor stabilizes this state via the dipole moment of helix α1, allowing Arg132 to preserve its orientation despite the disruption of its ionic interaction with C_P (**Figure 4D**, bottom). In contrast, this stabilization is absent in HpTpx_C_RA:**Metro***, resulting in increased flexibility within the C_P region (**Figure 4D, top**). Here, Arg132 adopts the conformation previously observed in $YpTpx^{ox}$ (**Figure S17D**).[2] Although **Metro*** induces a structural distortion of helix α1, the entropic penalty is balanced by enthalpic stabilization of the inhibitor within an induced non-physiological specificity pocket. Thus, C_P modification by either the natural substrates, **Metro*** or **Metro-P3*** disrupts the salt bridge between the C_P thiolate and Arg132 residues and induces flexibility in the surface-exposed helix α1. However, structural changes in the C_R region may only occur in the presence of the natural C_P-SOH reaction intermediate that initiates the condensation via C_P-C_R disulfide bond formation.

Material and Methods

1.1 General Remarks for Synthesis

Reagents and Solvents

Unless otherwise stated, commercially available reagents and starting materials are obtained from *Sigma Aldrich*, *TCI Europe*, *VWR*, *Roth*, BLDpharm and *Alfa Aesar* and starting compounds were used without further purification and stored as indicated. All reactions sensitive to air and moisture were carried out using standard *Schlenk* techniques under argon atmosphere in flame-dried glassware. Anhydrous solvents and water-sensitive liquid chemicals are transferred using argon flushed syringes.

Thin Layer Chromatography (TLC)

For thin layer chromatography (TLC) silica coated plates (aluminium, *Merck*, silica 60 F254) were used. For visualization, the spots were detected by using UV-light (254 nm and 366 nm) or by staining with a potassium permanganate solution (3.00 g $KMNO_4$, 20.0 g K_2CO_3 and 5.00 mL 5 % NaOH (aq.) in 300 mL water) followed by heat treatment. Column chromatography was carried out using silica gel (40-63 μm (Si 60)) from *Merck*.

High Pressure Liquid Chromatography (HPLC)

Compounds were purified using preparative, reversed-phase HPLC using a *Waters* 2545 quaternary gradient module equipped with a fraction collector on an *YMC* Triart C18 column (250 × 10 mm, 5 μm). Gradient is listed in **Table S7** using ddH_2O and HPLC-grade acetonitrile (no TFA) as the mobile phase.

Table S 8: Used gradient for HPLC purification.

**t (min)**	**H_2O (%)**	MECN **(%)**
0	98	2
1	98	2
12	2	98
13	2	98
14	2	98
15	98	2
17	98	2

Nuclear Magnetic Resonance Spectroscopy (NMR)

Nuclear Magnetic Resonance (NMR) spectra were measured at room temperature either on a *Bruker* AVHD-400 or AVHD-300. The chemical shifts are given in δ values in ppm (parts per million) and calibrated to the residual proton signals of the solvents relative to the internal standard tetramethylsilane:

Chloroform-d_1 (^{1}H-NMR: δ = 7.26 ppm, ^{13}C-NMR: δ = 77.2 ppm)

The NMR multiplicities are given as singlets (s), doublets (d), triplets (t), quartets (q) , pseudo-sextets (ps) or multiplets (m). The coupling constants *J* are reported in Hertz (Hz). NMR data were analysed using MestReNova (Mestrelab Research)

Mass Spectrometry (MS)

High-Resolution Mass Spectrometry (HR-MS) was performed on a LTQ-FT Ultra mass spectrometer (*Thermo Fisher Scientific*). ESI is used as an ionization method. Low resolution LC-MS measurements were conducted on a MSQ Plus mass spectrometer (*Thermo Fisher Scientific*). Processing of mass spectrometry data was done by Xcalibur 2.2 (*Thermo Fisher Scientific*).

1.2 Synthesis

(**1**) 2-methyl-5-nitro-1-(2-(prop-2-yn-1-yloxy)ethyl)-*1H*-imidazole - **Metro-P1**

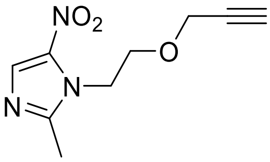

Metro-P1

To a suspension of metronidazole (200 mg, 1.17 mmol, 1.00 eq.) and Cs_2CO_3 (838 mg, 2.57 mmol, 2.20 eq.) in DMF (4 mL) is added propargyl bromide solution (80 % in toluene, *Sigma Aldrich*: P51001, 0.30 mL, 2.78 mmol, 2.40 eq.) and heated to 50 °C for 96 h. The reaction mixture is quenched *via* addition of H_2O (5 mL) and extracted with EtOAc (3 × 20 mL). Combined organic layers are subsequently washed with 5 % LiCl solution (aq., 15 mL) and brine (20 mL), dried over Na_2SO_4 and the solvent is removed *in vacuo*. The crude product is further purified performing column chromatography (EtOAc 100 %) and high-performance liquid chromatography (HPLC) to obtain 74.4 mg of the desired probe **Metro-P1** (1.17 mmol, 30 %) as a white solid.

TLC: R_f = 0.45 (EtOAc) [UV].

^{1}H NMR (400 MHz, $CDCl_3$) δ (ppm) = 2.39 (t, ^4J = 2.3 Hz, 1H), 2.52 (s, 3H), 3.86 (t, ^3J = 4.9 Hz, 2H), 4.08 (d, ^4J = 2.3 Hz, 2H), 4.51 (t, ^3J = 4.9 Hz, 2H), 7.96 (s, 1H).

^{13}C NMR (101 MHz, $CDCl_3$) δ (ppm) = 14.8, 46.6, 58.7, 68.6, 75.3, 78.8, 133.4, 152.0.

HR-MS (ESI): *m/z* = calc. $[M+H]^+$: 210.0878, found: 210.0874.

(**2**) 2-(2-Methyl-5-nitro-*1H*-imidazol-1-yl)acetaldehyde

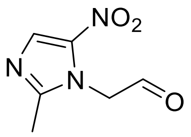

Synthesis of (**2**) is adapted from a published procedure.[6]

To 160 mL of CH_2Cl_2 is added dropwise 2.00 mL (20.0 mmol, 1.10 eq.) of oxalyl chloride under Argon atmosphere. The solution is cooled to −78 °C and 10 mL of DMSO is added dropwise to the stirred solution. After 20 minutes, 3.42 g (20 mmol, 1.00 eq.) of metronidazole dissolved in 15 mL of DMSO is added. After 20 min of additional stirring, 33 mL (240 mmol, 12.0 eq.) of NEt_3 is added. The reaction mixture is stirred for 10 min at −78 °C and is then allowed to warm to room temperature. The mixture is diluted with EtOAc (400 mL) and washed with water (4 × 75 mL). The water phase is extracted with EtOAc (3 × 50 mL). The combined organic layers are washed with brine (150 mL), dried over Na_2SO_4 and the solvent is removed *in vacuo*. The resulting crude residue is purified by flash silica gel chromatography (CH_2Cl_2/MeOH 40:1 v/v) to give the target compound (**1**) (1.20 g, 7.09 mmol, 32 %) as an orange, viscous oil.

TLC: R_f = 0.78 (CH_2Cl_2/MeOH 10:1) [UV].

^{1}H NMR (400 MHz, $CDCl_3$) δ (ppm) = 2.41 (s, 3H), 5.21 (s, 2H), 7.99 (s, 1H), 9.75 (s, 1H).

^{13}C NMR (101 MHz, $CDCl_3$) δ (ppm) = 14.0, 54.8, 132.8, 132.9, 150.4, 191.6.

LR-MS: *m/z* = calc. $[M+H]^+$: 170.06, found: 170.11.

(**3**) (1-(2-Methyl-5-nitro-*1H*-imidazol-1-yl)pent-4-yn-2-ol) - **Metro-P2**

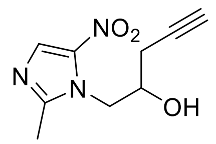

Metro-P2

The *Grignard* preparation of propargylmagnesium bromide was adapted from a literature procedure by *Ratsch et al.* [7]

A mixture of 620 mg (25.5 mmol, 1.78 eq.) of magnesium turnings, 3.56 g (15.8 mmol, 1.10 eq.) of $ZnBr_2$ and iodine (5 mol %) in dry THF (5 mL) is stirred for 15 min at room temperature. Then, a solution of propargylbromide (1.55 mL, *Sigma Aldrich*: P51001, 80 % in toluene, 14.4 mmol, 1.00 eq.) in dry THF (15 mL) is added dropwise. When the reaction mixture starts to reflux, it is cooled to 0 °C. After complete addition of the bromide, the reaction mixture is stirred for 1 h at room temperature. Full conversion is assumed, and the crude material is directly used in the subsequent step.

2 (500 mg, 2.96 mmol, 1.00 eq.) is dissolved in dry THF (8 mL) and 5.30 mL (3.84 mmol, 1.30 eq.) of previously synthesized *Grignard* reagent is added. The mixture is stirred for 2 h at room temperature and then poured into ice water. Saturated aqueous NH_4Cl solution (20 mL) is added to dissolve the precipitate and the organic layer is separated. The aqueous layer is extracted with Et_2O (3 × 30 mL). Combined organic extracts are washed with brine (2 × 20 mL) and dried over Na_2SO_4. After evaporation of the solvent the residue is purified by column chromatography (EtOAc 100 %) and HPLC to obtain 34.0 mg (0.16 mmol, 5 %) of the desired probe **Metro-P2** as a viscous orange oil.

TLC: R_f = 0.41 (EtOAc) [UV].

^{1}H NMR (300 MHz, $CDCl_3$) δ (ppm) = 2.18 (t, ^4J = 2.7 Hz, 1H), 2.56 – 2.60 (m, 2H), 2.72 (s, 3H), 4.14 – 4.36 (m, 2H, H-8) 4.77 (d, ^3J = 11.8 Hz, 1H), 8.06 (s, 1H).

^{13}C NMR (75 MHz, $CDCl_3$) δ (ppm) = 14.6, 25.4, 50.7, 69.0, 72.3, 78.5, 132.7, 140.2, 152.1.

HR-MS (ESI): *m/z* = calc. $[M+H]^+$: 210.0878, found: 210.0874.

(**4**) (2-methyl-5-nitro-1-((prop-2-yn-1-yloxy)methyl)-*1H*-imidazole) - **Metro-P3**

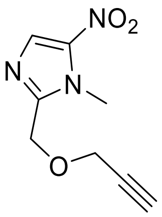

Metro-P3

To a suspension of (2-methyl-5-nitro-*1H*-imidazol-1-yl)methanol (250 mg, 1.59 mmol, 1.00 eq.) and Cs_2CO_3 (1.20 g, 3.91 mmol, 2.30 eq.) in THF (10 mL) is added propargyl bromide solution (80 % in toluene, *Sigma Aldrich*: P51001, 0.40 mL, 3.66 mmol, 1.20 eq.) and heated to 70 °C for 96 h. The reaction mixture is cooled to room temperature and the solvent is removed *in vacuo*. The crude product is further purified performing column chromatography (hexane/EtOAc 1:1) and high-performance liquid chromatography (HPLC) to obtain 136.5 mg of the desired probe **Metro-P3** (0.70 mmol, 44 %) as a white solid.

TLC: R_f = 0.36 (EtOAc) [UV].

^{1}H NMR (300 MHz, $CDCl_3$) δ (ppm) = 2.51 (t, ^4J = 2.4 Hz, 1H), 4.05 (s, 3H), 4.25 (d, ^4J = 2.4 Hz, 2H), 4.79 (s, 2H), 7.97 (s, 1H).

^{13}C NMR (75 MHz, $CDCl_3$) δ (ppm) = 34.2, 58.5, 63.2, 76.2, 78.2, 129.6, 147.8.

HR-MS (ESI): *m/z* = calc. $[M+H]^+$: 196.0717, found: 196.0715.

Analytical data is in accordance with literature.[8]

(**5**) (2-methyl-5-nitro-1-(prop-2-yn-1-yl)-*1H*-imidazole) - **Metro-P4**

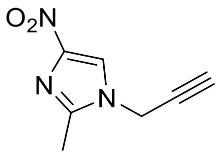

Metro-P4

The **Metro-P4** was synthesized *via* a S_N2 substitution reaction starting from the nitroimidazole compound **6** according to the literature procedure by *Bejot et al.*[9]

To a suspension of 2-methyl-4/5-nitro-*1H*-imidazole **6** (50.0 mg, 0.39 mmol, 1.00 eq.) and K_2CO_3 in DMF (2 mL) is added propargyl bromide solution (80 % in toluene, *Sigma Aldrich*: P51001, 0.05 mL, 0.47 mmol, 1.20 eq.) and the mixture is heated to 50 °C for 5 h and then stirred over night at room temperature. The reaction is quenched with *d*H_2O (5 mL) and diluted with EtOAc (20 mL). The organic phase is separated, and the aqueous phase is extracted with EtOAc (3 × 20 mL). Combined organic layers are washed with brine (20 mL), dried over anhydrous Na_2SO_4 and the solvent is evaporated *in vacuo*. The crude product is further purified performing column chromatography (hexane/EtOAc 1:1) to obtain 49.3 mg (0.30 mmol, 76 %) of the probe **Metro-P4** as a mixture of 4- and 5-nitroimidazole regioisomers (80:20) as a white solid.

$\mathbf{R_f}$ (hexane/EtOAc 1:1) = 0.36 [UV].

^{1}**H NMR** (400 MHz, $CDCl_3$) δ (ppm) = 2.40 (t, ^{4}J = 2.5 Hz, 1H), 2.49 (s, 3H), 2.56 (s, 3H), 2.60 (t, ^{4}J = 2.6 Hz, 1H), 4.70 (d, ^{4}J = 2.6 Hz, 2H), 5.15 (d, ^{4}J = 2.5 Hz, 2H), 7.78 (s, 1H), 7.86 (s, 1H).

^{13}**C NMR** (101 MHz, $CDCl_3$) δ (ppm) = 13.50, 36.94, 76.49, 76.84, 117.61, 119.54, 144.54.

LR-MS: *m/z* = calc. $[M+H]^+$: 166.06, found: 166.11.

(**7**) (1-(2-Methoxyethyl)-2-methyl-5-nitro-1*H*-imidazole) - **MF-01**

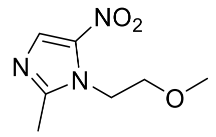

MF-01

Cs_2CO_3 (1.71 g, 5.28 mmol, 4.50 eq.) is added to a metronidazole (200 mg, 1.17 mmol, 1.00 eq.) dissolved in THF (10 mL) and stirred at room temperature for 30 min. Methyl iodide (*Sigma Aldrich*: I8507, 582 µL, 1.33 g, 9.36 mmol, 8.00 eq.) is added and the solution is stirred at room temperature for 21 h. The solvent is removed under reduced pressure, the remaining residue is dissolved in EtOAc and subsequently filtered. The crude product is purified by column chromatography (hexane/EtOAc = 1/1) and HPLC to afford **MF-01** (28.4 mg, 153 µmol, 13 %) as a white solid.

TLC: R_f = 0.35 (EtOAc) [UV].

HPLC: t_R = 5.0 min

^{1}H-NMR (400 MHz, $CDCl_3$): δ [ppm] = 2.75 (s, 3H), 3.30 (s, 3H), 3.73 (t, ^3J = 4.8 Hz, 2H), 4.59 (t, ^3J = 4.8 Hz, 2H), 8.07 (s, 1H).

^{13}C-NMR (101 MHz, $CDCl_3$): δ [ppm] = 13.6, 47.6, 59.4, 70.7, 126.9, 136.6, 150.8.

HR-MS (ESI): *m/z* = calc. $[M+H]^+$: 186.0873, found: 186.0871.

(**8**) (2-(Methoxymethyl)-1-methyl-5-nitro-1*H*-imidazole) - **MF-02**

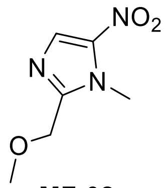

MF-02

Cs_2CO_3 (1.04 g, 3.18 mmol, 2.50 eq.) is added to 1-methyl-5-nitro-*1H*-imidazol-2-yl)methanol (200 mg, 1.27 mmol, 1.00 eq.) dissolved in THF (10 mL) and stirred at room temperature for 30 min. Methyl iodide (*Sigma Aldrich*: I8507, 560 µL, 1.26 g, 8.89 mmol, 7.00 eq.) is added and the solution is stirred at room temperature for 21 h. The solvent is removed under reduced pressure, the remaining residue is dissolved in EtOAc and then filtered. The crude product is purified by column chromatography (hexane/EtOAc = 1/1) and HPLC to afford **MF-02** (84.8 mg, 495 µmol, 39 %) as a white solid.

TLC: R_f = 0.48 (EtOAc) [UV].

HPLC: t_R = 6.6 min.

^{1}H-NMR (400 MHz, $CDCl_3$): δ [ppm] = 3.41 (s, 3H), 4.04 (s, 3H), 4.65 (s, 2H), 7.98 (s, 1H).

^{13}C-NMR (101 MHz, $CDCl_3$): δ [ppm] = 34.4, 58.9, 66.3, 130.2, 148.5, 157.3.

(**9**) (1-(2-ethoxyethyl)-2-methyl-5-nitro-1H-imidazole) - **MF-03**

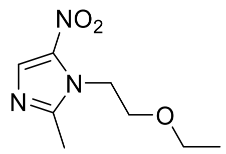

MF-03

Metronidazole (200 mg, 1.17 mmol, 1.00 eq.) and Cs_2CO_3 (457 mg, 1.40 mmol, 1.20 eq.) are suspended in dry THF (5 mL). Ethyl bromide (*TCI*: B0588, 0.20 mL, 306 mg, 2.80 mmol, 2.40 eq.) is slowly added to the suspension and heated to reflux. Over a period of 60 h, ethyl bromide (612 mg, 5.60 mmol, 4.80 eq.) and caesium carbonate (380 mg, 1.16 mmol, 1.00 eq.) are additionally added. The suspension is quenched with water (20 mL). The reaction mixture is extracted with ethyl acetate (3 × 20 mL). The combined organic layers are washed with brine (20 mL), dried over anhydrous Na_2SO_4 and the solvent is evaporated *in vacuo.* The crude product is further purified performing column chromatography (hexane/EtOAc = 1:5) to obtain an orange-brown solid (37.8 mg, 0.19 mmol, 16 %).

TLC: R_f = 0.30 (hexane/EtOAc 1:5) [UV].

^{1}H-NMR (400 MHz, $CDCl_3$): δ [ppm] = 1.10 (t, ^3J = 7.0 Hz, 3H), 2.58 (s, 3H), 3.41 (q, ^3J = 7.0 Hz, 2H), 3.73 (t, ^3J = 5.0 Hz, 2H), 4.51 (t, ^3J = 5.0 Hz, 2H), 7.98 (s, 1H).

^{13}C-NMR (100 MHz, $CDCl_3$): δ [ppm] = 14.4, 15.1, 47.1, 67.1, 69.1, 131.6, 138.4, 151.7.

HR-MS (ESI): *m/z* = calc. $[M+H]^+$: 200.1030, found: 200.1029.

(**10**) 2-(Ethoxymethyl)-1-methyl-5-nitro-1H-imidazole - **MF-04**

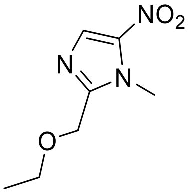

MF-04

Cs_2CO_3 (2.49 g, 7.64 mmol, 4.00 eq.) and tetrabutylammonium iodide (*Sigma Aldrich*: 140775, TBAI, 317 mg, 0.45 mol %) are added to (1-methyl-5-nitro-*1H*-imidazol-2-yl)methanol (*BLDpharm*: BD43612, 300 mg, 1.91 mmol, 1.00 eq.) dissolved in THF (10 mL) and stirred at room temperature for 30 min. Ethyl bromide (0.56 mL, 7.64 mmol, 4.00 eq.) is added and the solution is stirred at reflux for 21 h. The reaction mixture is filtered, and the solvent is evaporated *in vacuo*. The crude product is dissolved in EtOAc, filtered, and purified by column chromatography (hexane/EtOAc = 1:2) followed by HPLC purification. **MF-04** is obtained pure as a yellow oil (80.0 mg, 1.11 mmol, 58 %).

TLC: R_f = 0.44 (hexane/EtOAc 2:1) [UV].

HPLC: t_R = 7.7 min

^{1}H NMR (400 MHz, $CDCl_3$) δ [ppm] = 1.22 (t, ^3J = 7.1 Hz, 3H), 3.57 (q, ^3J = 7.1 Hz, 2H), 4.04 (s, 3H), 4.66 (s, 2H), 7.95 (s, 1H).

^{13}C NMR (100 MHz, $CDCl_3$) δ = 15.1, 33.9, 64.7, 66.8, 130.8, 139.7, 149.0.

HR-MS (ESI): *m/z* = calc. $[M+H]^+$: 186.0873, found: 186.0872.

(**11**) (2-methyl-5-nitro-1-(2-propoxyethyl)-*1H*-imidazole) - **MF-05**

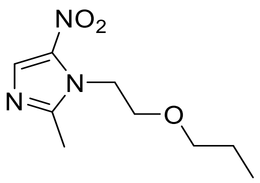

MF-05

Cs_2CO_3 (1.14 g, 3.51 mmol, 4.99 eq.) is added to metronidazole (150 mg, 876 µmol, 1.00 eq.) dissolved in THF (10 mL) and stirred at room temperature for 30 min. Propyl iodide (*Sigma Aldrich*: 171883, 0.85 mL, 8.76 mmol, 10.0 eq.) is added and the solution is stirred at reflux overnight. The reaction mixture is filtered, and the solvent is evaporated *in vacuo*. The crude product is purified by column chromatography (hexane/EtOAc = 1:2) to afford **MF-05** (38.5 mg, 184 µmol, 21 %) pure as a yellow oil.

TLC: R_f = 0.23 (hexane/EtOAc 1:2) [UV].

^{1}H NMR (400 MHz, $CDCl_3$) δ [ppm] = 0.83 (t, ^3J = 7.1 Hz, 3H), 1.49 (ps, ^3J = 7.1 Hz, 2H), 2.58 (s, 3H), 3.31 (t, ^3J = 7.1 Hz, 2H), 3.73 (t, ^3J = 4.5 Hz, 2H), 4.52 (t, ^3J = 4.5 Hz, 2H), 7.99 (s, 1H).

^{13}C NMR (100 MHz, $CDCl_3$) δ [ppm] = 10.6, 14.4, 22.9, 47.1, 69.3, 73.4, 131.9, 138.3, 151.6.

HR-MS (ESI): *m/z* = calc. $[M+H]^+$: 214.1186, found: 214.1184.

(**12**) (1-methyl-5-nitro-2-(propoxymethyl)-1*H*-imidazole) - **MF-06**

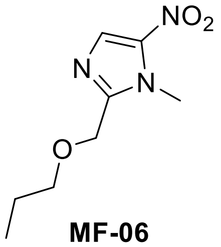

MF-06

Cs_2CO_3 (3.32 g, 7.64 mmol, 4.00 eq.) is added to (1-methyl-5-nitro-*1H*-imidazol-2-yl)methanol (*BLDpharm*: BD43612, 400 mg, 2.55 mmol, 1.00 eq.) dissolved in THF (10 mL) and stirred at room temperature for 30 min. Propyl iodide (*Sigma Aldrich*: 171883, 1.59 mL, 15.3 mmol, 6.00 eq.) is added and the solution is stirred at reflux overnight. The reaction mixture is filtered, and the solvent is evaporated *in vacuo*. The crude product is purified by column chromatography (hexane/EtOAc = 1:2) to obtain **MF-06** pure as a yellow oil (57.6 mg, 281 µmol, 11 %).

TLC: R_f = 0.57 (hexane/EtOAc 1:3) [UV].

^{1}H NMR (400 MHz, $CDCl_3$) δ [ppm] = 0.91 (t, ^3J = 7.2 Hz, 3H), 1.61 (ps, ^3J = 7.2 Hz, 2H), 3.44 (t, ^3J = 7.2 Hz, 2H), 4.02 (s, 3H), 4.62 (s, 2H), 7.93 (s, 1H).

^{13}C NMR (100 MHz, $CDCl_3$) δ [ppm] = 10.6, 22.9, 33.8, 65.3, 73.0, 131.6, 149.2.

HR-MS (ESI): *m/z* = calc. $[M+H]^+$: 200.1030, found: 200.1028.

(**13**) (1-(2-(allyloxy)ethyl) -2-methyl-5-nitro-1H-imidazole) - **MF-07**

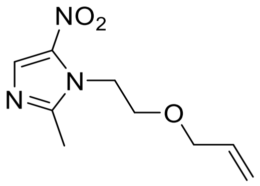

MF-07

Metronidazole (200 mg, 1.17 mmol, 1.00 eq.) and Cs_2CO_3 (762 mg, 2.34 mmol, 2.00 eq.) are suspended in dry THF (10 mL). To this suspension, allyl bromide (*TCI*: B0643, 0.30 mL, 2.80 mmol, 3.00 eq.) is slowly added and heated to 70°C. After 23 h, the suspension is quenched with addition of water (20 mL). The reaction mixture is extracted with ethyl acetate (3 × 20 mL). The combined organic layers are washed with brine (20 mL), dried over anhydrous Na_2SO_4 and the solvent is evaporated *in vacuo.* The crude product is further purified performing column chromatography (hexane/EtOAc = 1:5) to obtain **MF-07** pure as a gold-brown solid (74.5 mg, 0.35 mmol, 30 %).

TLC: R_f = 0.40 (hexane/EtOAc 1:5) [UV].

^{1}H NMR (400 MHz, $CDCl_3$) δ [ppm] = 2.57 (d, ^3J = 2.6 Hz, 3H), 3.75 (t, ^3J = 5.1 Hz, 2H), 3.84 – 3.93 (m, 2H), 4.52 (t, ^3J = 5.1 Hz, 2H), 5.09 – 5.20 (m, 2H), 5.66 – 5.81 (m, 1H), 7.98 (d, ^3J = 2.6 Hz, 1H).

^{13}C-NMR (100 MHz, $CDCl_3$): δ [ppm] = 14.5, 46.9, 68.7, 72.3, 117.7, 132.0, 133.8, 138.4, 151.7.

HR-MS (ESI): *m/z* = calc. $[M+H]^+$: 212.1030, found: 212.1030.

(**14**) (2-((allyloxy)methyl)-1-methyl-5-nitro-1*H*-imidazole) - **MF-08**

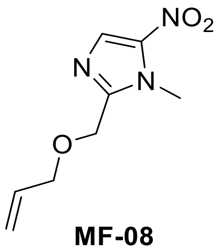

MF-08

Cs_2CO_3 (1.56 g, 4.77 mmol, 3.00 eq.) is added to (1-methyl-5-nitro-*1H*-imidazol-2-yl)methanol (*BLDpharm*: HMMNI, 250 mg, 1.59 mmol, 1.00 eq.) dissolved in THF (12 mL) and stirred at room temperature for 30 min. Allyl iodide (*Sigma Aldrich*: 238325, 0.44 mL, 4.77 mmol, 3.00 eq.) is added and the solution is stirred at reflux for 3 days. The reaction mixture is filtered, and and the solvent is evaporated *in vacuo*. The crude product is purified by column chromatography (hexane/EtOAc = 1:1) and HPLC to obtain **MF-08** pure as a colourless oil (138 mg, 700 µmol, 44 %).

TLC: R_f = 0.40 (hexane/EtOAc 1:1) [UV].

^{1}H NMR (400 MHz, $CDCl_3$) δ [ppm] = 4.02 – 4.06 (m, 5H, H-6), 4.66 (s, 2H), 5.20 – 5.37 (m, 2H), 5.77 – 5.96 (m, 1H), 7.95 (s, 1H).

^{13}C NMR (100 MHz, $CDCl_3$) δ [ppm] = 33.9, 64.1, 72.0, 118.8, 131.0, 133.3, 139.7, 148.8.

HR-MS (ESI): *m/z* = calc. $[M+H]^+$: 198.0873, found: 198.0872.

(**15**) (2-methyl-1-(2-((3-methylbut-2-en-1-yl)oxy)ethyl)-5-nitro-1H-imidazole) - **MF-09**

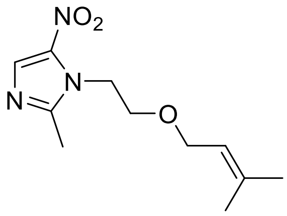

MF-09

Metronidazole (200 mg, 1.17 mmol, 1.00 eq.) and Cs_2CO_3 (762 mg, 2.34 mmol, 2.00 eq.) are suspended in dry THF (9 mL). 3,3-dimethylallyl bromide (*Sigma Aldrich*: 249904, 0.40 mL, 3.51 mmol, 3.00 eq.) is slowly added to the reaction mixture and heated to reflux for 18 h. The suspension is quenched with water (20 mL) and extracted with ethyl acetate (3 × 20 mL). The combined organic layers are washed with brine (20 mL), dried over anhydrous Na_2SO_4 and the solvent is evaporated *in vacuo.* The crude product is further purified performing column chromatography (hexane/EtOAc = 1:5) and HPLC to obtain **MF-09** as a white solid (29.0 mg, 0.12 mmol, 10 %).

TLC: R_f = 0.42 (hexane/EtOAc 1:5) [UV].

^{1}H-NMR (400 MHz, $CDCl_3$): δ [ppm] = 1.59 (d, ^3J = 1.3 Hz, 3H), 1.70 (d, ^3J = 1.4 Hz, 6H), 1.76 (d, ^3J = 1.3 Hz, 3H), 2.52 (s, 3H), 2.62 (s, 3H), 3.72 (t, ^3J = 4.9 Hz, 2H), 3.88 (d, ^3J = 6.9 Hz, 2H), 4.47 (t, ^3J = 4.3 Hz, 2H), 4.51 (t, ^3J = 4.9 Hz, 2H), 4.56 – 4.63 (m, 4H), 5.05 – 5.16 (m, 1H), 5.25 – 5.40 (m, 1H), 7.97 (s, 1H), 8.00 (s, 1H).

^{13}C-NMR (100 MHz, $CDCl_3$): δ [ppm] = 14.1, 14.2, 18.0, 18.1, 25.7, 25.8, 45.4, 47.2, 65.3, 65.6, 67.7, 67.8, 117.5, 120.0, 130.1, 132.6, 138.0, 140.9, 151.1, 154.7.

HR-MS (ESI): *m/z* = calc. $[M+H]^+$: 240.1343, found: 240.1343.

(**16**) (1-butyl-2-methyl-5-nitro-1H-imidazole) - **MF-10**

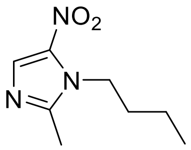

MF-10

1-Brombutane (*TCI*: B0560, 0.68 mL, 6.29 mmol, 4.00 eq.) is added to a solution of 2-methyl-4(5)-nitroimidazole (*Sigma Aldrich*: 136255, 200 mg, 1.57 mmol, 1.00 eq.) and Na_2CO_3 (334 mg, 3.15 mmol, 2.00 eq.) in DMF (10 mL) and heated to 60°C overnight. After cooling to room temperature, the reaction mixture is filtered. The solvent is removed under reduced pressure. After purification *via* column chromatography (hexane/EtOAc, 1:1), the addition of water causes a solid to precipitate, which was dried under vacuum to yield 206 mg (70.7 mmol, 71%) **MF-10** as an off-white solid.

TLC R_f = 0.33 (hexane/EtOAc, 1:1) [UV].

^{1}H-NMR (400 MHz, $CDCl_3$): δ [ppm] = 1.00 – 0.96 (t, 3H, ^3J = 7.34 Hz), 1.43 – 1.34 (m, 2H), 1.81 – 1.74 (m, 2H), 2.43 (s, 3H), 3.91-3.87 (t, 2H, ^3J = 7.34 Hz), 7.67 (s, 1H).

^{13}C-NMR (100 MHz, $CDCl_3$): δ [ppm] = 13.24, 13.62, 19.81, 32.41, 47.11, 119.49.

HRMS (ESI) *m/z* = calc. $[M+H]^+$: 184.10807 found: 184.10803.

(**17**) (1-pentyl-2-methyl-5-nitro-1*H*-imidazole) - **MF-11**

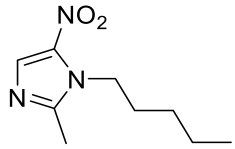

MF-11

1-Brompentane (0.79 ml, 6.29 mmol, 2.00 eq.) is added to a solution of 2-Methyl-4(5)-nitroimidazole (*Sigma Aldrich*: 136255, 400 mg, 3.15 mmol, 1.00 eq.) and Na_2CO_3 (1.33 g, 12.6 mmol, 4.00 eq.) in DMF (10 mL) and heated to 60°C for 4 h. After cooling to room temperature, the reaction mixture is filtered. The solvent was removed under reduced pressure. After purification *via* column chromatography (hexane/EtOAc, 1:6), the resulting oil is crystallized to yield 161 mg (0.81 mmol, 26%) **MF-11** as white crystals.

TLC R_f = 0.40 (hexane/EtOAc, 1:6) [UV].

^{1}H-NMR (400 MHz, $CDCl_3$): δ [ppm] = 0.92 (t, 3 H, ^3J = 7.05 Hz), 1.41 – 1.29 (m, 4 H), 1.83 – 1.75 (m, 2 H), 2.42 (s, 3 H), 3.89 (t, 2 H, ^3J = 7.33 Hz), 7.67 (s, 3 H).

^{13}C-NMR (100 MHz, $CDCl_3$): δ [ppm] = 13.26, 13.94, 22.28, 28.67, 30.13, 47.38, 119.47,144.67.

HRMS (ESI) *m/z* = calc. $[M+H]^+$: 198.12367 found: 198.12364.

1.3 Cloning

Primers

Table S 9: Primer sequences (except Δ*tpx* primers, for this, see 'generation of HpTpx Δ*tpx* knockout mutant') used in the present work.

Primer	Gene	5'-3' Sequence	Supplier
Gateway HpTpx FWD	*tpx*	ggggacaagtttgtacaaaaaagcaggctttcaaaaagttacttttaaagaagaaac	*Sigma*
Gateway HpTpx REV	*tpx*	ggggaccactttgtacaagaaagctgggtgctatttcaacacttttaaaagcg	*Sigma*
HpTpx C60A FWD	*tpx*	tagtttaaccggatcggtt**gc**tttgctccaagccaaacac	*Sigma*
HpTpx C60A REV	*tpx*	gtgtttggcttggagcaaa**gc**aaccgatccggttaaacta	*Sigma*
HpTpx C94A FWD	*tpx*	tgcctttttctcaagggcaaatt**gc**cggcgctgaagg	*Sigma*
HpTpx C94A REV	*tpx*	ccttcagcgccg**gc**aatttgcccttgagaaaaaggca	*Sigma*
HpGroEL insert	*groEL*	atggccaaagagatcaagttctctgatagcgcgcgtaaccttttgtttgagggtgttcgaca<br>actgcacgatgcggttaaagtaaccatggggccccggggccgtaatgttctgattcagaa<br>gtcctacggagcacctagcattaccaaagatggcgtgagtgtggcgaaagaaattgaatt<br>atcctgcccggtagcaaacatgggagcccagctggtaaaagaagttgcgtccaaaactg<br>ccgacgcagccggcgatggtaccactacagcgactgtgcttgcgtattccatatttaagga<br>agggctcagaaatatcaccgctggtgcgaatccaatcgaggtgaagcgcggtatggata<br>aggccgcagaggcaatcattaacgagctgaagaaagcctcaaaaaaggttggtggtaa<br>agaagaaatcacacaggtagcaaccatatctgcgaactcagatcataatattgggaagtt<br>aatcgcggatgctatggaaaaggtcggcaaggacggagttattaccgttgaggaagcca<br>agggtattgaggatgagttggatgttgttgaaggcatgcagtttgaccgggggtacttgtca<br>ccatactttgtaaccaatgcagaaaaaatgactgcgcagctggataatgcgtatatcttgct<br>gactgacaaaaaaatcagctcaatgaaagacatactgcctctcttggagaaaaccatga<br>aagaaggtaagcctctgttaattatcgcagaagatatcgaaggcgaagctctcacgaccc<br>tggtagtcaataagttacggggcgtcttaaacatcgcagctgtaaaggcgccagggtttgg<br>tgatcgtcgtaaagagatgctgaaagatattgcaatactgaccggcgggcaagttatttcc<br>gaggagctggggctcagtctggaaaacgccgaagttgaatttctggggaaggccggtag<br>aatcgtgatcgataaagataataccaccatcgtcgatggcaaaggtcattctcacgatgtg<br>aaagaccgggtcgctcagattaagacgcagatagcatcaacaacgagtgactatgaca<br>aagagaaacttcaggagcgactcgcgaaactgtcaggcggcgttgctgtcatcaaagtc<br>ggcgctgccagcgaagtggagatgaaagaaaaaaaggatcgcgtcgatgatgctttatc<br>tgcaacaaaagctgctgtggaagaaggcatagtgataggtggtggagcagcgctcatac<br>gagcggctcagaaggtacatttgaacttacatgatgacgaaaaagtgggttatgaaatcat<br>aatgcgcgcaataaaagccccgctggcgcagattgcgatcaacgcaggttatgacggtg<br>gtgttgtcgtgaatgaagtggaaaaacacgagggccactttggtttcaatgcatcaaacgg<br>caaatatgtcgacatgttcaaagaagggattattgatccgttaaaagtggagcgaattgca<br>ctgcagaatgctgtgtccgtttcgagtctgttgctgactacagaagctaccgtccatgaaata	*TwistBioscience*

		aaagaagaaaaagcggcccctgcaatgccggatatgggtggtatgggcggcatgggtg gcatgggtggcatgatggaaaatttatatttccagggg	

Cloning of recombinant HpGroEL in E. coli

pET-28a(+) plasmids with kanamycin resistance cassette encoding the gene *groEL* (Uniprot ID: P42383) with a TEV cleavage site before the C-terminal His tag were purchased from *TwistBioscience*. HpGroEl was ligated into pET-28a(+) using NcoI and XhoI restriction sites. Sequence of the insert is shown in **Table S9** and was verified by DNA sequencing (*Azenta*). The plasmid was transformed into chemically competent *E. coli* BL21 (DE3) cells for protein expression and purification.

Cloning of recombinant HpTpx in E. coli

gDNA extraction of *H. pylori* 26695 was performed with *GeneRabbit* DNA extraction Kit. *H. pylori tpx* gene (Uniprot ID: O25151) for recombinant protein expression in *E. coli* was amplified by polymerase chain reaction (PCR) according to **Table S10**. The PCR mixture contained 10 µL 5x Phusion HF buffer (NEB), 1 µL dNTP mix (10 mM), 1 µL forward primer (10 µM), 1 µL reverse primer (10 µM), 1 µL *H. pylori* gDNA (25 – 50 ng), 1.5 µL DMSO and 1 µL Phusion DNA Polymerase (NEB) and filled up to 50 µL with nuclease-free water. After verification of the reactions by an 1 % agarose gel, PCR Products were purified using a E.Z.N.A.® MicroElute Cycle Pure Kit (*Omega*) according to the manufacturers protocol.

Table S 10: Cycling parameters for tpx gene amplification PCR reactions.

Time	Temperature	Cycle
**30 s**	98 °C	
**10 s**	95 °C	35 ×
**30s**	50 °C	
**15 s**	72 °C	
**10 min**	72 °C	
∞	4 °C	

Cloning of N-terminal Strep-tagged tpx (HpTpx) was performed using the *Invitrogen* Gateway® cloning system with pDONR™201Kan (*Invitrogen*) as the donor vector and pET-55-DEST™Amp (*Invitrogen*) as the destination vectors. Protocols were adopted from *Invitrogen* (Thermo Fisher Scientific, "Gateway Cloning Protocols"). BP and LR reactions were transformed into *E. coli* TOP10 cells. After re-isolation of final plasmid DNA with plasmid Miniprep Kit (peqGOLD Plasmid Miniprep Kit II, *VWR* Peqlab) according to the manufacturer's instructions, correct insertion was checked with Sanger Sequencing (*Azenta*). Isolated plasmid DNA was re-transformed in competent *E. coli* BL21(DE3) cells (*Promega*) as the final expression strain.

QuikChange Site Directed Mutagenesis for Generation of HpTpx Point Mutations

Cloning of N-terminal Strep-tagged Hptpx was performed using the *Invitrogen* Gateway Technology as described above. Plasmid DNA of wildtype pET-55-DEST-HpTpx was purified using a plasmid Miniprep Kit (peqGOLD Plasmid Miniprep Kit II, *VWR Peqlab*) according to the manufacturer's instructions and used as template for single point mutations (C60A, C94A). For double mutations (C60A-C94A), plasmid DNA of pET-55-DEST-HpTpx with single point mutation (C60A) was isolated and used as template for site-directed mutagenesis. Point mutation primers (*Sigma*) were designed based on the HpTpx gene sequence according to the manufacturer's instructions (*Agilent*). Primer sequences are listed in **Table S9.** QuikChange site directed mutagenesis PCR reactions were performed using Phusion High-Fidelity DNA

Polymerase (*New England BioLabs*) using the cycle listed below (**Table S11**). The PCR mixture contained 10 µL GC buffer (NEB), 1 µL dNTP mix (10 mM), 1 µL forward primer (10 µM), 1 µL reverse primer (10 µM), 1 µL plasmid template (25 ng), 1.5 µL DMSO and 1 µL Phusion DNA Polymerase (NEB) and 33.5 µL ddH_2O.

Table S 11: Cycling parameters for QuikChange site-directed mutagenesis PCR reactions.

Time	Temperature	Cycle
**3 min**	98 °C	
**45 s**	95 °C	35 ×
**30 s**	60 or 68 °C	
**3 min**	72 °C	
**7 min**	72 °C	
∞	4 °C	

PCR product was digested with *DpnI* (1 µL CutSmart Buffer, 1 µL DpnI, 8 µL PCR reaction mixture) for 1 h at 37°C and subsequently transformed in *E. coli* XL1 blue cells for nick repair. After re-isolation of plasmid DNA with plasmid Miniprep Kit (peqGOLD Plasmid Miniprep Kit II, *VWR* Peqlab) according to the manufacturer's instructions, correct insertion of the desired point mutation(s) and overall sequence was checked with Sanger Sequencing (*Azenta*). Isolated plasmid DNA was re-transformed in competent *E. coli* BL21(DE3) cells (*Promega*) as the final expression strain.

Generation of H. pylori 26695 ∆tpx mutant

All primers used for generation of *H. pylori* 26695 ∆*tpx* are summarized in **Table S12**.

Table S 12: Primer sequences used for generation of *tpx* knockout mutants (Gibson assembly primers and PCR verification primers).

Primer	Sequence 5'→3'	
UF tpx rpsL fwd	**gacggtatcgat**ggtcaatgtaataagcatgc	for Gibson Assembly of pTpx_rpsL-cat
UF tpx rpsL rev	**atccatagttata**ggttaaatcctttctaattgaattg	
rpsL-cat tpx fwd	**aaggatttaacc**tataactatggattaaacactttttag	
rpsL-cat tpx rev	**tttaaggatttc**ttcagcaagtcttgtaattc	
LF tpx rpsL fwd	**aagacttgctgaa**gaaatccttaaaaggagggggc	
LF tpx rpsL rev	**ggtggcggccgc**cgcgagtccctgaaaccc	
backbone pOND708 tpx fwd	**tcagggactcgcg**gcggccgccaccgcggtg	
backbone pOND708 tpx rev	**tattacattgacc**atcgataccgtcgatcgaggggggc	
rpsL control fwd	ttggtgaacgaatgggaatg	Sequencing, Verification PCR
tpx control fwd	accttagaggctattaagttgctga	
tpx control rev	tggcgataagattccggacgct	

*__Bold:__ Overhang for Gibson Assembly

A deletion plasmid (pTpx_rpsL-cat) containing 0.5 – 0.7 kb homologous sequences to the up- and downstream region of the *tpx* gene (HP0927) was generated. Homologous flanks were amplified by PCR using Q5 polymerase (NEB) and primer pairs UF tpx rpsL fwd/rev and LF tpx rpsL fwd/rev. In the deletion plasmid, homologous regions flank a *rpsL-cat* cassette, that was amplified from plasmid pOND708[3] using primers rpsL-cat tpx fwd/rev. Chloramphenicolacetyltransferase (*cat*) confers resistance to chloramphenicol to bacteria that successfully integrated the deletion plasmid and *rpsL* allows for the use of the plasmid in a streptomycin-based counter selection system.[10] In addition, pOND708 was amplified to be used as a plasmid backbone using the primer pair backbone pOND708 tpx fwd/rev. PCR products were purified using Promega ReliaPrep and the plasmid was assembled using the NEB Gibson assembly kit. After transformation into *E. coli* DH5α, correct assembly of pTpx_rpsL-cat was verified by PCR using primers LF tpx rpsL rev /rpsL control fwd and by sequencing using primers LF tpx rpsL rev and UF tpx rpsL fwd. The deletion plasmid was transferred into *H. pylori* 26695 using the natural transformation competence of the bacterium and deletion mutants were selected on chloramphenicol (10 µg/ml) containing WC-DENT blood agar plates. Correct replacement of the *tpx* gene with *rpsL-cat* was verified by PCR using primers tpx control fwd/rev for several clones growing on the plate and the loss of protein expression was additionally proven by full proteome LC-MS/MS analysis (**Figure S26**).

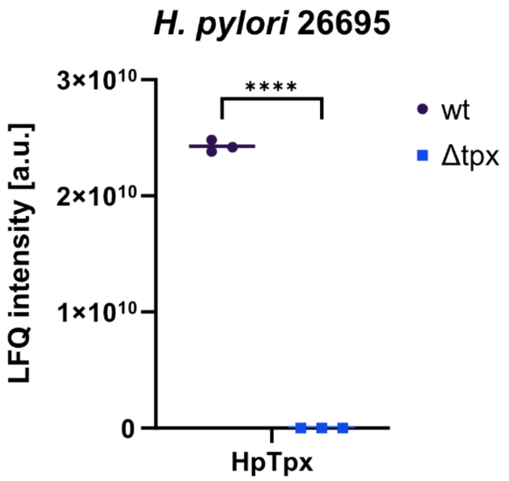

Figure S 26: Validation of successful cloning of *H. pylori* 26695 Δ*tpx* mutant exemplarily shown for ‘clone 1’. LFQ intensities of HpTpx (O25151, thiol peroxidase) after full proteome analysis of *H. pylori* 26695 wildtype and Δ*tpx* mutant. Experiment was performed in biological triplicates (n =3). Statistical significance was determined by a two-tailed Student’s t-test. ****: p<0.0001. Result of one picked colony/clone shown.

1.4 Gel-based Activity-based Protein Profiling (ABPP) in HeLa cells

Labeling and Lysis in HeLa cells

For gel-based ABPP, HeLa cells were seeded in 6-well plates and grown at 37 °C and 5 % CO_2 to 90 % confluency. Before labeling, DMEM medium was fully removed, and cells were washed with PBS (1 ml) once. To each well, 1 µL of **Metro-P1**, **Metro-P2**, **Metro-P3** (1000 × stock compound in DMSO) in 1 mL medium (DMEM + 2 mM *L*-glutamine without FCS) were added and incubated for 1 h at 37 °C (5 % CO_2). For control experiments, cells were treated with 0.1 % (v/v) DMSO. After removal of medium and washing with ice-cold PBS (1 mL) cells were lysed by adding 100 µL lysis buffer (50 mM Tris, pH = 7.5, 150 mM NaCl, 1 % (v/v) NP40, 0.1 % (w/v) sodium deoxycholate, 1 mM EDTA) to each well and incubated for 15 min on ice. Afterwards, cells were scraped off wells, transferred into 1.5 mL Eppendorf tubes and centrifuged (21 000 × g, 15 min, 4 °C) before *Click* chemistry was performed.

The samples were subjected to *Click* reaction by adding 1 µL rhodamine azide (10 mM in DMSO), 5 µL BTTAA (10 mM in DMSO), 2 µL $CuSO_4$ (50 mM in ddH_2O) and 2 µL NaAsc (100 mM in ddH_2O) to 90 µL of each sample and incubated for 1 h at r.t. *Click* reaction was quenched by adding 500 µL cold acetone (−80 °C), vortexed and stored overnight at −80 °C. Samples were centrifuged (6000 × g, 15 min, r.t.) and supernatant was aspirated. Cell pellet was resuspended in 65 µL PBS and 65 µL 2 × SDS running buffer under mild ultrasound sonication (10 s, 10 %, 5 × cycle) and protein bands were separated using SDS-Page and visualized by fluorescence detection described in ‘SDS-Page’.

1.5 SDS-Page

Stacking gels contained 4% (w/v) acrylamide (in 50 mM Tris, pH = 6.8) and resolving gels consisted of 12.5 or 15% (w/v) acrylamide (in 300 mM Tris, pH = 8.8). The gels were run in a Tris-glycine buffer (25 mM Tris, 192 mM glycine, 0.1% (w/v) SDS, pH = 8.3). Prior to loading on gel, samples were incubated at 96 °C for 5 min. For gel electrophoresis, 25 or 40 µL of each condition were loaded onto the gel. Roti®-Mark STANDARD (RM; *Carl Roth*) or PeqGOLD protein marker I (PM; VWR) and BenchMark^{TM} Fluorescent protein standard (FM; *Thermo Fisher*) were used as markers to determine the respective protein masses. Gels were run for 2.5 h with 150 V in an EV265 Consort (*Hoefer*). Fluorescence was detected with a Fujifilm LAS-4000 luminescent image analyser with a Fujinon VRF43LMD3 lens and a 575DF20 filter. For determination of relative protein amounts, gels were stained with Coomassie and scanned on a LAS-4000 (*Fujifilm*).

1.6 BCA Assay

Protein concentrations were determined using the Pierce BCA protein assay kit (*Thermo Fisher Scientific*, Pierce Biotechnology). Standard calibration curve (BSA, 0 µg/mL to 400 µg/mL, 50 µL) and lysate samples (diluted 1:10 in 50 µL PBS) were pipetted into a flat bottom 96-well plate in triplicates. 100 µL of the BCA working solution (15 parts reagent 1, 1 part reagent 2) were added to the samples and incubated for 15 min at 60°C prior to measuring the absorption at 492 nm on a Tecan Infinite M200Pro Plate reader. The lysate concentration in bacteria was adjusted to a final protein amount of 250 µg protein in 500 µL (0.5 mg/mL) prior to *Click* chemistry.

1.7 Desalting and Filtration

H. pylori samples were desalted using SepPak® C18 cartridges (50 mg, *Waters*). Prior to desalting, the columns were washed with elution buffer (80 % MeCN, 0.5 % formic acid (FA), 2 × 1 mL) and equilibrated with 0.1 % trifluoroacetic acid (TFA) in ddH_20 (3 × 1 mL). The supernatant of each sample was loaded onto the columns and the beads were washed with 0.1 % TFA in ddH_20 (1 mL), centrifuged (13 000 × g, 3 min, r.t.), and supernatant loaded once more onto each column before washing with 0.1 % TFA in ddH_20 (2 × 1 mL) and 0.5 % FA in ddH_20 (500 µL). Peptides were eluted into tubes with elution buffer (80 % MeCN, 0.5 % FA, 1 × 500 µL, 1 × 250 µL) while vacuum was applied during the last step. Samples were evaporated using a speed vac (Concentrator Plus, *Eppendorf*). The dried peptides were dissolved in 40 µL 1 % FA in ddH_20, vortexed and sonicated (3 × 5 min). PVDF filters (0.22 µM, *Merck Millipore*) were washed with 300 µL 1 % FA in ddH_20 (13 000 × g, 1 min, r.t.). Samples were filtered by centrifugation (13 000 rpm, 1 min, r.t.) and transferred into MS vials (*Thermo Fisher*).

Desalting of *S. pseudintermedius* and *S. schleiferi* samples was performed using pre-equilibrated stage tips containing two layers of styrenedivinylbenzene-reverse phase sulfonate (SDB-RPS) disks (Empore, 3M). The stage tips were equilibrated with 150 µL wash buffer 1 (1% TFA in isopropanol) before loading the samples. Samples were loaded for 10 min at 500 x g, followed by washing with 150 µL wash buffer 1 for 10 min at 800 x g, and another wash with 150 µL wash buffer 2 (0.2% TFA in H2O). Peptides were eluted with 50 µL elution buffer (1% ammonia, 80% acetonitrile) for 5 min at 300 x g, followed by 5 min at 800 x g. Eluted peptide samples were dried using a centrifugal evaporator (Concentrator Plus, Eppendorf), following reconsititution in 35 µL of 1% FA.

1.8 MS Measurement and Data Analysis

H. pylori samples were analyzed via HPLC-MS/MS using an UltiMate 3000 nano HPLC system (*Dionex*) equipped with Acclaim C18 PepMap100 trap column (75 µm ID × 2 cm, Acclaim, *ThermoFisher*) and Aurora Ultimate™ (1st generation, 20 cm nanoflow UHPLC compatible, *ionopticks*) separation columns coupled to a Q Exactive Plus Orbitrap Mass Spectrometer (*Thermo Fisher Scientific*) constantly heated to 40 °C. Samples were loaded onto the trap column and washed with TFA (0.1% in ddH_2O). The subsequent separation was carried out with a flow rate of 400 nL/min using buffer A (0.1% FA in ddH_20) and buffer B (0.1% FA in acetonitrile). The separation column was heated to 40 °C. The analysis started with washing for 7 min with 5% buffer B for desalting followed by a gradient from 5% to 22% buffer B over 105 min, a second gradient from 22% B to 32% B within 10 min, and a final increase to 90% B in 10 min. Isocratic washing with 90% B was performed for 10 min, then decreased to 5% in 0.1 min and held at 5% for additional 9.9 min for re-equilibration.

The Q Exactive Plus mass spectrometer was run in a TOP10 data-dependent mode. In the orbitrap, full MS scans were collected in a scan range of 300-1500 m/z at a resolution of 140 000 and an AGC target of 3e6 with 80 ms maximum injection time. The TOP12 peaks were selected for MS2 scan with a minimum AGC target of 1e3 and isotope exclusion and dynamic exclusion (exclusion duration: 60 s) enabled. Peaks with unassigned charges or a charge of +1 were excluded. Peptide match was "preferred". MS2 spectra were collected at a resolution of 17 500 aiming at an AGC target of 1e5 with a maximum injection time of 100 ms. Isolation was conducted in the quadrupole using a window of 1.6 m/z. Fragments were generated using higher-energy collision-induced dissociation (HCD, normalized collision energy: 27%) and finally detected in the orbitrap. The lock-mass ion 445.12002 from ambient air was used for real-time mass calibration on the Q Exactive Plus.

S. schleiferi and *S. pseudintermedius* samples were measured and online-separated using an UltiMate 3000 nano HPLC system (*Dionex*) coupled to a *Bruker* timsTOF Pro mass spectrometer via a CaptiveSpray nano-electrospray ion source and *Sonation* column oven. Peptides were first loaded on the trap column (Acclaim PepMap 100 C18, 75 µm ID x 2 cm, 3 µm particle size, *Thermo Scientific*), washed with 0.1% formic acid in water for 7 min at 5 µL/min and subsequently transferred to the separation column (*IonOpticks* Aurora C18 column, 25 cm × 75 µm, 1.7 µm) and separated over a 60 min gradient from 5% to 28% B, then to 40 % B over 13 min, followed by 10 min at 95% before re-equilibration and at a flow rate of 400 nL/min. The mobile phases A and B were 0.1 % (v/v) formic acid in water and 0.1% (v/v) formic acid in acetonitrile, respectively.

The timsTOF Pro was operated in data-independent dia-PASEF mode with the dual TIMS analyser operating at equal accumulation and ramp times of 100 ms each with a set $1/K_0$ ion mobility range from 0.60 to 1.60 V × s/cm^2 for MS1 scans. The dia-PASEF settings for fragmentation were set to a mass range of 400 to 1201 m/z and an ion mobility range of 0.60 to 1.43 V × s × cm^{-2}. Two ion mobility isolation windows were performed per dia-PASEF scan with 26 m/z window widths. A total of 32 isolation windows with 1 m/z overlaps to cover the mass range were used resulting in 16 dia-PASEF scans per MS1 scan and an estimated total cycle time of 1.80 s (see Table). The collision energy was ramped linearly as a function of the mobility from 59 eV at $1/K_0$ = 1.3 V × s × cm^{-2} to 20 eV at $1/K_0$ = 0.85 V × s × cm^{-2}. TIMS elution voltages were calibrated linearly to obtain the reduced ion mobility coefficients ($1/K_0$) using three Agilent ESI-L Tuning Mix ions (*m/z* 622, 922 and 1,222) spiked on the CaptiveSpray Source inlet filter.

MS Type	Scan	Start IM [1/K0]	End IM [1/K0]	Start Mass [m/z]	End Mass [m/z]
MS1	0	0.6	1.6	100	1700
dia-PASEF	1	0.9	1.2	800	826
dia-PASEF	1	0.6	0.9	400	426
dia-PASEF	2	0.92	1.22	825	851
dia-PASEF	2	0.62	0.92	425	451
dia-PASEF	3	0.93	1.23	850	876
dia-PASEF	3	0.63	0.93	450	476
dia-PASEF	4	0.95	1.25	875	901
dia-PASEF	4	0.65	0.95	475	501
dia-PASEF	5	0.96	1.26	900	926
dia-PASEF	5	0.66	0.96	500	526
dia-PASEF	6	0.98	1.28	925	951
dia-PASEF	6	0.68	0.98	525	551
dia-PASEF	7	0.99	1.29	950	976
dia-PASEF	7	0.69	0.99	550	576
dia-PASEF	8	1.01	1.31	975	1001
dia-PASEF	8	0.71	1.01	575	601
dia-PASEF	9	1.02	1.32	1000	1026
dia-PASEF	9	0.72	1.02	600	626
dia-PASEF	10	1.04	1.34	1025	1051
dia-PASEF	10	0.74	1.04	625	651
dia-PASEF	11	1.06	1.36	1050	1076
dia-PASEF	11	0.76	1.06	650	676
dia-PASEF	12	1.07	1.37	1075	1101
dia-PASEF	12	0.77	1.07	675	701
dia-PASEF	13	1.09	1.39	1100	1126
dia-PASEF	13	0.79	1.09	700	726
dia-PASEF	14	1.1	1.4	1125	1151
dia-PASEF	14	0.8	1.1	725	751
dia-PASEF	15	1.12	1.42	1150	1176
dia-PASEF	15	0.82	1.12	750	776
dia-PASEF	16	1.13	1.43	1175	1201
dia-PASEF	16	0.83	1.13	775	801

Data was acquired using Xcalibur software version 3.1 sp3 and proteomics raw data were analysed using MaxQuant[11] (ver. 1.6.2.10) which uses the Andromeda search engine.[12] Settings were default except for LFQ-quantification and match between runs, which were activated during search. The parameters can be found attached in the appendix. Perseus 2.0.10.0 or 2.0.11.0ima was used for analysis.[13] Data was filtered as following: Potential

contaminants' were removed as well as 'reverse peptides' and 'only identified by site'. LFQ intensities were log_2 transformed. Rows were annotated in groups – DMSO (control) and different probe treatments. Rows were filtered for 2 valid values in at least one group. Missing values were imputed from normal distribution. Annotations derived from data banks (Uniprot[14]) were added. Each condition was performed in biological triplicates (n = 3) and volcano plots were created based on two-sided two sample Student's t-tests.

1.9 Full Proteome Analysis

Sample preparation for H. pylori 26695 Δtpx verification

For 20 mL of *H. pylori 26695* or *Δtpx,* cultures were inoculated from WC Dent plates and grown in BHI + 10 % FCS (37°C, 140 rpm, microaerophilic conditions) until stationary phase was reached (*H. pylori* growth was followed time-dependently *via* OD_{600} measurements and confirmed *via Stuart's* urease test). The cells were harvested (6 000 rpm, 10 min, r.t.), washed with PBS twice (500 µL, 6 000 rpm, 10 min, 4°C) and frozen at −80°C until lysis.

Lysis and Protein Precipitation

For lysis, cell pellet was resuspended in RIPA buffer with protease inhibitor (200 µL, 50 mM Tris-Cl, 150 mM NaCl, 1mM EGTA, 1 % Igepal, 0.25 % sodium deoxycholate, pH = 7.4, addition of 1 tablet of protease inhibitor (*Roche* cOmplete Tablets Mini EDTA-free) per 10 mL RIPA buffer), incubated on ice for 5 min followed by sonication for 10 min in a sonication bath (*Bandelin* Sonorex Super RK). Lysate is clarified by centrifugation (21 000 × g, 30 min, 4°C). For full proteome analysis, samples were adjusted to equal protein amounts by BCA assay (see 'MS-based ABPP') and subsequently precipitated with ice-cold acetone (4 × volumes) at −20°C overnight. Precipitated proteins are pelletized (10 000 rpm, 15 min, 4 °C) and supernatant is discarded. For washing, protein pellet is resuspended in 0.5 mL cold methanol (−80 °C) by mild sonication (10 s, 10 % intensity) and centrifuged (21 000 g, 10 min, 4 °C) twice.

Reduction, Alkylation and Digest

Each protein pellet is resuspended in 200 µL denaturation buffer (7 M urea, 2 M thiourea in 20 mM HEPES buffer, pH = 7.5), then reduced with 10 mM Tris(2-carboxyethyl)phosphine hydrochloride (TCEP; 600 rpm, 1 h, 37°C) and subsequently alkylated with 10 mM iodoacetamide (IAA; 600 rpm, 30 min, r.t.). Alkylation was quenched by the addition of 10 mM dithiothreitol (DTT; 600 rpm, 30 min, r.t.). For digestion, LysC (1:200 enzyme:protein ratio; 0.5 µg/µl) was added to each sample and incubated for 2 h at r.t. with shaking at 600 rpm. 50 mM triethylammonium bicarbonate (TEAB, 600 µL) was added and the pH value was checked to be above 8. Trypsin (1:100 enzyme:protein ratio; 0.5 µg/µL in 50 mM acetic acid, *Promega*) was added to digest the samples for 16 h (37 °C, 600 rpm) and quenched the next day by acidifying with formic acid (10 µL, final pH below 3.0). Samples were centrifuged (13 000 rpm, 3 min, r.t.) before proceeding to the desalting step and filtration step according to procedure described in 'MS-based ABPP'.

LC-MS/MS Measurement

MS analysis was performed on an Orbitrap Fusion mass spectrometer coupled to an Ultimate3000 nano-HPLC via a *Nanospray Flex Ion Source* (ThermoFisher Scientific). Samples were loaded onto the trap column (AcclaimPepMap 100 C18 (75 µm×2 cm) trap/ flow rate: 5 µL/min, 0.1 % TFA) and separated on the Aurora Ultimate™ columns (2nd generation, 75 µm×25 cm, *ionopticks*). Both columns were constantly heated to 40 °C. The subsequent

separation was carried out with a flow rate of 400 nL/min using buffer A (0.1% FA in ddH_20) and buffer B (0.1% FA in acetonitrile). The analysis started with washing for 7 min with 5% buffer B for desalting followed by a gradient from 5% to 28% buffer B over 105 min, a second gradient from 28% B to 35% B within 10 min, and a final increase to 90% B in 10 min. Isocratic washing with 90% B was performed for 10 min, then decreased to 5% in 0.1 min and held at 5% for additional 9.9 min for re-equilibration. The Orbitrap was operated in a cycle time (3 s) data dependent mode. An AGC target of 2e5, a maximum injection time of 50 ms, 60% RF lens and a resolution of 120 000 in a scan range of 300-1500 *m/z* in profile mode was used. Monoisotopic precursor selection and dynamic exclusion (60 s) was turned on. For fragmentation, most intense precursors with charges of 2-7 and intensities greater than 5e3 were chosen. Quadrupole isolation was performed using a range of 1.6 *m/z.* Precursor ions were separated using an AGC target of 1e4 and a maximum injection time of 35 ms. Fragmentation was performed using higher-energy collisional dissociation with a collision energy of 30%. Fragments were detected in the ion trap operating at a rapid scan rate.

Data Analysis

Data is acquired using Xcalibur software version 3.1 sp3 and processing of the obtained label-free quantification data is done by mest.[11] In brief, cysteine carbamidomethylation was set as fixed modification and methionine oxidation and *N*-terminal acetylation as variable modifications. Trypsin (without *N*-terminal cleavage to proline) was set as proteolytic enzyme with a maximum of two allowed missed cleavages. Label-free quantification (LFQ) mode was performed with a minimum ratio count of 2. The “match between runs” (0.7 min match and 20 min alignment time window), second peptide identification and iBAQ options were activated. Peptides were searched against the *UniProt*[14] database for *H. pylori* 26695. All other parameters were used as default in the software. LFQ intensities were further processed with Perseus version 2.0.10.0.[13] Peptides of the categories “only identified by site”, “reverse”, or “potential contaminant” were removed and LFQ intensities were log_2 transformed. Rows were annotated in respective groups.

For *H. pylori* Δ*tpx* verification, data was searched for LFQ intensities of O25151 (Uniprot ID, thiol peroxidase, HpTpx) and compared to wildtype *H. plyori* 26695 in a profile plot. Experiment was performed in biological triplicates (n = 3).

1.10 Binding Site Identification isoDTB Analysis

LC-MS/MS Analysis

The samples were analyzed according to a published procedure.[15] Prior to analysis dried peptides were reconstituted in 0.1% (v/v) TFA *via* sonication (3 × 5 min, *Bandelin* Sonorex) and filtered through 0.22 µm PVDF filters (*Millipore*). Samples were transferred to MS vials and analysed via HPLC-MS/MS using an UltiMate 3000 nano HPLC system (*Dionex*) equipped with Acclaim C18 PepMap100 trap column (75 µm ID × 2 cm, Acclaim, *ThermoFisher*) and Aurora Ultimate™ (1st generation, 20 cm nanoflow UHPLC compatible, *ionopticks*) separation columns coupled to a Q Exactive Plus Orbitrap Mass Spectrometer (*Thermo Fisher Scientific*). Samples were loaded onto the trap column and washed with TFA (0.1% in ddH_2O). The subsequent separation was carried out with a flow rate of 400 nL/min using buffer A (0.1% FA in ddH_2O) and buffer B (0.1% FA in MeCN). The separation column was heated to 40 °C. Analysis started with washing for 7 min with 5% buffer B for desalting followed by a gradient from 5% to 40% buffer B over 105 min, a second gradient from 40% B to 60% B within 10 min and a final increase to 90% B in 10 min. Isocratic washing with 90% B was performed for 10

min, then decreased to 5% in 0.1 min and held at 5% for additional 9.9 min for re-equilibration. The Q Exactive Plus mass spectrometer was run in a TOP10 data-dependent mode. In the orbitrap, full MS scans were collected in a scan range of 300-1500 m/z at a resolution of 70 000 and an AGC target of 3e6 with 80 ms maximum injection time. The TOP10 peaks were selected for MS2 scan with a minimum AGC target of 1e3 and isotope exclusion and dynamic exclusion (exclusion duration: 60 s) enabled. Peaks with unassigned charge or a charge of +1 were excluded. Peptide match was "preferred". MS2 spectra were collected at a resolution of 17 500 aiming at an AGC target of 1e5 with a maximum injection time of 100 ms. Isolation was conducted in the quadrupole using a window of 1.6 m/z. Fragments were generated using higher-energy collision induced dissociation (HCD, normalized collision energy: 27%) and finally detected in the orbitrap.

Data analysis

The data analysis described in the following sections was performed according to a published procedure[15] and adjusted for binding site identification studies. The deposited files can be downloaded from the PRIDE database. Detailed results of the analysis can be found in the excel sheet Supporting_Excel_1_isoDTB.

General setup of analysis software

Acquired raw data of performed LC-MS/MS analyses was converted into a mzML format using the MSconvert tool (version: 3.0.21193-ccb3e0136) of the ProteoWizard software (version: 3.0.21193 64bit)[16] using standard settings with vendor´s peak picking enabled. For further data analysis the FragPipe interface (version: 14.0) with MSFragger (version 3.1.1)[17-21], Philosopher (version: 3.3.10)[22], IonQuant (version 1.4.6)[23] and Python (version 3.7.3) was used. A FASTA database for *H. pylori* 26695 was downloaded from www.uniprot.org on 7th of April 2021.[14] The reverse sequences were manually added to the FASTA databases.

OpenSearch Analysis of mass of modifications with FragPipe[17-21 22 23].

To survey the landscape of all mass shifts observed on the peptides of **Metro-P3** labeled *H. pylori* proteome in the data set, an OpenSearch was performed with MSFragger[17-21 22 23]. For this purpose, the following settings were used: Precursor mass tolerance –150 to 1000 Da, (initial) fragment mass tolerance 20 ppm, Calibration and Optimization 'Mass calibration, parameter optimization' enabled, Isotope Error '0', enzyme name 'trypsin' , cut after 'KR', but not before 'P', cleavage 'enzymatic' , missed cleavages '2', Clip N-term N enabled, peptide length 7 to 50, peptide mass range 500 to 5,000 Da, no variable modifications, no fixed modifications, all other options were left at the standard settings. Crystal-C[19] was enabled. PeptideProphet[22] was run with the following setting: '--nonparam --expectscore --decoyprobs --masswidth 1000.0 --clevel -2'. PTMProphet was disabled. ProteinProphet[22] was run with the following settings: '--maxppmdiff 2000000'. Generate report was enabled with the following settings: '--sequential --razor –mapmods --prot 0.01'. Run MS1 quant was disabled. Run TMT-Integrator was disabled. PTM-Shepherd [20] was enabled with the following settings: Smoothing factor '2', Precursor tolerance '0.01 Da', Prominence ratio '0.3', Peak picking width '0.002 Da', Localization background '4'. Annotation tolerance '0.01 Da', Custom mass shifts: a custom mass shift list was used including only UniMod modifications with less than 400 Da molecular weight as previously published[15], Ion Types for modification with 'b' and 'y' enabled and mass fragment charge '2'. Generate Spectral Library was disabled. For downstream data analysis, the 'global.modsummary.tsv' file was searched for mass shifts >482 Da with differences of 6.0075 ± 0.0010 Da between heavy and light isoDTB tags clicked to **Metro-P3**.

ClosedSearch Analysis for Binding site identification studies with FragPipe

To identify binding sites and quantify specific amino acids a *Closed Search* was performed in MSFragger[17-21] [22] [23]. For this purpose, the following settings were used: Precursor mass tolerance –50 to 50 ppm, fragment mass tolerance 20 ppm, Calibration and Optimization 'none', Isotope Error '0/1/2', enzyme name 'trypsin' , cuter after 'K' , but not before 'P', cleavage 'enzymatic' , missed cleavages '2', Clip N-term N enabled, peptide length 7 to 50, peptide mass range 500 to 5,000 Da, no mass offsets, all other options were kept at default settings. Variable modifications were set to the detected mass shifts 660.3462 and 666.3528 found in the Open Search on cysteines (C) with max. 1 occurrence and additionally a variable modification of 57.02146 Da on cysteines with max. 3 occurrences was added. Crystal-C was disabled. PeptideProphet was run with the following settings: '--decoyprobs --ppm --accmass --nonparam –expectscore'. PTMProphet was disabled. ProteinProphet was run with the following settings: '--maxppmdiff 2000000'. Generate report was enabled with the following settings: '--sequential – razor -- prot 0.01'. PTM-Shepherd was disabled. Run MS1 quant was enabled with the following settings: IonQuant enabled, Labeling based quant with the detected mass shifts 660.3462 (light) and 666.3528 (heavy) on cysteines, Re-quantify enabled, Top N ions '3', Min freq. '0.5', Min scans '1', Min isotopes '2', Normalize disabled. RT window (minutes) '0.4' and m/z Window (ppm) '10'.

Generate Spectral Library was disabled. For duplicates, both runs were analyzed as different experiments. For downstream data analysis, the 'ion_label_quant.tsv' files of the two experiments were analyzed separately. For each entry, the 'Modified peptide' was generated as either the 'Light Modified Peptid' or the 'Heavy Modified Peptide' based on the entry with the higher 'PeptideProphet Propability'. The masses of probe modification in the 'Modified Peptide' were replaced by a '*' and the mass of carbamidomethylation ([57.0215]) was deleted if present. The full protein sequence was linked to the table. Based on this information, all peptide sequences that do not occur exactly once in the same protein were excluded and the residue number of the modified residue was determined. The 'Identifier' was generated in the format 'UniProtCode'_C_'residue number', where C represents the one letter code of the modified amino acid cysteine. For each 'identifier', the averaged 'Log2 Ratio HL', which is the log2 transformed ratio of heavy and light ions, was determined as average of the 'Log2 Ratio HL' of all corresponding ions weighted with the 'Total intensity' of the ion, which was calculated as the sum of 'Light Intensity' and 'Heavy Intensity' for each ion. The value was disregarded if the standard deviation of the 'Log2 Ratio HL' values was > 1.41 for all ions of the same 'Identifier'.

Additionally, for each 'Identifier' the 'Total Intensity', 'Total Light Intensity' and 'Total Heavy Intensity' were calculated as the sum of all 'Total Intensity', 'Light Intensity' and 'Heavy Intensity' values of the individual ions, respectively. If several different 'Modified peptides' were detected for the same 'Identifier', the 'Modified Peptide' and the 'Peptide Sequence' with the shortest sequence were kept. For all identifiers, the data for both replicates were now combined in one table (**Table S13**). The 'Log2Ratio HL' values for the replicates were named 'Log2 ratio HL replicate 1' and 'Log2 ratio HL replicate 2'. The average of these two values was calculated and named 'Log2 ratio HL'. The value was disregarded, if the standard deviation between the replicates was > 1.41 or if the identifier was only quantified in one of the replicates 'missed filter'. Remaining peptide sequences that fulfilled all mentioned criteria were assigned to be the binding sites of **Metro-P3** in *H. pylori* 26695 (**Table S13**).

Table S 13: Final binding site identification results after combining data of both replicates of **Metro-P3** labeled *H. pylori* 26695 for the detected mass shifts 660.3462 (light) and 666.3528(heavy) on cysteines.

Protein Name	Identifier	Modified Peptide	Log2 Ratio HL
60 kDa chaperonin	P42383_C63	EIELSC*PVANMGAQLVK	-0.646878149
Thiol peroxidase	O25151_C60	FQVVSALPSLTGSVC*LLQAK	-0.403514043
Cinnamyl-alcohol dehydrogenase ELI3-2	O25732_C90	VGDVVGVGC*FVNSCK	Missed Filter
Thiol peroxidase	O25151_C94	LPSVSFSVISMDLPFSQGQIC*GAEGIK	Missed Filter
Alkyl hydroperoxide reductase C	P21762_C49	DFTFVC*PTEIIAFDKR	Missed Filter
Fumarate hydratase class II	O25883_C85	GELC*GEFPLAIWQTGSGTQTNMNLNEVIANK	Missed Filter
Nitrogen fixation protein NifU	O25009_C54	LIVADYGAEAC*GDAVR	Missed Filter

1.11 Intracellular EC_{50} Determination in *H. pylori*

To determine intracellular EC_{50} values in *H. pylori* 26695, Metro or **MF-03** were added in excess in a competition experiment concentration-dependently to read-out the residual labeling of HpTpx and HpGroEL protein bands with probe **Metro-P3** (1 µM final concentration). The labeling procedure was followed as described above in 'Gel-based Fluorescent Labeling – in situ ABPP'. After SDS-PAGE and fluorescence visualization, the intensity values of the fluorescent bands of the proteins HpTpx (ca. 18 kDa, verified by Δ*tpx* mutant) and HpGroEL (60 kDa band) were determined by *ImageJ*[1] as reported previously.[24] A rectangle was drawn around the bands of interest to plot their profile of intensity by using the 'gel analyser tool'.

Signal intensities were quantified as peak areas and were normalized relative to treatment with probe **Metro-P3** only (100 % labeling) and DMSO treatment (0 % labeling). Respective values were plotted in GraphPad Prism 10 against log-transformed compound concentrations. Intracellular EC_{50} values were calculated from three independent biological replicates (n = 3) by fitting the obtained mean values using the function 'log(inhibitor) vs. response - Variable slope (four parameters)' for HpGroEL. For HpTpx, fitted function was extrapolated at 50% reduction of labeling intensity (y-axis) to reveal respective concentration on x-axis since full competition was not observed for **Metro**. Coomassie staining of the gels revealed equal protein amounts and served as loading control.

1.12 Recombinant Protein Overexpression from *E. coli*

Overexpression of unmodified recombinant proteins

Overexpression of unmodified recombinant protein expression of HpTpx, HpTpx C94A and HpGroEL in *E. coli* was performed as follows. LB-media containing 0.1 mg/mL ampicillin (100 mg/mL stock in ethanol/ddH_2O = 1/1) or 0.025 mg/mL kanamycin (25 mg/mL stock in ddH_2O) was inoculated with 1:100 overnight culture *E. coli* BL21 (DE3) strain containing the expression plasmids pET55-dest-HpTpx/HpTpx-C60A or pEt28a(+)-HpGroEL, respectively. Bacterial cultures were grown at 37 °C while shaking (200 rpm) until reaching an OD_{600} of 0.5-0.6 and protein expression is induced by adding 1 mM *iso*-propyl-1-thio-β-galactopyranoside (IPTG, 1 M stock in ddH_2O) and the protein expression was carried out at 18°C (200 rpm, 16 h).The next day, bacteria were harvested (6000 × g, 4°C, 10 min, rotor SLA-3000; Sorvall RC 6+, *Thermo Scientific*) and washed with PBS (30 mL) once prior to cell lysis and protein purification.

Anaerobic Overexpression in the presence of Metro or ***Metro-P3***

This experimental approach aimed to modify the protein during the expression in *E. coli* under anaerobic conditions to ensure reductive activation of **Metro** or **Metro-P3**.[25]

The expression of recombinant proteins modified with **Metro** or **Metro-P3** in *E. coli* was conducted as follows. Bacterial cultures were grown in 600 mL LB media supplemented with 0.1 mg/mL ampicillin (HpTpx) or 0.025 mg/mL kanamycin (HpGroEL) and inoculated with 1:100 overnight culture *E. coli* BL21 (DE3) strain containing either the expression plasmid pET55-dest-HpTpx or pet28a(+)-HpGroEL, respectively. The cultures were allowed to grow at 37°C with shaking (200 rpm) until reaching an OD_{600} of ≈0.6. Metronidazole or **Metro-P3** were added in different concentrations (1:1000; final concentrations 31.3 µM, 62.5 µM, 125 µM, 250 µM). Protein expression was induced by adding 1 mM *iso*-propyl-1-thio-β-galactopyranoside (IPTG, 1 M stock in ddH_2O). Subsequently, the cultures were transferred into 500 mL sterile Schott Flasks, holding a volume of approximately 600 mL. The flasks were tightly sealed to create a pseudo-anaerobic environment, and a single inversion was performed to ensure homogeneity of the culture. Overexpression was performed at 18°C for 3 h (25 rpm). Bacteria were harvested (6000 × g, 4°C, 10 min, SLA-3000; Sorvall RC 6+, *Thermo Scientific*) and washed with PBS (20 mL) once prior to cell lysis and protein purification.

1.13 Purification of recombinantly expressed proteins

A summary of all purified proteins used within this work including expression conditions, applied purification steps, and determined degree of modification via intact-protein mass spectrometry (IP-MS) are summarized in **Table S14** (HpGroEL) and **Table S15** (HpTpx).

Purification of HpGroEL (C-terminally His tagged protein)

Protein purification was adapted from *Mendoza et al.*[26]

Protein purification of recombinant HpGroEl was performed using a HisTrap HP 5 mL column (*GE Healthcare*) for Ni-NTA affinity purification followed by size exclusion chromatography with a Superdex (HiLoad 16/600 Superdex 200 pg, *Cytiva*) or analytical Superose 6 (Increase 10/300 GL with fractionation) column (*GE Healthcare*). All purification conditions and obtained modification degrees are summarized in **Table S14.**

Table S 14: Summary of all expression and purification conditions and respective observed modification degrees of HpGroEL as mean values of three independently prepared IP-MS measurements (n = 3).

**Protein**	**Expression cond.**	**Purification**	**Mod. Degree**
HpGroEL	18 °C, 16 h, 200 rpm, aerobic	His, SEC (Superdex200)	0%
HpGroEL x Metro	31.3 µM Metro, 18°C, 3 h, anaerobic	His, SEC (Superdex200)	22 %
HpGroEL x Metro	62.5 µM Metro, 18°C, 3 h, anaerobic	His, SEC (Superose 6 Increase)	44 %
HpGroEL x Metro	125 µM Metro, 18°C, 3 h, anaerobic	His, SEC (Superose 6 Increase)	61 %
HpGroEL x Metro	250 µM Metro, 18°C, 3 h, anaerobic	His, SEC (Superose 6 Increase)	68 %
HpGroEL x Metro	500 µM Metro, 18°C, 16 h, anaerobic	His, SEC (Superdex200)	100%
HpGroEL x **Metro-P3**	31.3 µM Metro, 18°C, 3 h, anaerobic	His, SEC (Superose 6 Increase)	25 %
HpGroEL x **Metro-P3**	62.5 µM Metro, 18°C, 3 h, anaerobic	His, SEC (Superose 6 Increase)	34 %
HpGroEL x **Metro-P3**	125 µM Metro, 18°C, 3 h, anaerobic	His, SEC (Superose 6 Increase)	39 %
HpGroEL x **Metro-P3**	250 µM Metro, 18°C, 3 h, anaerobic	His, SEC (Superose 6 Increase)	47 %
HpGroEL x **Metro-P3**	100 µM **Metro-P3**, 18°C, 16 h, anaerobic	His, SEC (Superdex200)	71%

Cell pellets were resuspended in 20 – 50 mL His wash buffer buffer (50 mM Tris-HCl, 10 mM imidazole, pH = 8.0) and lysed by sonication (7 min 30 %, 3 min 70 %, 3 cycles; *Bandelin* Sonolus HD 2070). The lysate was clarified by centrifugation (18 000 rpm, 45 min, 4 °C, SLA-3000), the supernatant was filtered through a 0.45 µm PVDF filter (Whatman GD/X25, *Cytiva*) and loaded (via sample pump (S9, Cytiva)) onto a pre-equilibrated HisTrap HP 5 mL column (*Cytiva;* His wash buffer; 50 mM Tris-HCl, 10 mM imidazole, pH = 8.0) integrated into a Äkta purifier10 FPLC system (*GE Healthcare*). After loading, non-specifically bound proteins were removed by extensive washing with 1% buffer B (His Elution Buffer; 50 mM Tris-HCl, 500 mM imidazole, pH = 8.0) for 5 column volumes (CVs). Then, His tagged HpGroEL was eluted with a linear gradient from 1% to 100% buffer B over 7.5 CVs followed by isocratic elution at 100% B

for 2.5 CVs. The protein containing elution fractions were concentrated (*Amicon*, 30 kDa cut-off, 3500 × g, 4°C) and preparative size-exclusion chromatography was performed over 1.25 column volumes with the concentrated sample using either a 120 mL pre-equilibrated Superdex column (HiLoad 16/600 Superdex 200 pg, *Cytiva*) or a 24 mL Superose 6 (Increase 10/300 GL) column in SEC buffer (50 mM Tris-HCl, pH = 7.5) on the same purification system. Peaks were analyzed using SDS-Page and IP-MS (see 'Intact Protein MS') to concentrate protein with correct monomeric masses and protein aliquots were snap frozen in liquid nitrogen and stored at -80 °C. Protein concentrations were measured at 280 nm on a *Tecan* Infinite® M Nano plate reader in a NanoQuant plate™.

Due to the tedious purification leading to very low yields, anaerobic expressions of HpGroEL with Metronidazole or **Metro-P3** in different concentrations (final concentrations 31.3 µM, 62.5 µM, 125 µM, 250 µM) were only subjected to IP-MS and could not be used for ATPase activity assays. Only purifications of unmodified HpGroEL and the modification degrees 100 % Metro and 71% **Metro-P3** led to sufficient protein amounts that were further projected to biological activity assays.

Purification of unmodified and modified HpTpx and HpTpx C94A mutant (Strep II tagged proteins)

Protein purification of recombinant HpTpx was performed using a StrepTap 5 mL column (*GE Healthcare*) for affinity purification followed by size exclusion chromatography with a Superdex (HiLoad 16/600 Superdex 75 pg, *Cytiva*) if indicated. All purification conditions and obtained modification degrees are summarized in **Table S15.**

HpTpx purification was adapted Nguyen et al.[27]

Table S 15: Summary of all expression and purification conditions and respective observed modification degrees of HpTpx as mean values of three independently prepared IP-MS measurements (n = 3). Strep = Strep affinity purification, His = His affinity purification, SEC = size exclusion chromatography.

**Protein**	**Expression cond.**	**Purification**	**Mod. Degree**	
HpTpx	18 °C, 16 h, 200 rpm, aerobic	Strep	0 %	
HpTpx x Metro	31.3 µM Metro, 18°C, 3 h, anaerobic	Strep	8 %	
HpTpx x Metro	62.5 µM Metro, 18°C, 3 h, anaerobic	Strep	12 %	
HpTpx x Metro	125 µM Metro, 18°C, 3 h, anaerobic	Strep	24 %	
HpTpx x Metro	250 µM Metro, 18°C, 3 h, anaerobic	Strep	42 %	
HpTpx x Metro	1 mM Metro, 18°C, 16 h, anaerobic	Strep	95 %	
HpTpx x **Metro-P3**	31.3 µM **Metro-P3**, 18°C, 3 h, anaerobic	Strep	34 %	
HpTpx x **Metro-P3**	62.5 µM **Metro-P3**, 18°C, 3 h, anaerobic	Strep	49 %	
HpTpx x **Metro-P3**	125 µM **Metro-P3**, 18°C, 3 h, anaerobic	Strep	71 %	
HpTpx x **Metro-P3**	250 µM **Metro-P3**, 18°C, 3 h, anaerobic	Strep	88 %	
HpTpx x **Metro-P3**	100 µM **Metro-P3**, 18°C, 16 h, anaerobic	Strep	98 %	
HpTpx	18 °C, 16 h, 200 rpm, aerobic	Strep, SEC (Superdex75)	0%	
HpTpx C94A x Metro	500 µM, 18°C, 16 h, anaerobic	Strep, SEC (Superdex75)	≈100%	Crystallography
HpTpx C94A x **Metro-P3**	100 µM **Metro-P3**, 18°C, 16 h, anaerobic	Strep, SEC (Superdex75)	≈100%	

Cell pellets were resuspended in 20 – 50 mL lysis buffer (50 mM NaH_2PO_4, 300 mM NaCl, 1 mM PMSF, pH = 8.0) and lysed by sonication (7 min 30 %, 3 min 70 %, 3 cycles; *Bandelin* Sonolus HD 2070). The lysate was clarified by centrifugation (18 000 rpm, 45 min, 4 °C, SLA-3000, Sorvall RC 6+, *Thermo Scientific*), the supernatant was filtered through a 0.45 µm PVDF filter (Whatman GD/X25, Cytiva) and loaded via sample pump (S9, Cytiva) onto an pre-equilibrated (with Strep binding buffer; 50 mM NaH_2PO_4, 300 mM NaCl, pH = 8.0) StrepTrap column (*GE Healthcare*) integrated into an ÄKTA pure 25 FPLC protein purification system (*GE Healthcare*, software: unicorn 7.5) coupled to a fraction collector (F9-C, *GE Healthcare*). The column was extensively washed with Strep binding buffer for 5 column volumes (CVs), and the protein was eluted with Strep elution buffer (50 mM NaH_2PO_4, 300 mM NaCl, 2.5 mM desthiobiotin (DTB), pH = 8.0) for 5 CVs. Fractions containing protein based on UV-detection

were pooled and concentrated with centrifugal mass filters (*Amicon*, 10 kDa cut-off, 3500 × g, 4°C) while the buffer was exchanged to HEPES buffer (20 mM, pH = 7). Protein concentrations were measured at 280 nm on a *Tecan* Infinite® M Nano plate reader in a NanoQuant plate™ and 50 µL aliquots were snap frozen in liquid nitrogen and stored at –80 °C. Correct protein mass or modification degree of protein was checked via Intact-Protein Mass Spectrometry (IP-MS). The purity of HpTpx was verified by SDS-PAGE and used in activity assays without further purification.

For crystallography, HpTpx or HpTpx C94A modified with **Metro** or **Metro-P3** were further purified using size-exclusion chromatography. Preparative size-exclusion chromatography was performed over 1.25 column volumes with the concentrated sample using a 120 mL pre-equilibrated Superdex column (HiLoad 16/600 Superdex 75 pg, *Cytiva*) in SEC buffer (20 mM HEPES, pH = 7.0) on the same purification system. Monomeric protein fractions were pooled, concentrated and protein concentrations were measured at 280 nm on a *Tecan* Infinite® M Nano plate reader in a NanoQuant plate™. Monomeric protein fractions of HpTpx or HpTpx C94A modified with Metro or **Metro-P3** were pooled, and correct mass and degree of modification was determined by IP-MS (see 'Intact Protein MS'). Proteins were concentrated to final concentrations of 20 – 30 mg/mL measured at 280 nm on a *Tecan* Infinite® M Nano plate reader in a NanoQuant plate™ and directly subjected to crystallographic analysis.

1.14 Intact Protein MS Measurement and Data Analysis

IP-MS measurements were carried out on an Ultimate 3000 RSLC system (*Thermo Scientific*) coupled to either an LTQ Orbitrap XL, LTQ FT Ultra or Q Exactive Plus mass spectrometer (*Thermo Scientific*). For all devices, protein desalting was carried out using a MassPREP desalting column (*Waters*) at 22 °C. Gradient elution was carried out with buffer A (0.1% formic acid in ddH2O; LC-MS grade, *Fisher Analytics*) and buffer B (0.1 % formic acid in acetonitrile; LC-MS grade, *Fisher Analytics*). After 2 min pre-equilibration with 6 % B, protein samples were injected and eluted with a linear gradient from 6 to 95% B over 1.5 min followed by isocratic elution for 2 min at 95% B at a 300 µL/min flow rate. The column was re-equilibrated with 6 % B for 1 min.

LTQ Orbitrap XL mass spectrometric measurements were conducted in HESI-positive mode (H-ESI-II source, *Thermo Scientific*) with the following parameters: 4.0 kV spray voltage, 350 °C capillary temperature, 31 V capillary voltage, 110 V tube lens, 30 L/h sheath gas, 15 L/h aux gas. Full scan measurements were accomplished in a range from 300 to 2000 *m*/*z* in profile mode in the orbitrap at a resolution of 100 000.

LTQ FT mass spectrometric measurements were conducted in HESI-positive mode (H-ESI-II source, *Thermo Scientific*) with the following parameters: 4.0 kV spray voltage, 275 °C capillary temperature, 31 V capillary voltage, 110 V tube lens, 60 L/h sheath gas, 10 L/h aux gas, sweep gas 0.2 L/h. Full scan measurements were performed in a range from 600 to 2000 *m*/*z* in profile mode at a resolution of 200 000.

Q Exactive Plus mass spectrometric measurements were conducted in HESI-positive mode (H-ESI-II source, *Thermo Scientific*) with the following parameters: 3.5 kV spray voltage, 350 °C capillary temperature, S-lens RF level 60.0, 40 L/h sheath gas, 10 L/h aux gas. Full scan measurements were performed in a range from 300 to 2000 *m*/*z* in profile mode in the orbitrap at a resolution of 17 500. With a maximum injection time of 200 ms and an AGC target of 1e6.

Raw spectra were processed with *UniDec* 2.6.7 or *ProMass HR for Xcalibur* (version 4.0) for deconvolution and deconvoluted masses plotted against normalized intensity by GraphPad Prism 10. Respective protein masses of HpGroEL and HpTpx with or without modification with **Metro** or **Metro-P3** are summarized in **Table S16**.

Table S 16: Found protein masses (±2 Da) after deconvolution of IP-MS spectra of HpGroEL and HpTpx unmodified or modified with **Metro** or **Metro-P3**. Met = methionine, Metro-NH_2 = amine form of **Metro**, Metro-P3-NH_2 = amine form of **Metro-P3**.

Protein	Found mass [Da]	Found adduct
HpGroEL (unmodified)	60 049	[HpGroEL – Met]
HpGroEL x **Metro**	60 189	[HpGroEL + **Metro**-NH_2 – Met]
HpGroEL x **Metro-P3**	60 213	[HpGroEL + **Metro-P3**-NH_2 – Met]
HpTpx (unmodified)	20 581	[HpTpx – Met]
HpTpx x **Metro** (mono-modified)	20721	[HpTpx + **Metro**-NH_2 – Met]
HpTpx x **Metro** (di-modified)	20859	[HpTpx + 2 × **Metro**-NH_2 – Met]
HpTpx x **Metro-P3** (mono-modified)	20 746	[HpTpx + **Metro-P3**-NH_2 – Met]
HpTpx x **Metro-P3** (di-modified)	20 908	[HpTpx + 2 × **Metro-P3**-NH_2 – Met]

1.15 Recombinant Protein Labeling of HpTpx from *E. coli*

For recombinant labeling of HpTpx after purification from *E. coli*, HpTpx was diluted to a final concentration of 1 µM in PBS. 50 µL aliquots were prepared for different concentrations of **Metro-P1**, **Metro-P2** and **Metro-P3** as well as a DMSO and heat control. Heat control samples are diluted in PBS to a final concentration of 1 µM and heat shock was performed at 95°C for 20 min. To each protein sample, probes were added to final concentrations of 100 µM, 10 µM and 1 µM and labeled for 1 h (37 °C, 200 rpm). Addition of 1 % (v/v) DMSO was used as control.

For comparison of unmodified HpTpx with HpTpx modified with **Metro-P3** (98%) after anaerobic overexpression, proteins were diluted to 1 µM and 100 µM **Metro-P3** or 1 % (v/v) DMSO were added to the proteins and incubated for 1 h (37 °C, 200 rpm).

After labeling, *Click*-chemistry is performed by addition of 1 µL rhodamine azide (5 mM in DMSO), 1 µL tris(2-carboxyethyl)phosphine (TCEP, 52 mM in ddH2O), 3 µL 1× tris(benzyltriazoyl-methyl)amine (TBTA, 1.67 mM in 80% *t*BuOH and 20% DMSO) and 1 µL $CuSO_4$ (50 mM in ddH_2O) per 50 µL sample. The reaction was stopped by adding 50 µL loading buffer (2×) and the samples were analysed by SDS-PAGE as described before.

1.16 Analytical Protein Expression and *in situ* Labeling of HpTpx or Mutants in *E. coli*

Dose-and time-dependent labeling of HpTpx with **Metro-P3** during protein overexpression in *E. coli* was performed as described in ‘Anaerobic Overexpression in the presence of Metro or

Metro-P3' with slight modifications for fluorescent visualization. After IPTG induction, 1.7 mL of bacterial culture (OD_{600} = 0.6) was pipetted into 1.5 mL tubes containing different concentrations of **Metro-P3** (final: 10 µM, 50 µM, 100 µM, 250 µM) and were incubated at 18 °C for different periods of time (2 h, 3 h, 4 h).

For labeling of HpTpx and mutants (C60A, C94A and C60A-C94A), 1.7 mL of each bacterial culture (OD_{600} = 0.6) were pipetted into 1.5 mL tubes containing **Metro-P3** (final: 100 µM) and incubated at 18 °C for 2 h. Labeling of HpTpx and mutants *in situ* during overexpression was performed in biological triplicates.

After incubation time, cell pellets were centrifuged (6000 × g, 10 min, 4 °C) and washed with PBS (0.5 mL) once. Cell pellets were resuspended in 200 µL PBS + 0.4 % SDS and lysed by sonication. (3 × 20 s, 30 %, pulse 5; Bandelin Sonolus HD 2070). Samples were centrifuged (21 000 × g, 30 min, 4 °C) and 100 µL of supernatant were transferred to new tube. Click-chemistry was performed by addition of 1 µL rhodamine azide (10 mM in DMSO), 2 µL tris(2-carboxyethyl)phosphine (TCEP, 52 mM in ddH2O), 6 µL 1× tris(benzyltriazoyl-methyl)amine (TBTA, 1.67 mM in 80% tBuOH and 20% DMSO) and 2 µL CuSO4 (50 mM in ddH2O) per 100 µL sample. The reaction was stopped by adding 100 µL loading buffer (2×) and the samples were analysed by SDS-PAGE as described in section 'SDS-PAGE'.

1.17 Crystallography

Specific conditions for crystal growth were:

- $HpTpx^{red}$: 0.1 M HEPES pH 7.5, 30% PEG 400
- HpTpx_C_RA:**Metro***: 0.01 M $CoCl_2$, 0.1 M TRIS pH 8.5, 20% PVP K15
- HpTpx_C_RA:**Metro-P3***: 0.1 M BISTRIS pH 6.5, 28% PEG monomethylether 2000

Crystals were cryoprotected using a 7:3 mixture of mother liquor and 100% 2,3-butanediol, prior vitrification in liquid nitrogen.

Data Collection and Structural Analysis: High-resolution data sets were obtained using synchrotron radiation:

- $HpTpx^{red}$ and HpTpx_C_RA:**Metro*** crystals at beamline X06SA, Swiss Light Source (SLS), Paul Scherrer Institute, Switzerland.
- HpTpx_C_RA:**Metro-P3*** crystals at beamline ID30B, European Synchrotron Radiation Facility (ESRF), Grenoble, France.

Reflection intensities were evaluated with XDS, and data reductions were performed using XSCALE[28]. Initial phases were determined by Patterson search calculations with PHASER[29], utilizing coordinates predicted by Alphafold2[30]. Model building was conducted with COOT[31], and water molecules were placed automatically using ARP/wARP[32]. Refinements, including restrained and TLS (Translation/Libration/Screw) adjustments, were executed with REFMAC[33], achieving excellent R_{work}, R_{free}, and rmsd for bond angles and lengths (**Table S17**).

Deposition of Structures: Crystal structures have been deposited in the RCSB Protein Data Bank (**Table S17**).

Table S 17: Crystallographic data collection and refinement statistics.

	HpTpx^{red}	HpTpx_C_RA:Metro*	HpTpx_C_RA:Metro-P3*
**Crystal parameters**			
Space group	$P2_1$	C2	$P2_1$
Cell constants [Å/°]	a = 40.5<br>b = 68.6<br>c= 64.4<br>β= 99.0	a = 67.4<br>b = 71.5<br>c = 40.8<br>β = 115.9	a = 40.6<br>b = 67.8<br>c= 64.0<br>β= 98.7
Subunits / AU[a]	2	1	2
**Data collection**			
Beam line	X06SA, SLS	X06SA, SLS	ID30B, ESRF
Wavelength (Å)	1.0	1.0	0.873
Resolution range (Å)[b]	30-1.75 (1.85-1.75)	30-1.75 (1.85-1.75)	30-1.95 (2.05-1.95)
No. observations	105,494	47,462	70,575
No. unique reflections[c]	34,071	16,754	24,146
Completeness (%)[b]	96.6 (96.5)	95.0 (97.4)	96.2 (96.7)
R_{merge} (%)[b, d]	4.4 (68.5)	3.4 (59.2)	4.3 (63.8)
I/□ (I)[b]	14.1 (2.2)	14.3 (3.1)	11.8 (2.1)
**Refinement**			
Resolution range (Å)	30-1.75	30-1.75	30-1.95
No. refl. working set	32,361	15,911	22.932
No. refl. test set	1,703	837	1,207
No. non hydrogen	2,629	1,333	2,588
No. of ligand atoms	-	10	12
Solvent	79	44	18
R_{work}/R_{free} (%)[e]	18.3 / 21.9	19.7 / 22.4	17.7 / 21.7
r.m.s.d. bond (Å)/angle (°)[f]	0.003 / 1.2	0.006 / 1.2	0.004 / 1.1
Average B-factor (Å^2)	36.0	56.7	55.3
Ramachandran Plot (%)[g]	99.1 / 0.9 / 0	98.8 / 1.2 / 0	99.4 / 0.6 / 0
PDB accession code	9F5V	9F64	9F65

[a] Asymmetric unit

[b] The values in parentheses for resolution range, completeness, R_{merge} and I/σ (I) correspond to the highest resolution shell

[c] Data reduction was carried out with XDS and from a single crystal. Friedel pairs were treated as identical reflections

[d] $R_{merge}(I) = \Sigma_{hkl}\Sigma_j \mid I(hkl)_j - \langle I(hkl) \rangle \mid / \Sigma_{hkl} \Sigma_j I(hkl)_j$, where $I(hkl)_j$ is the jth measurement of the intensity of reflection hkl and <I(hkl)> is the average intensity

[e] $R = \Sigma_{hkl} \mid |F_{obs}| - |F_{calc}| \mid / \Sigma_{hkl} |Fobs|$, where R_{free} is calculated without a sigma cut off for a randomly chosen 5% of reflections, which were not used for structure refinement, and R_{work} is calculated for the remaining reflections

[f] Deviations from ideal bond lengths/angles

[g] Percentage of residues in favored region / allowed region / outlier region

1.18 Minimal Inhibitory Concentration Assay (MIC Assay)

The minimum inhibitory concentration (MIC) represents the lowest concentration of an antibiotic that inhibits visible growth of a microorganism after a distinct incubation time and was performed as a 96 well plate-based MIC assay (transparent Nunc 96-well flat bottom, *Thermo Fisher)*.

For MIC assays in *C. difficile*, *E. coli* and *S. aureus,* overnight cultures are diluted 1:2500 (*C. difficile*) or 1:10 000 (*E. coli* and *S. aureus*) in their corresponding media (*C. difficile*: BHI supplemented with 5 g/L yeast extract, 0.1 % (w/v) L-cysteine and 1 mg/L resazurine; *E. coli* and *S. aureus*: LB medium). 1.5 µL 100 × stock of compound is pipetted into a 96 well plate in triplicates and 148.5 µL bacterial solution is added to obtain desired concentration. The plate is incubated at 37 °C for 24 hours (200 rpm, aerobic conditions, *E. coli* and *S. aureus*) or 72 h in an anaerobic chamber (85 % nitrogen, 5 % H_2, 10 % CO_2, *C. difficile*) before visual readout by eye or Tecan (OD_{600}) revealed respective MICs as the lowest concentration without growth.

For MIC assays in anaerobic bacteria from the murine gut including *Bacteroides caecimuris* (I48), *Muribaculum intestinale* (YL27)*, Enterococcus faecalis (KB1), Bifidobacterium animalis (YL2),* overnight cultures were diluted to reach a final OD_{600} = 0.0015 in AAM medium in an anaerobic** chamber. 50 µL of these bacterial suspensions were added to a sterile 96-well microtiter plate containing two-fold serial dilutions of respective compound in AAM media (50 µL). Dilution series of each compound was analyzed for microbial growth after incubation for 24 hours (For I48, KB1 and YL2) and for 48 h (YL27) either by eye or by OD_{600} readout with a Tecan reader. Experiment was performed in biological duplicates.

1.19 DNA-based Antioxidant Activity Assay

A DNA supercoiling assay was performed as described previously with some modifications.[27,34]

Nicking of supercoiled DNA was carried out in a reaction mixture containing 280 ng of pUC19 DNA (70 ng/µL; *Carl-Roth*), 5 mM DTT, 15 µM $FeCl_3$, and 20 µM of unmodified HpTpx or modified HpTpx with **Metro** or **Metro-P3** with different modification degrees in Hepes-NaOH (100 mM, pH 7.0) buffer, and incubated at 37 °C for 30 min. 1 µL of EDTA at a final concentration of 250 mM was added to stop the reaction. A positive control was carried out with the addition of EDTA (final conc. 250 mM) before incubation. HpTpx was omitted from the reaction mixture to serve as a negative control. 4 µL of each sample was loaded on 0.8 % (w/v) agarose gel and run at 100 V for 75 min. DNA rescuing activity was determined as the ratio of supercoiled /open circular form of DNA. Band intensities were quantified by *ImageJ*[1]. Obtained ratios were normalized to positive control ratio (100% activity) and negative EDTA control was subtracted as baseline. Experiment was performed in 12 biologically independent experiments (n = 12). Statistical significance was determined by ordinary one-way ANOVA with multiple comparisons (no correction).

1.20 MTT Assay

MTT assays were performed in flat bottom 96 well plates (Nunclon™ Delta 96-Well MicroWell™ Plates, *Thermo Scientific*). For the assay, 4000 HeLa and 8000 HepG2 cells in 200 µL DMEM medium (+ 10% FCS, 2 mM L-glutamine) per well were seeded and incubated overnight to allow the cells to adhere to the wells. The next day, the medium is removed and 100 µL/well containing 1 µL of 100 × compound stock solution or 1 % (v/v) DMSO as growth control in DMEM medium (+ 10% FCS, 2 mM L-glutamine) was added to the cells and incubated for 24 h

(37 °C, 5 % CO_2) before 20 µL of Thiazolyl Blue Tetrazolium Bromide Solution (MTT reagent, 5 mg/mL in PBS, *Sigma Aldrich*) were added to each well. The cells were incubated for 2 h (37 °C, 5 % CO_2) to allow the MTT reagent to be metabolized. After medium aspiration, the resulting formazan is resuspended in 200 µL DMSO/well. Absorbance as measured at 570 nm and background subtracted at 630 nm by a Tecan Infinite® M200 Pro Reader. Residual metabolic activity of each condition is normalized to the DMSO controls (100 % activity). Curve fitting to determine IC_{50} values was performed by applying a non-linear least-squares fit (log(inhibitor) vs. response curve with variable slope model) with GraphPad Prism 10. The experiment was performed in two cell lines in technical triplicates.

1.21 Plasma Stability Assay

Prior to measurements, mouse serum (*Biowest*) was diluted 1:2 in PBS (10 mM Na_2HPO_4, 1.8 mM KH_2PO_4, 140 mM NaCl and 2.7 mM KCl, pH = 7.4) and pre-incubated for 10 min at 37°C (400 rpm, 10 min, Thermomixer, *Eppendorf*). Compounds and standard (caffeine) at a final concentration of 500 µM were added and incubated at 37°C (400 rpm, 10 min, Thermomixer, *Eppendorf*). 25 µL aliquots were taken at distinct timepoints and pipetted into 100 µL acetone (-80°C) and centrifuged (21 000 × g, 10 min, 4°C). The supernatant was transferred to a new tube and the solvent was removed under reduced pressure in a speed vac (Concentrator Plus, *Eppendorf*). Dried pellets were dissolved in 40 µL acetonitrile and 10 µL H_20 (MS-grade) and filtered through modified nylon centrifugal filters (modified Nylon, 0.45 µm, VWR). For analysis, sample was loaded onto a C18 column (*Accucore*, *Thermo Fisher*) at 22 °C. Gradient elution was carried out with buffer A (0.1% formic acid in ddH_2O; LC-MS grade, *Fisher Analytics*) and buffer B (0.1 % formic acid in acetonitrile; LC-MS grade, *Fisher Analytics*). After 3 min pre-equilibration with 2 % B, protein samples were injected and eluted with a linear gradient from 2 to 98% B over 5.5 min followed by isocratic elution for 1 min at 98% B at a 300 µL/min flow rate. The column was re-equilibrated with 2 % B for 2 min. MS measurements were carried out in positive mode on an Ultimate 3000 RSLC system coupled to a LTQ Orbitrap XL mass spectrometer (*Thermo Scientific*) with ESI ion source (spray voltage: 4 kV, capillary temp.: 275 °C, capillary voltage: 24 V, tube lens: 110 V). Full scan measurements were performed in a range from 100 to 1000 *m*/*z* in centroid mode in the orbitrap at a resolution of 60 000. X.calibur 2.2 Qual Browser was used for calculation of peak areas and quantification. Assay was performed in three biological replicates (n = 3). Per replicate, each data point was normalized to the internal standard caffeine and referenced to t = 0 h (100% stability). Plasma stability was determined by plotting normalized mean stability values of three biological replicates against sampling time in GraphPad Prism 10.

1.22 AGS Adhesion Assay with *H. pylori* 26695

This protocol describes the infection of adherent AGS cells with *H. pylori* 26695 with and without compound addition adapted from literature procedures.[35-37]

Gastric adenocarcinoma cells (AGS) were seeded in 24 well plates (1 × 10^5 cells per well) and cultured for 24 hours to obtain an attached monolayer. Cells from one well were detached using Trypsin-EDTA (0.25 %) and counted. The medium was aspirated, and cells were washed with PBS to remove antibiotic supplemented media to ensure growth of *H. pylori*. DMEM supplemented with 20% BHI and 10% FCS (500 µL) was used for adhesion assay experiment. *H. pylori* liquid cultures were prepared by inoculating BHI + 10 % FCS medium *with H. pylori* 26695 colonies from WC Dent (*H. pylori* selective) agar plates and then incubated for 24 h (37 °C, 100 rpm, microaerophilic). *H. pylori* strain 26695 was added in a multiplicity of infection

(MOI) of 10 to co-culture with AGS. After 2 hours, the medium was aspirated, and the coculture was washed with PBS once to remove non-adherent *H. pylori* cells. DMEM with 10% FCS supplemented with metronidazole or **Metro-P3** in various concentrations (1 µM and 25 µM) was added to each well and incubated for 16 hours at 37°C (5% CO_2). DMSO was used as a growth control. The co-culture was washed with PBS three times to remove compound supplemented media, and ddH_2O was added to lyse the AGS cells. After 45 minutes, 1:2, 1:10, 1:100 or 1:1000 dilutions of lysed AGS cells were spread on WC Dent agar plates. Colony-forming units of *H. pylori* were counted after 5 days of incubation (37°C, microaerophilic conditions). Experiment was performed in three or four biological independent experiments with technical triplicates per experiment.

1.23 HPLC-MS/MS Analysis for ADME and PK Studies

HPLC conditions were as follows: column: Agilent Zorbax Eclipse Plus C18, 50 × 2.1 mm, 1.8 µm; temperature: 30°C; injection volume: 1 µL; flow rate: 700 µL/min; solvent A: water + 0.1 % formic acid; solvent B: acetonitrile + 0.1 % formic acid; gradient for **Metro-P1**, **Metro-P3**, **MF-01**, **MF-02**, **MF-03** and **MF-07**: 99 % A at 0 min, 99 % - 50% A from 0.1 min to 3.5 min, 50 - 0 % A from 3.5 min to 3.8 min, 0 % A until 4.7 min. Mass spectrometric conditions were as follows: Scan type: Q1 and Q3 masses for caffeine, glipizide, metronidazole, **Metro-P1**, **Metro-P2**, **MF-01**, **MF-02**, **MF-03** and **MF-07** can be found in **Table S18** and **Table S19**; peak areas of each sample and of the corresponding internal standard were analyzed using MultiQuant 3.0 software (AB Sciex). Peaks of PK samples were quantified using the calibration curve. The accuracy of the calibration curve was determined using QCs independently prepared on different days. PK parameters were determined using a non-compartmental analysis with PKSolver[38]. HPLC-MS/MS conditions for verapamil, naproxen, procaine, procainamide and propoxycaine (control compounds of the *in vitro* ADME assays) were used as described previously.[39]

Table S 18: Mass spectrometric conditions of analytes for ADME and PK studies.

ID	Q1 Mass [Da]	Q3 Mass [Da]	time [msec]	DP [volts]	CE [volts]	DXP [volts]
**caffeine**	195.024	138.0	20	130	25	14
		110.0	20	130	31	18
**Metro-P1**	209.912	128.0	20	56	17	20
		82.8	20	56	15	12
		43.0	20	56	37	14
**Metro-P3**	195.872	140.0	20	11	15	16
		55.0	20	11	27	6
		41.9	20	11	73	18
**MF-01**	185.900	59.1	20	51	17	8
		128.0	20	51	15	20
		31.1	20	51	35	14
**MF-02**	171.897	140.0	20	26	15	18
		55.0	20	26	23	8
		42.0	20	26	63	10
**MF-03**	199.889	73.0	20	1	17	8
		44.9	20	1	37	10
		128.0	20	1	17	14
**MF-07**	211.894	85.0	20	51	17	24
		41.0	20	51	49	12
		168.1	20	51	15	20
**glipizide**	443.936	170.100	150	-40	-7	-66

Table S 19: Mass spectrometric conditions of analytes using QTrap 7500.

ID	Q1 Mass [Da]	Q3 Mass [Da]	time [msec]	CE [volts]	CXP [volts]
**caffeine**	195.050	100.933	10	12.0	10.0
		116.849	10	10.0	7.0
**metronidazol**	172.062	82.062	10	35.0	4.0
		128.061	10	20.0	3.0
		44.998	10	35.0	17.0
		111.034	10	36.0	17.0
	170.066	109.946	10	-21.0	-7.0
		126.066	10	-11.0	-24.0
		79.972	10	-40.0	-10.0
		140.069	10	-11.0	-8.0
**MF-03**	200.050	45.031	10	29.0	12.0
		73.050	10	19.0	12.0
		128.038	10	18.0	12..0
		103.959	10	15.0	18.0
**Metro-P3**	196.046	140.024	10	16.0	35.0
		55.052	10	65.0	35.0
		42.034	10	65.0	5.0
		110.053	10	22.0	5.0
		163.926	10	10.0	5.0

1.24 *In vivo* Efficacy Studies in Murine Model

Female C57BL/6 wild-type mice (*Envigo*) were housed under specific pathogen-free conditions and were fed *ad libitum*. Six- to eight-week-old mice were infected with *H. pylori* SS1 by oral gavage with $2*10^8$ bacteria resuspended in 200 µL BHI with 20% FCS. Mice were infected twice with *H. pylori* at a time interval of two days. After a stable infection was established (6-8 weeks), mice were treated with different antibiotic triple therapies by oral gavage twice a day for 7 days: metronidazole or **Metro-P3** (14.2 mg/kg/day) or **Metro-P3** (0.02 × 14.2 mg/kg/day = 0.30 mg/kg/day or **MF-01** (0.02 × 14.2 mg/kg/day = 0.30 mg/kg/day) or metronidazole (0.02 × 14.2 mg/kg/day = 0.30 mg/kg/day) with clarithromycin (7.15 mg/kg/day) or clarithromycin only (control group, 7.15 mg/kg/day). One hour before antibiotic treatment, mice were administered proton pump inhibitor omeprazol (400 µmol/kg/day). One group was not treated with antibiotics as a positive colonization control group for CFU determinations. Two weeks after antibiotic treatment, mice were sacrificed by cervical dislocation and colonization was analyzed by plating serial dilutions of stomach tissue. For this, a longitudinal piece of stomach was weighed and homogenized in 1 mL BHI + 20% FCS with a bead mill (Precellys 24, 5000 rpm, 2 × 45 s, 5 s breaks). Serial dilutions (*H. pylori* SS1: $1{:}10^2$, $1{:}10^3$, $1{:}10^4$) in BHI + 20% FCS were prepared and plated on WC-dent blood agar plates supplemented with bacitracin (200 µg/ml), nalidixic acid (10 µg/ml), and polymyxin B (3 µg/ml). After 5 days of culture, CFU's were counted and optionally expanded on WC-dent plates for 2 days and then frozen for later use. All experiments were approved by the Bavarian Government (Regierung von Oberbayern, ROB-55.2-2532.Vet_02-23-90) and conducted in compliance with European guidelines for the care and use of laboratory animals.

In vivo efficacy studies in C57BL/6 mice infected with *H. pylori* SS1 strain and treatment with different regimens: No treatment (n = 7), clarithromycin only (C only, n = 6), triple therapy with low dose of **Metro-P3** (0.02 × **Metro**; n = 4) or **MF-01** (0.02 × **Metro**; n = 5), standard triple therapy with **Metro** (n = 6) or **Metro-P3** (1 × **Metro**, n = 5). Statistical significance was determined by nonparametric Kruskal-Wallis test with multiple comparisons (no correction) to control column (no treatment). The limit of detection (LOD, CFU/mg) was defined as the equivalent of a single colony on the lowest dilution plated and calculated as $LOD = n \times (DF/V_{plate}) \times (V_{hom}/m_{proc})$, where DF is the dilution factor, V_{plate} is the plated volume, V_{horn} the homogenate volume, and m_{proc} the mass of processed tissue.

Uncropped Gels of Supplementary Figures

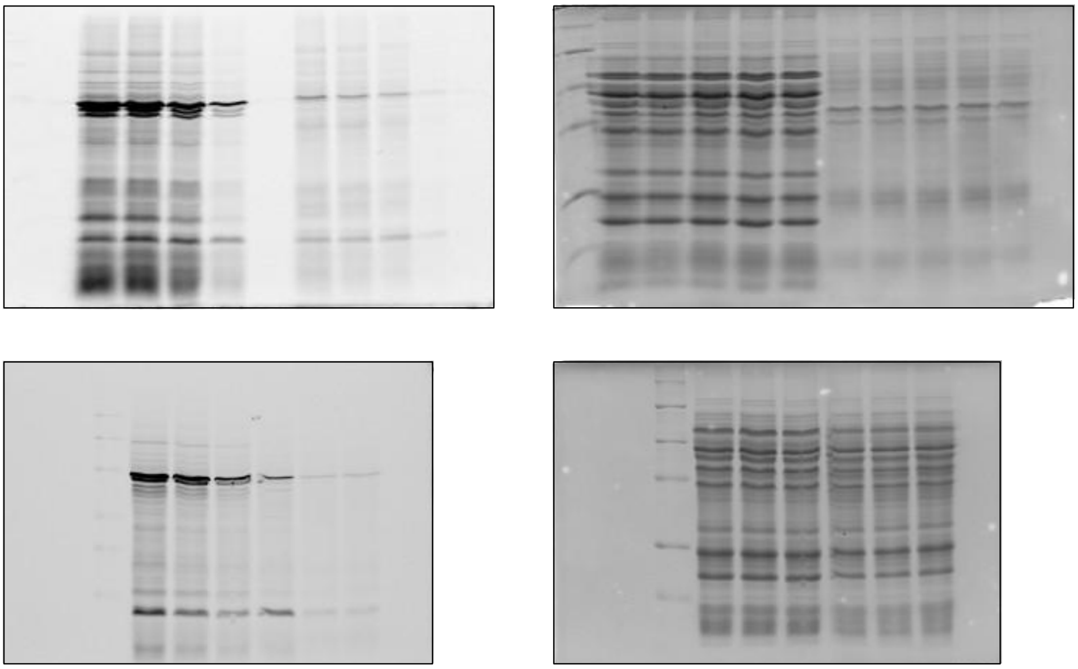

Figure S 27: Uncropped Gels of **Figure S6 A**.

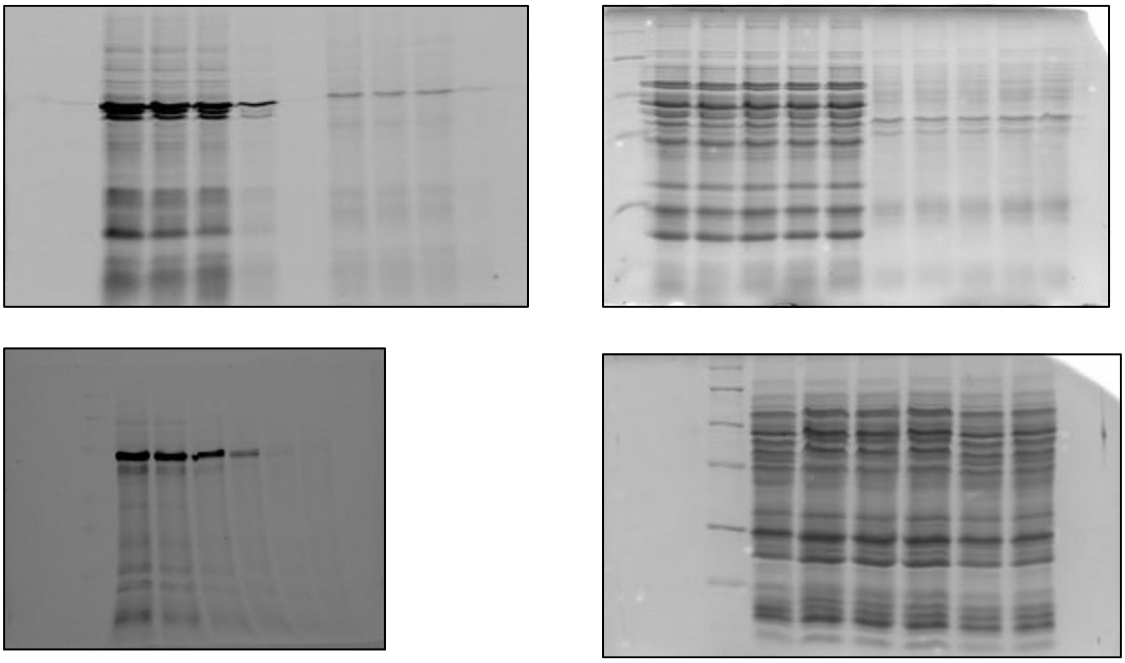

Figure S 28: Uncropped Gels of **Figure S6 B**.

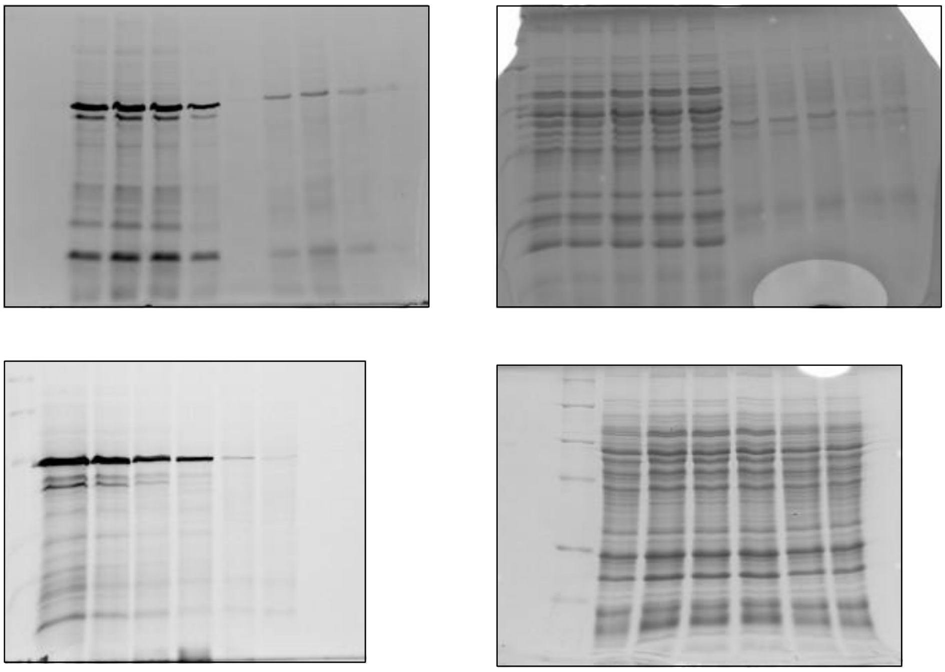

Figure S 29: Uncropped Gels of **Figure S6 C**.

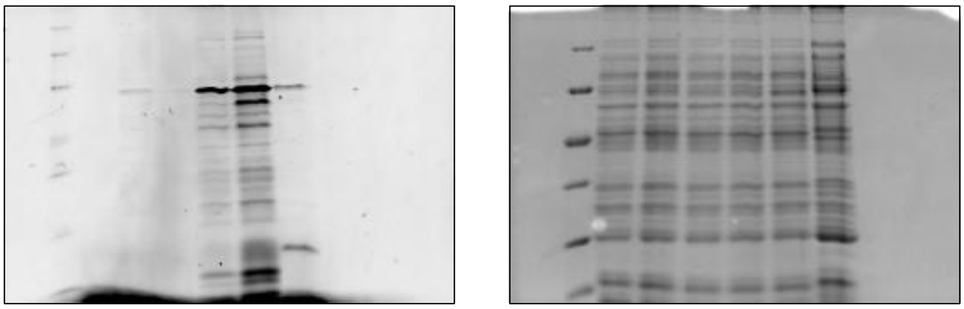

Figure S 30: Uncropped Gels of **Figure S7**.

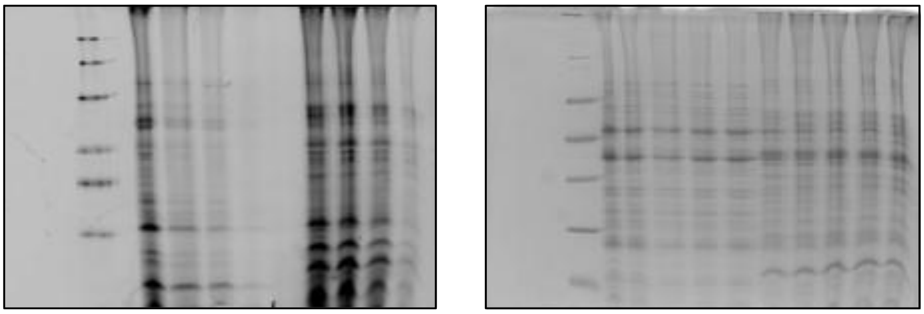

Figure S 31: Uncropped Gels of **Figure S9**.

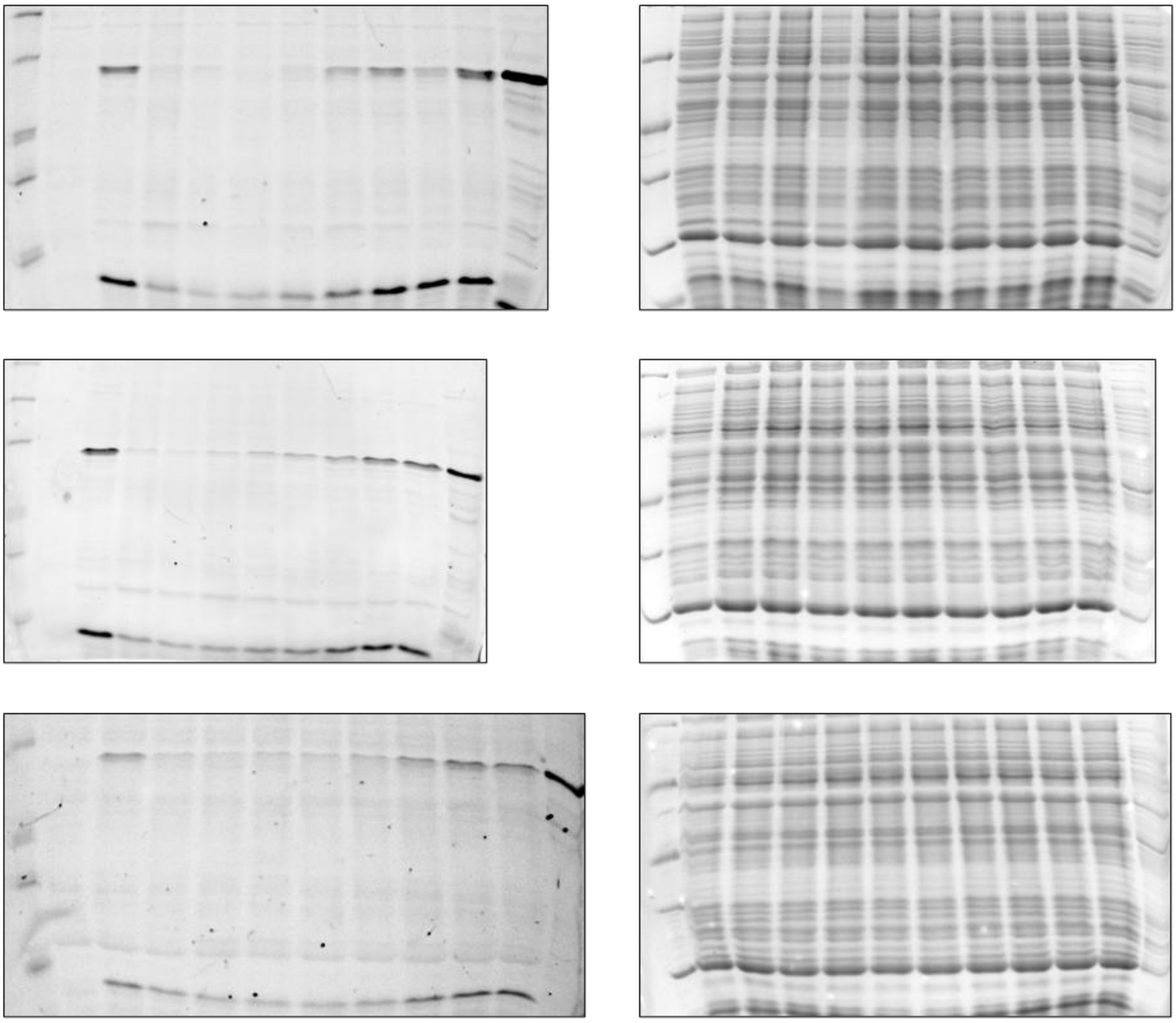

Figure S 32: Uncropped Gels of **Figures S10 A** and **S11 A**.

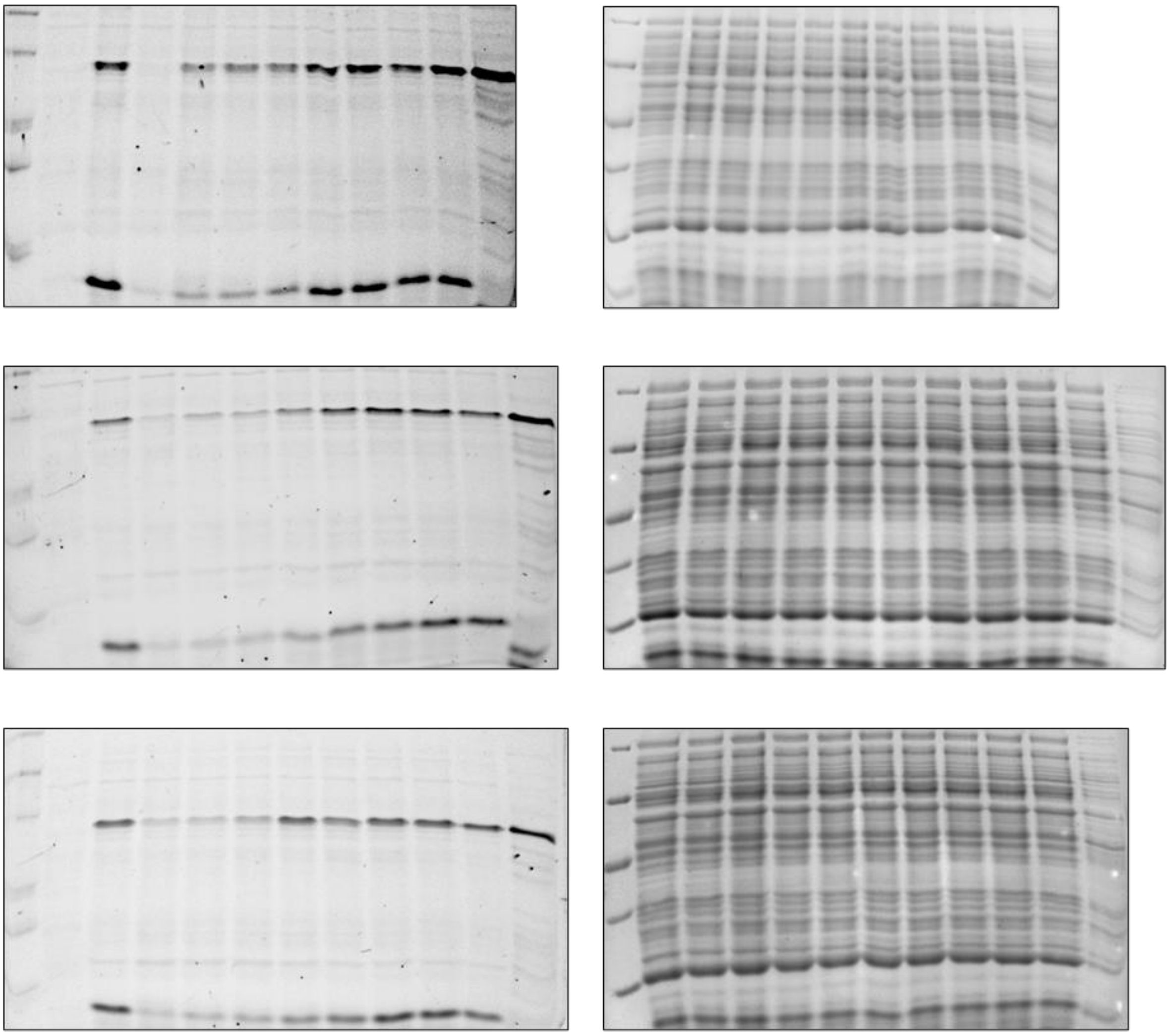

Figure S 33: Uncropped Gels of **Figures S10 B** and **S11 B**.

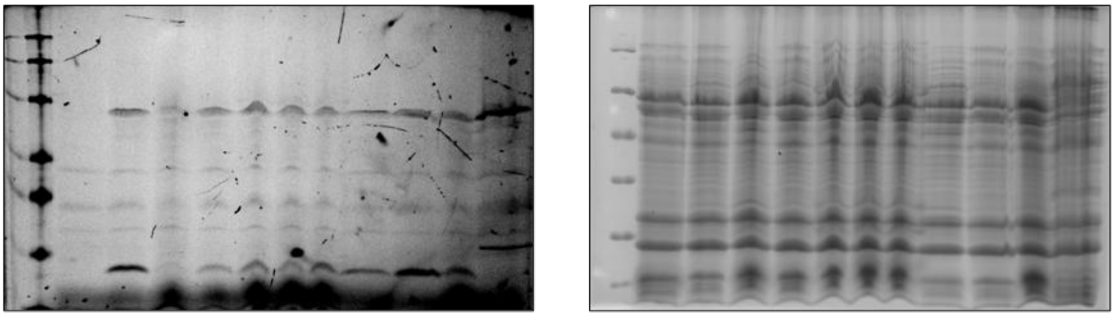

Figure S 34: Uncropped Gels of **Figure S10 C**.

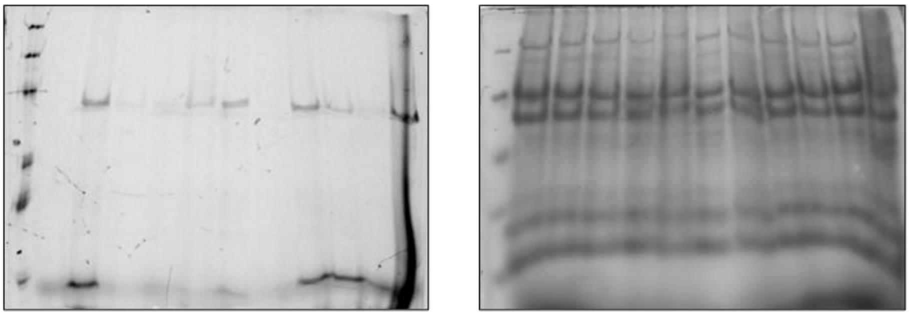

Figure S 35: Uncropped Gels of **Figure S10 D**.

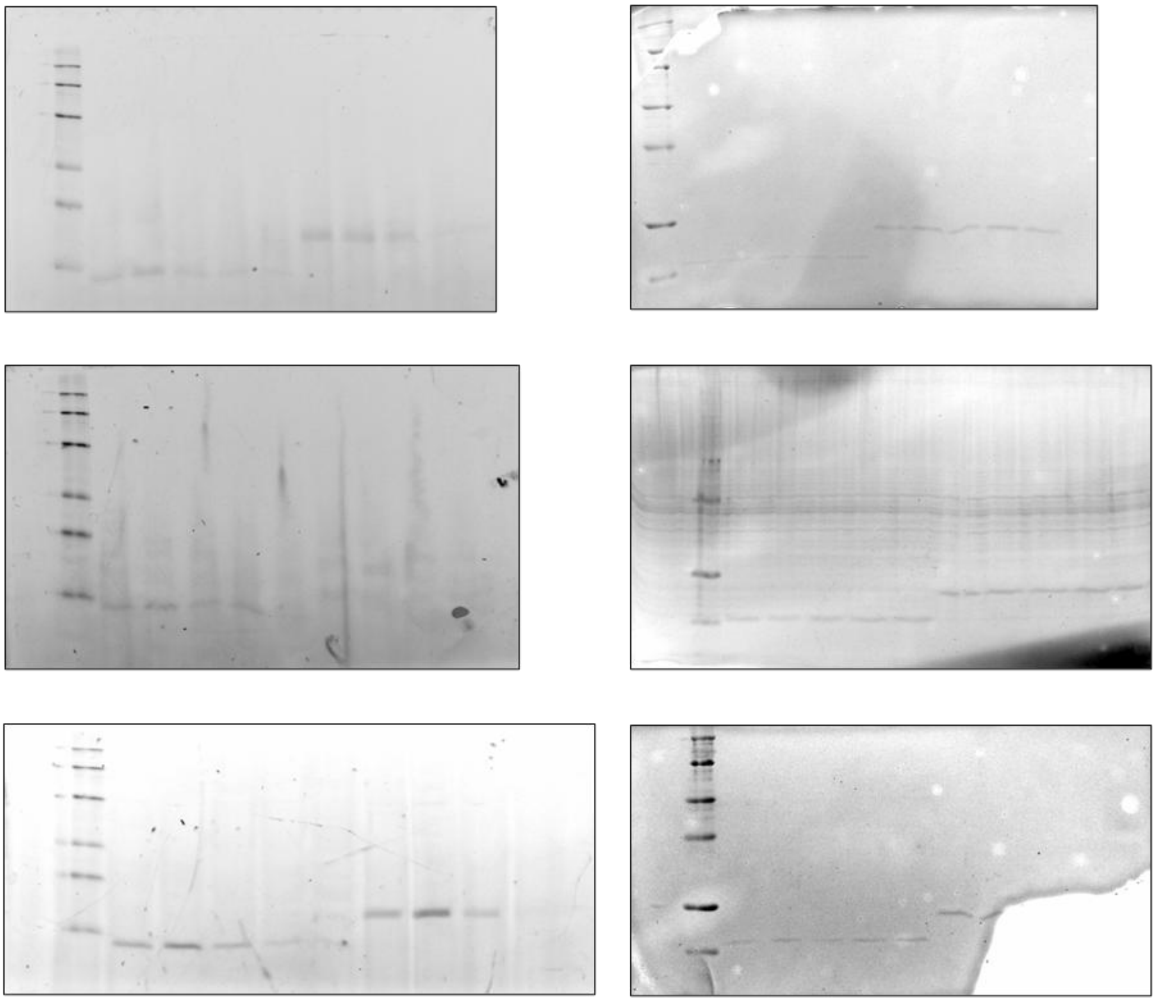

Figure S 36: Uncropped Gels of **Figure S12**.

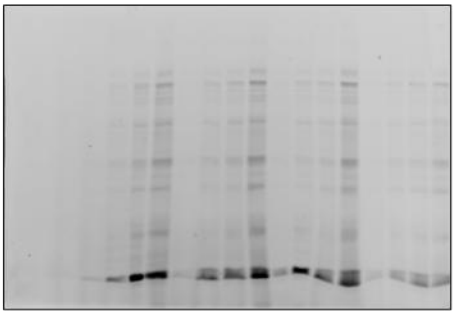 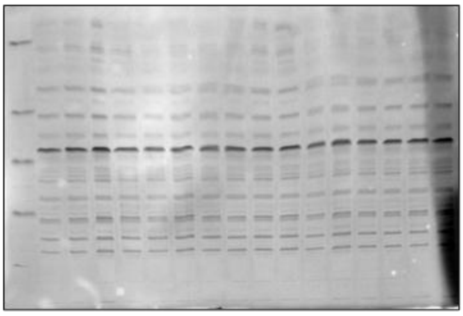

Figure S 37: Uncropped Gels of **Figure S15**.

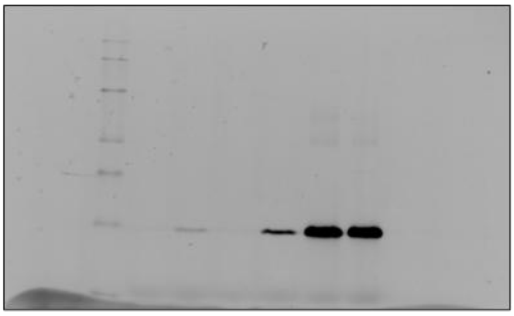 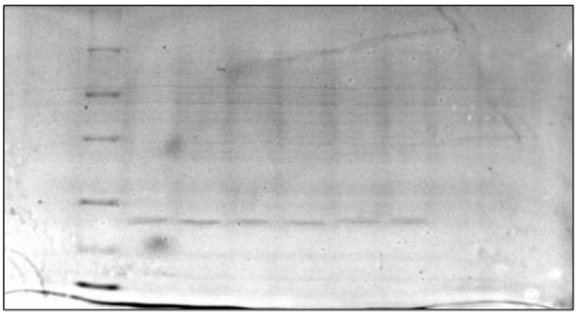

Figure S 38: Uncropped Gels of **Figure S16**.

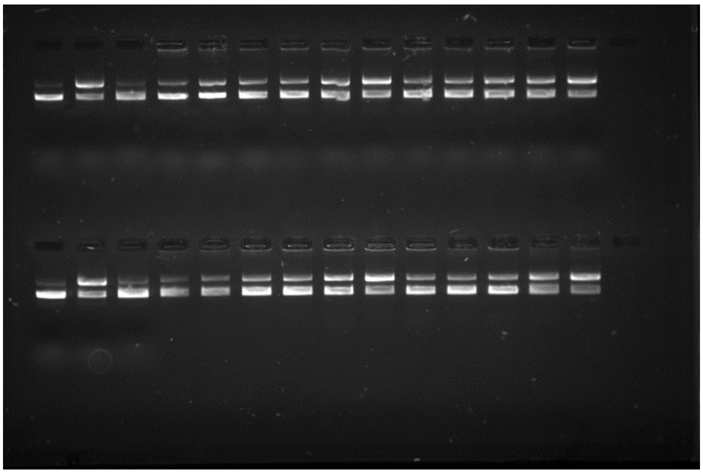

Figure S 39: Uncropped Gel of **Figure S17**.

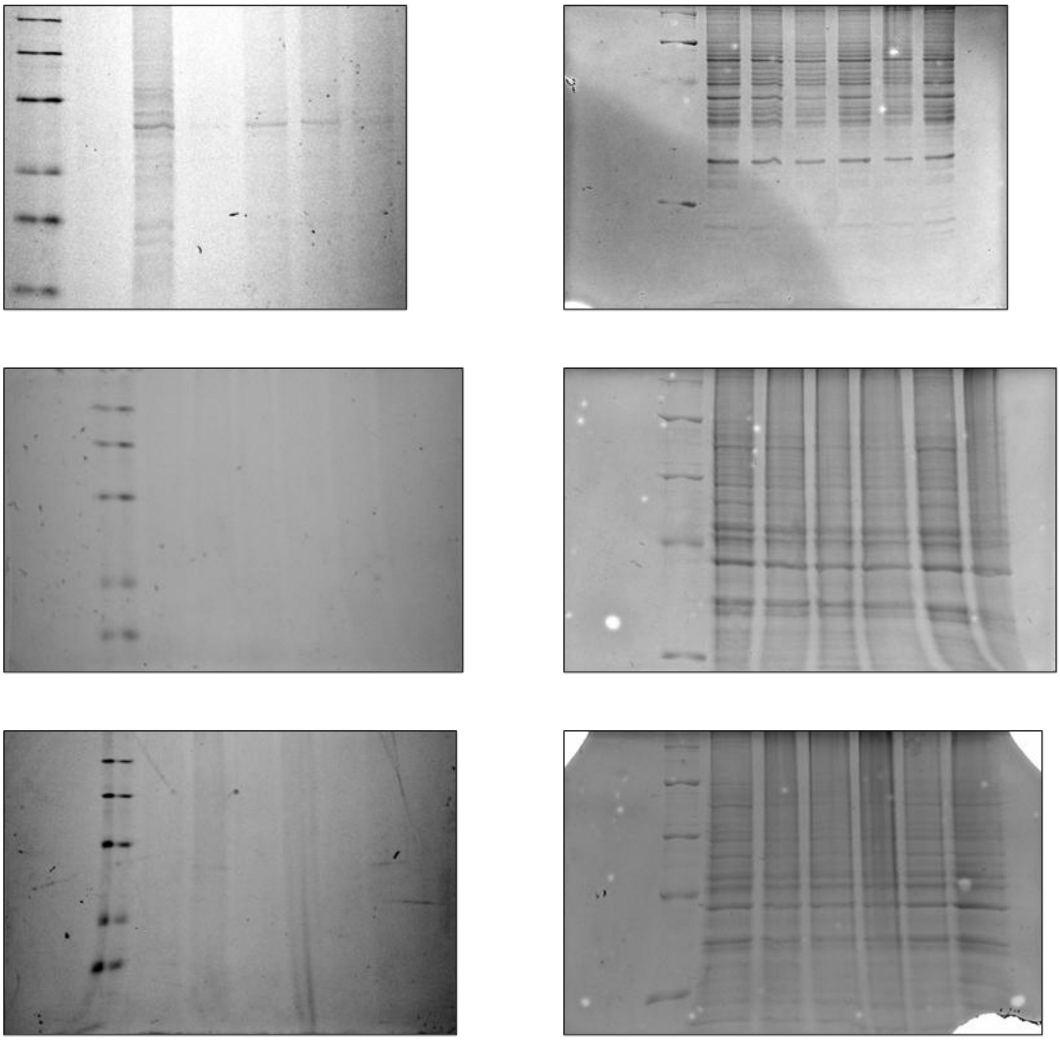

Figure S 40: Uncropped Gels of **Figure S21**.

References

1. Schneider, C. A., Rasband, W. S. & Eliceiri, K. W. NIH Image to ImageJ: 25 years of image analysis. *Nature methods* **9**, 671–675 (2012).
2. Gabrielsen, M. *et al.* Expression, purification, crystallization and initial X-ray diffraction analysis of thiol peroxidase from Yersinia pseudotuberculosis. *Acta Crystallographica Section F: Structural Biology and Crystallization Communications* **66**, 1606–1609 (2010).
3. Debowski, A. W., Verbrugghe, P., Sehnal, M., Marshall, B. J. & Benghezal, M. Development of a tetracycline-inducible gene expression system for the study of Helicobacter pylori pathogenesis. *Applied and environmental microbiology* **79**, 7351–7359 (2013).
4. Choi, J., Choi, S., Cha, M.-K., Kim, I.-H. & Shin, W. Crystallization and preliminary X-ray analysis of Escherichia coli p20, a novel thiol peroxidase. *Acta Crystallographica Section D: Biological Crystallography* **59**, 1064–1066 (2003).
5. Holm, L. Dali server: structural unification of protein families. *Nucleic Acids Research* **50**, W210–W215 (2022). https://doi.org/10.1093/nar/gkac387
6. Yang, L.-X. & Hofer, K. Synthesis of 2-Methyl-5-nitroimidazol-1-yl-acetaldehyde. *Synthetic communications* **26**, 3653–3657 (1996).
7. Ratsch, F. *et al.* Total Synthesis of α-Tocopherol through Enantioselective Iridium-Catalyzed Fragmentation of a Spiro-Cyclobutanol Intermediate. *Chemistry–A European Journal* **25**, 4941–4945 (2019).
8. Jamshidi, H. *et al.* Synthesis and biological activity profile of novel triazole/quinoline hybrids. *Chemical Biology & Drug Design* **100**, 935–946 (2022).
9. Bejot, R., Carroll, L., Bhakoo, K., Declerck, J. & Gouverneur, V. A fluorous and click approach for screening potential PET probes: evaluation of potential hypoxia biomarkers. *Bioorganic & medicinal chemistry* **20**, 324–329 (2012).
10. Dailidiene, D., Dailide, G., Kersulyte, D. & Berg, D. E. Contraselectable streptomycin susceptibility determinant for genetic manipulation and analysis of Helicobacter pylori. *Applied and environmental microbiology* **72**, 5908–5914 (2006).
11. Cox, J. & Mann, M. MaxQuant enables high peptide identification rates, individualized ppb-range mass accuracies and proteome-wide protein quantification. *Nature biotechnology* **26**, 1367–1372 (2008).
12. Cox, J. *et al.* Andromeda: A Peptide Search Engine Integrated into the MaxQuant Environment. *Journal of Proteome Research* **10**, 1794–1805 (2011). https://doi.org/10.1021/pr101065j
13. Tyanova, S. *et al.* The Perseus computational platform for comprehensive analysis of (prote) omics data. *Nature methods* **13**, 731–740 (2016).
14. Consortium, T. U. UniProt: the Universal Protein Knowledgebase in 2023. *Nucleic Acids Research* **51**, D523–D531 (2022). https://doi.org/10.1093/nar/gkac1052
15. Zanon, P. R. *et al.* Profiling the proteome-wide selectivity of diverse electrophiles. (2021).
16. Kessner, D., Chambers, M., Burke, R., Agus, D. & Mallick, P. ProteoWizard: open source software for rapid proteomics tools development. *Bioinformatics* **24**, 2534–2536 (2008).

17 Kong, A. T., Leprevost, F. V., Avtonomov, D. M., Mellacheruvu, D. & Nesvizhskii, A. I. MSFragger: ultrafast and comprehensive peptide identification in mass spectrometry–based proteomics. *Nature methods* **14**, 513–520 (2017).

18 Yu, F. *et al.* Identification of modified peptides using localization-aware open search. *Nature communications* **11**, 4065 (2020).

19 Chang, H.-Y. *et al.* Crystal-C: a computational tool for refinement of open search results. *Journal of proteome research* **19**, 2511–2515 (2020).

20 Geiszler, D. J. *et al.* PTM-Shepherd: analysis and summarization of post-translational and chemical modifications from open search results. *Molecular & Cellular Proteomics* **20** (2021).

21 Teo, G. C., Polasky, D. A., Yu, F. & Nesvizhskii, A. I. Fast deisotoping algorithm and its implementation in the MSFragger search engine. *Journal of proteome research* **20**, 498–505 (2020).

22 da Veiga Leprevost, F. *et al.* Philosopher: a versatile toolkit for shotgun proteomics data analysis. *Nature methods* **17**, 869–870 (2020).

23 Yu, F., Haynes, S. E. & Nesvizhskii, A. I. IonQuant enables accurate and sensitive label-free quantification with FDR-controlled match-between-runs. *Molecular & Cellular Proteomics* **20** (2021).

24 Wright, M. H. *et al.* Validation of N-myristoyltransferase as an antimalarial drug target using an integrated chemical biology approach. *Nature chemistry* **6**, 112–121 (2014).

25 Leitsch, D., Kolarich, D., Wilson, I. B. H., Altmann, F. & Duchêne, M. Nitroimidazole action in Entamoeba histolytica: a central role for thioredoxin reductase. *PLoS biology* **5**, e211 (2007).

26 Mendoza, J. A., Ignacio, J. L. & Buckley, C. M. The Hsp60 protein of Helicobacter pylori exhibits chaperone and ATPase activities at elevated temperatures. *BioChem* **1**, 19–25 (2021).

27 Nguyen, H. T. M., Nam, K.-H., Saleem, Y. & Kim, K.-S. Characterization of Helicobacter pylori adhesin thiol peroxidase (HP0390) purified from Escherichia coli. *Journal of biosciences* **35**, 241–248 (2010).

28 Kabsch, W. xds. *Acta Crystallographica Section D: Biological Crystallography* **66**, 125–132 (2010).

29 McCoy, A. J. *et al.* Phaser crystallographic software. *Journal of applied crystallography* **40**, 658–674 (2007).

30 Jumper, J. *et al.* Highly accurate protein structure prediction with AlphaFold. *Nature* **596**, 583–589 (2021).

31 Emsley, P., Lohkamp, B., Scott, W. G. & Cowtan, K. Features and development of Coot. *Acta Crystallographica Section D: Biological Crystallography* **66**, 486–501 (2010).

32 Perrakis, A., Sixma, T., Wilson, K. & Lamzin, V. wARP: improvement and extension of crystallographic phases by weighted averaging of multiple-refined dummy atomic models. *Acta Crystallographica Section D: Biological Crystallography* **53**, 448–455 (1997).

33 Vagin, A. A. *et al.* REFMAC5 dictionary: organization of prior chemical knowledge and guidelines for its use. *Acta Crystallographica Section D: Biological Crystallography* **60**, 2184–2195 (2004).

34 Rho, B.-S. *et al.* Functional and Structural Characterization of a Thiol Peroxidase from Mycobacterium tuberculosis. *Journal of Molecular Biology* **361**, 850–863 (2006). https://doi.org/https://doi.org/10.1016/j.jmb.2006.05.076

35 Rohdewald, P. & Beil, W. In vitro inhibition of helicobacter pylori growth and adherence to gastric mucosal cells by Pycnogenol®. *Phytotherapy Research: An International Journal Devoted to Pharmacological and Toxicological Evaluation of Natural Product Derivatives* **22**, 685–688 (2008).

36 Huang, Y., Wang, Q.-l., Cheng, D.-d., Xu, W.-t. & Lu, N.-h. Adhesion and invasion of gastric mucosa epithelial cells by Helicobacter pylori. *Frontiers in cellular and infection microbiology* **6**, 159 (2016).

37 Lozniewski, A. *et al.* Influence of Lewis antigen expression by Helicobacter pylori on bacterial internalization by gastric epithelial cells. *Infection and immunity* **71**, 2902–2906 (2003).

38 Zhang, Y., Huo, M., Zhou, J. & Xie, S. PKSolver: An add-in program for pharmacokinetic and pharmacodynamic data analysis in Microsoft Excel. *Computer methods and programs in biomedicine* **99**, 306–314 (2010).

39 Mała, P. *et al.* Discovery of N-β-l-Fucosyl Amides as High-Affinity Ligands for the Pseudomonas aeruginosa Lectin LecB. *Journal of Medicinal Chemistry* **65**, 14180–14200 (2022). https://doi.org/10.1021/acs.jmedchem.2c01373
