# Supplementary material for: Metronidazole and ether derivatives target Helicobacter pylori via simultaneous stress induction and inhibition
Source: Nat Microbiol. 2026 Mar 18;11(4):1049–63. doi: 10.1038/s41564-026-02291-w (PMC13056558; doi:10.1038/s41564-026-02291-w)
Supplement: Supplementary file 2 — Reporting summary [file 41564_2026_2291_MOESM2_ESM.pdf]

nature portfolio

Corresponding author(s): Stephan A. Sieber

Last updated by author(s): Jan 26, 2026

Reporting Summary

Nature Portfolio wishes to improve the reproducibility of the work that we publish. This form provides structure for consistency and transparency in reporting. For further information on Nature Portfolio policies, see our Editorial Policies and the Editorial Policy Checklist.

Statistics

For all statistical analyses, confirm that the following items are present in the figure legend, table legend, main text, or Methods section.

n/a	Confirmed	
☐	☒	The exact sample size (*n*) for each experimental group/condition, given as a discrete number and unit of measurement
☐	☒	A statement on whether measurements were taken from distinct samples or whether the same sample was measured repeatedly
☐	☒	The statistical test(s) used AND whether they are one- or two-sided<br>*Only common tests should be described solely by name; describe more complex techniques in the Methods section.*
☐	☒	A description of all covariates tested
☐	☒	A description of any assumptions or corrections, such as tests of normality and adjustment for multiple comparisons
☐	☒	A full description of the statistical parameters including central tendency (e.g. means) or other basic estimates (e.g. regression coefficient) AND variation (e.g. standard deviation) or associated estimates of uncertainty (e.g. confidence intervals)
☐	☒	For null hypothesis testing, the test statistic (e.g. *F*, *t*, *r*) with confidence intervals, effect sizes, degrees of freedom and *P* value noted<br>*Give P values as exact values whenever suitable.*
☒	☐	For Bayesian analysis, information on the choice of priors and Markov chain Monte Carlo settings
☒	☐	For hierarchical and complex designs, identification of the appropriate level for tests and full reporting of outcomes
☒	☐	Estimates of effect sizes (e.g. Cohen's *d*, Pearson's *r*), indicating how they were calculated

Our web collection on statistics for biologists contains articles on many of the points above.

Software and code

Policy information about availability of computer code

Data collection	no code was used
Data analysis	GraphPad Prism 10, Perseus 2.0.10.0 or 2.0.11.0, ImageJ, ChemDraw, MestReNova, FragPipe, MSFragger, MaxQuant 1.6.2.10

For manuscripts utilizing custom algorithms or software that are central to the research but not yet described in published literature, software must be made available to editors and reviewers. We strongly encourage code deposition in a community repository (e.g. GitHub). See the Nature Portfolio guidelines for submitting code & software for further information.

Data

Policy information about availability of data

All manuscripts must include a data availability statement. This statement should provide the following information, where applicable:

- Accession codes, unique identifiers, or web links for publicly available datasets
- A description of any restrictions on data availability
- For clinical datasets or third party data, please ensure that the statement adheres to our policy

Crystal structures have been deposited in the RCSB Protein Data Bank (PDB IDs: 2XPD, 9F5V, 3ZRD, 9F64, 9F65). The mass spectrometry proteomics data have been deposited to the ProteomeXchange Consortium via the PRIDE partner repository with the dataset identifier PXD051773 (H. pylori) and PXD067473 (S. epidermidis, S. pseudintermedius). 16S sequencing data have been deposited to ENA (PRJEB107125).

Research involving human participants, their data, or biological material

Policy information about studies with human participants or human data. See also policy information about sex, gender (identity/presentation), and sexual orientation and race, ethnicity and racism.

Reporting on sex and gender	no human participant involved in the present study
Reporting on race, ethnicity, or other socially relevant groupings	no human participant involved in the present study
Population characteristics	no human participant involved in the present study
Recruitment	no human participant involved in the present study
Ethics oversight	no human participant involved in the present study

Note that full information on the approval of the study protocol must also be provided in the manuscript.

Field-specific reporting

Please select the one below that is the best fit for your research. If you are not sure, read the appropriate sections before making your selection.

☒ Life sciences ☐ Behavioural & social sciences ☐ Ecological, evolutionary & environmental sciences

For a reference copy of the document with all sections, see nature.com/documents/nr-reporting-summary-flat.pdf

Life sciences study design

All studies must disclose on these points even when the disclosure is negative.

Sample size	Sample sizes were selected based on standard practice in the field. For in vivo mouse studies a statistician determined the sample size during the ethical approval process.
Data exclusions	No data was excluded.
Replication	Exact replicate numbers are indicated individually for each experiment in the manuscript. All attempts at replication were successful.
Randomization	Randomization was not performed/necessary.
Blinding	Blinding was not performed/necessary.

Reporting for specific materials, systems and methods

We require information from authors about some types of materials, experimental systems and methods used in many studies. Here, indicate whether each material, system or method listed is relevant to your study. If you are not sure if a list item applies to your research, read the appropriate section before selecting a response.

Materials & experimental systems

n/a	Involved in the study	
☒	☐	Antibodies
☐	☒	Eukaryotic cell lines
☒	☐	Palaeontology and archaeology
☐	☒	Animals and other organisms
☒	☐	Clinical data
☒	☐	Dual use research of concern
☒	☐	Plants

Methods

n/a	Involved in the study	
☒	☐	ChIP-seq
☒	☐	Flow cytometry
☒	☐	MRI-based neuroimaging

Eukaryotic cell lines

Policy information about cell lines and Sex and Gender in Research

Cell line source(s)	HeLa, AGS and HepG2 were bought from ATCC or DSMZ

Authentication	was not authenticated
Mycoplasma contamination	cell lines were tested for mycoplasma contamination prior to experiments
Commonly misidentified lines (See ICLAC register)	there was no commonly misidentified line used

Animals and other research organisms

Policy information about studies involving animals; ARRIVE guidelines recommended for reporting animal research, and Sex and Gender in Research

Laboratory animals	male CD-1 mice (Charles River, Germany) and Female C57BL/6 wild-type mice (Envigo)
Wild animals	no wild animals used
Reporting on sex	sex was not considered in particular
Field-collected samples	study did not involve sample collection
Ethics oversight	The animal studies were conducted in accordance with the recommendations of the European Community (Directive 2010/63/EU, 1st January 2013). All animal procedures were performed in strict accordance with the German regulations of the Society for Laboratory Animal Science (GV-SOLAS) and the European Health Law of the Federation of Laboratory Animal Science Associations (FELASA). Animals were excluded from further analysis if sacrifice was necessary according to the humane endpoints established by the ethical board. All experiments were approved by the ethical board of the Niedersächsisches Landesamt für Verbraucherschutz und Lebensmittelsicherheit, Oldenburg, or by the Bavarian government (Regierung von Oberbayern, permit no. ROB-55.2-2532.Vet_02-23-90). Animals were kept in individually ventilated cages with a 10h/14h dark/light cycle and had access to food and water ad libitum as well as additional nesting material. The animals were housed in individually ventilated cages in an animal facility with a room temperature of 21–22 °C and a relative humidity in the range of 45–65%.

Note that full information on the approval of the study protocol must also be provided in the manuscript.

Plants

Seed stocks	no plants were used in the present study
Novel plant genotypes	no plants were used in the present study
Authentication	no plants were used in the present study
