# Supplementary material for: Metronidazole and ether derivatives target Helicobacter pylori via simultaneous stress induction and inhibition
Source: Nat Microbiol. 2026 Mar 18;11(4):1049–63. doi: 10.1038/s41564-026-02291-w (PMC13056558; doi:10.1038/s41564-026-02291-w)
Supplement: Supplementary file 12 — All NMR spectra. [file 41564_2026_2291_MOESM12_ESM.pdf]

Metro-P1

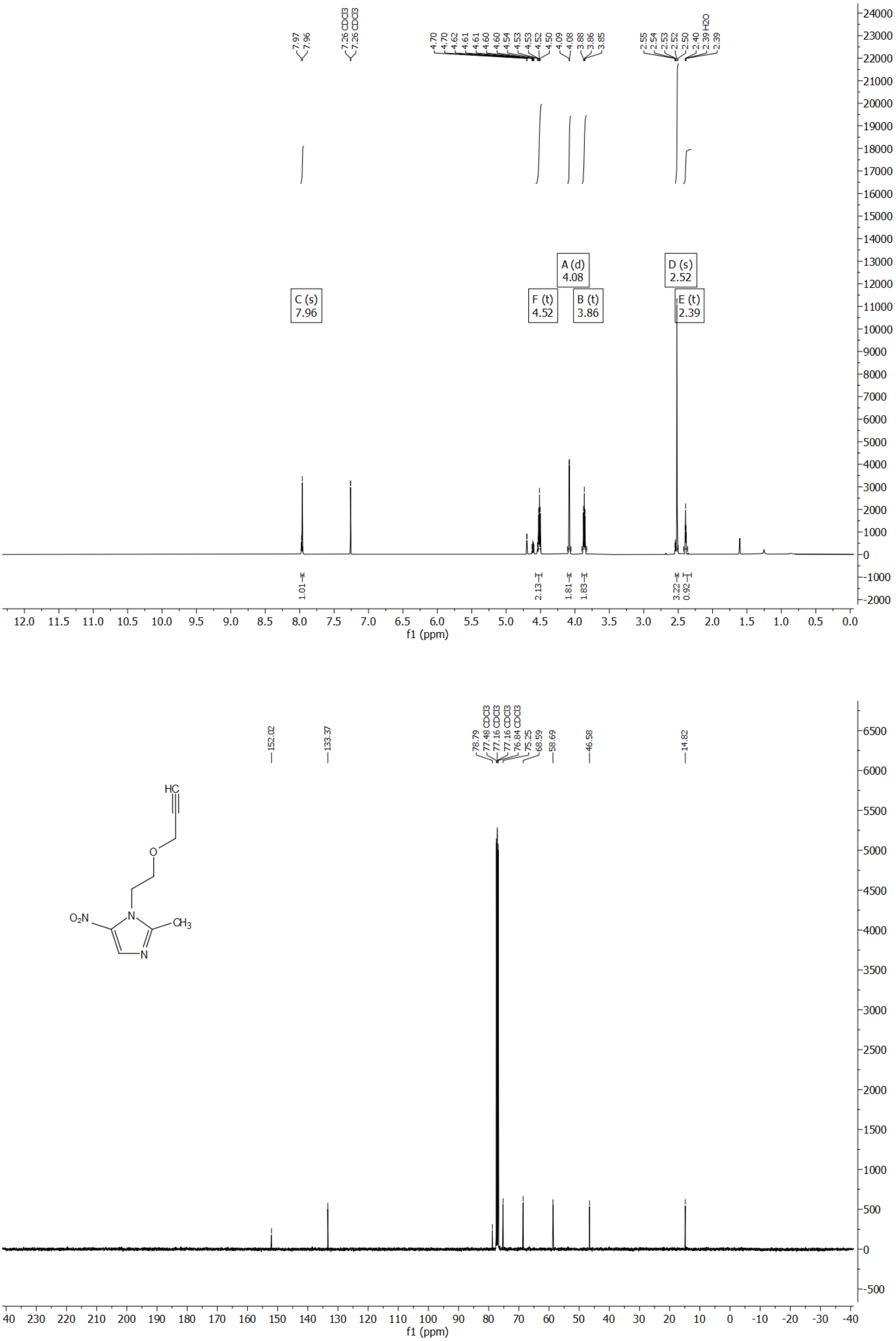

^{1}H and ^{13}C NMR spectra of **Metro-P1**.

Aldehyd 1

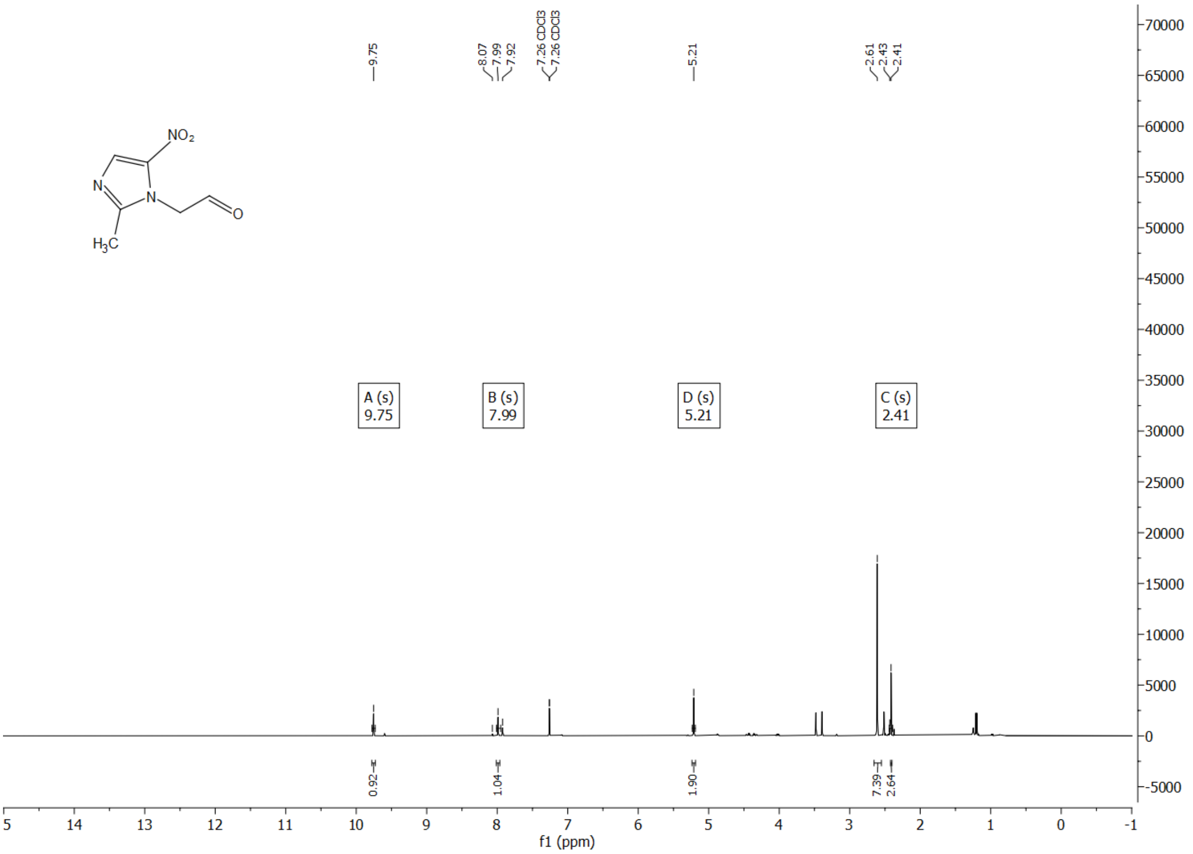

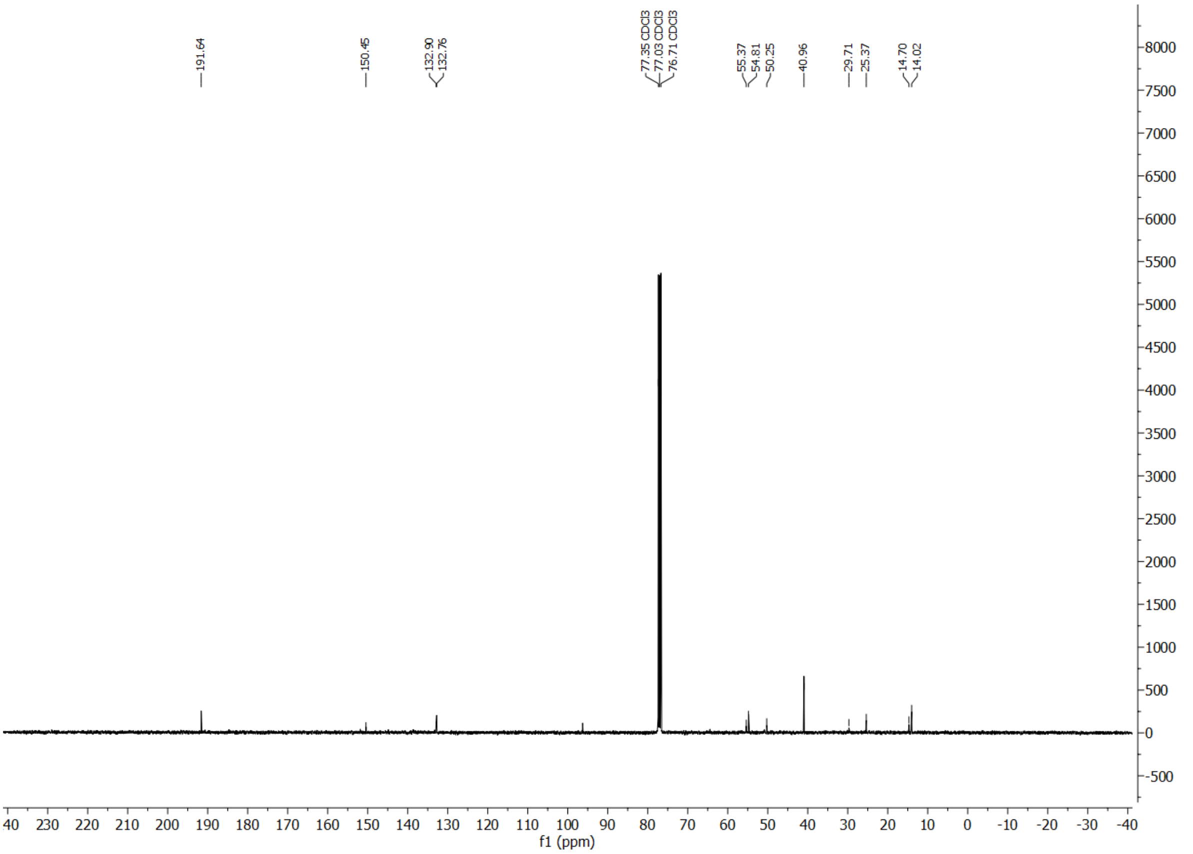

^{1}H and ^{13}C NMR spectra of **aldehyde 1**.

Metro-P2

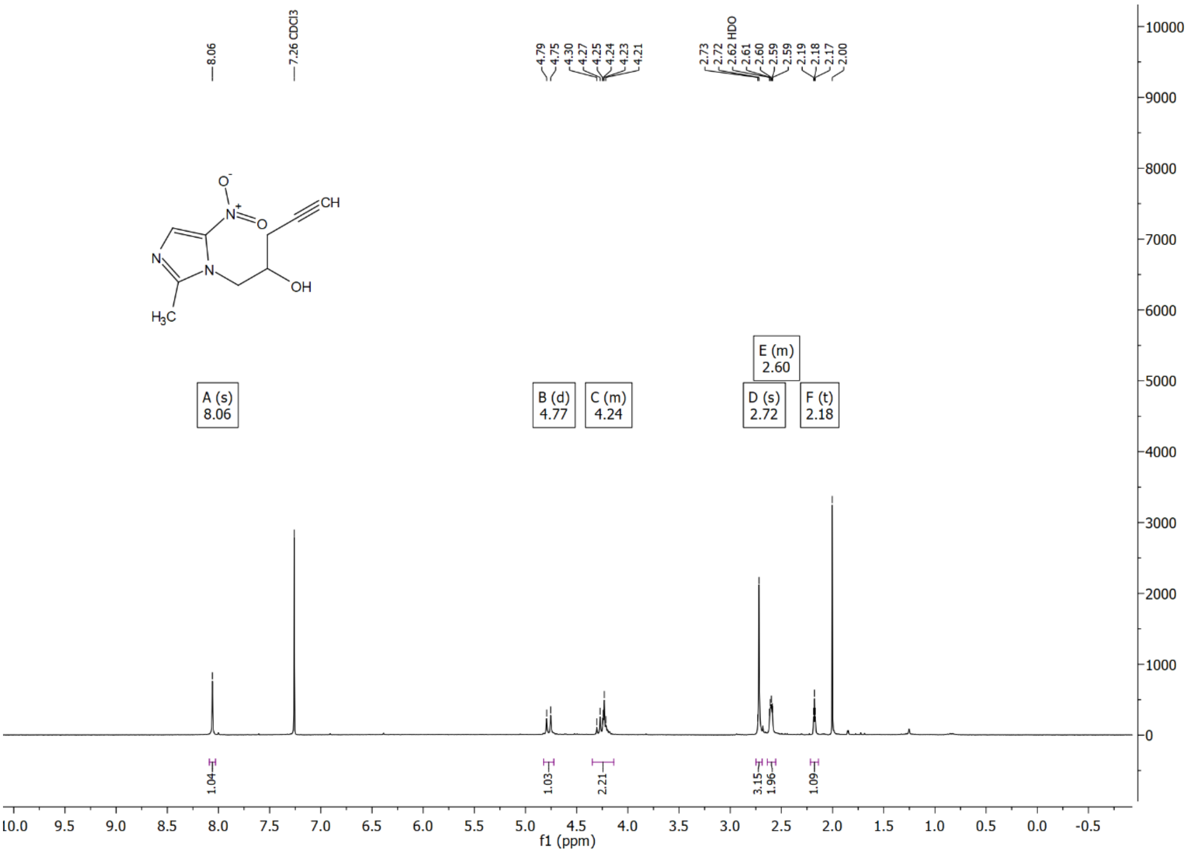

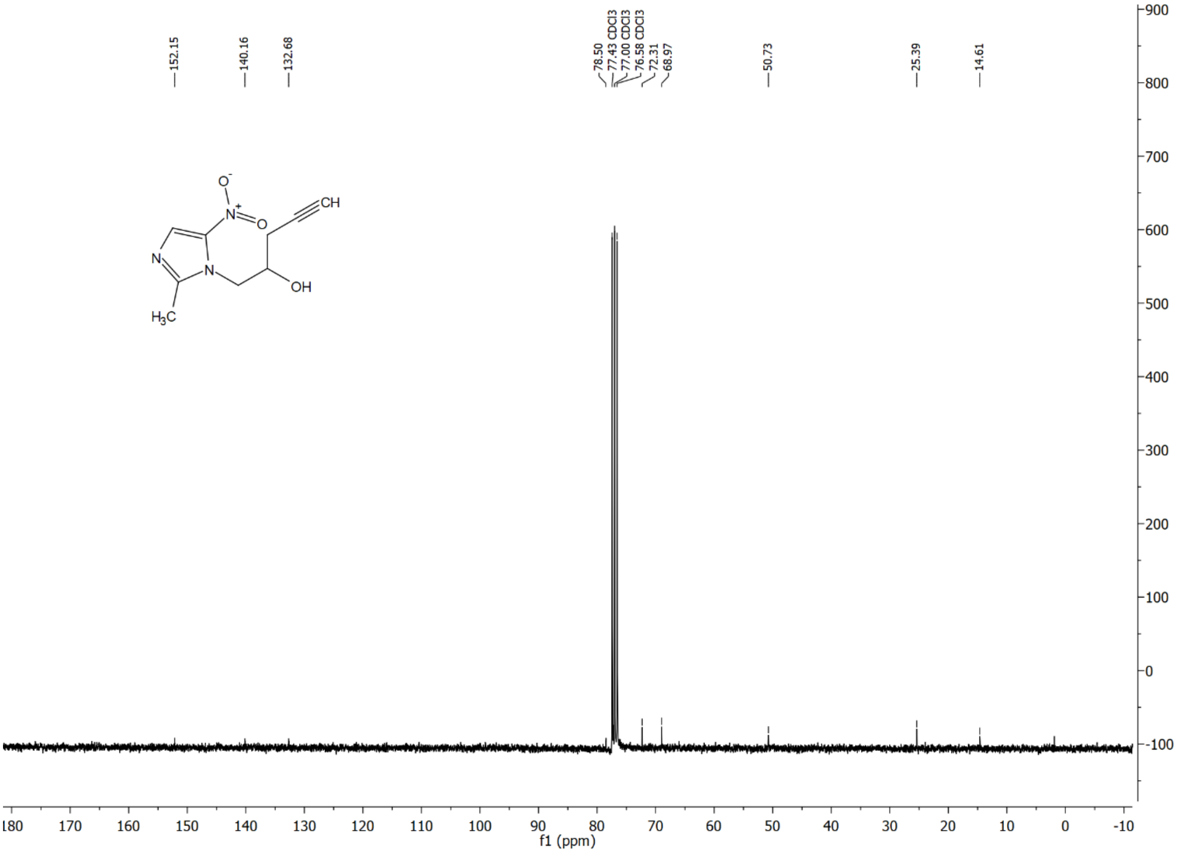

^{1}H and ^{13}C NMR spectra of **Metro-P2**.

Metro-P3

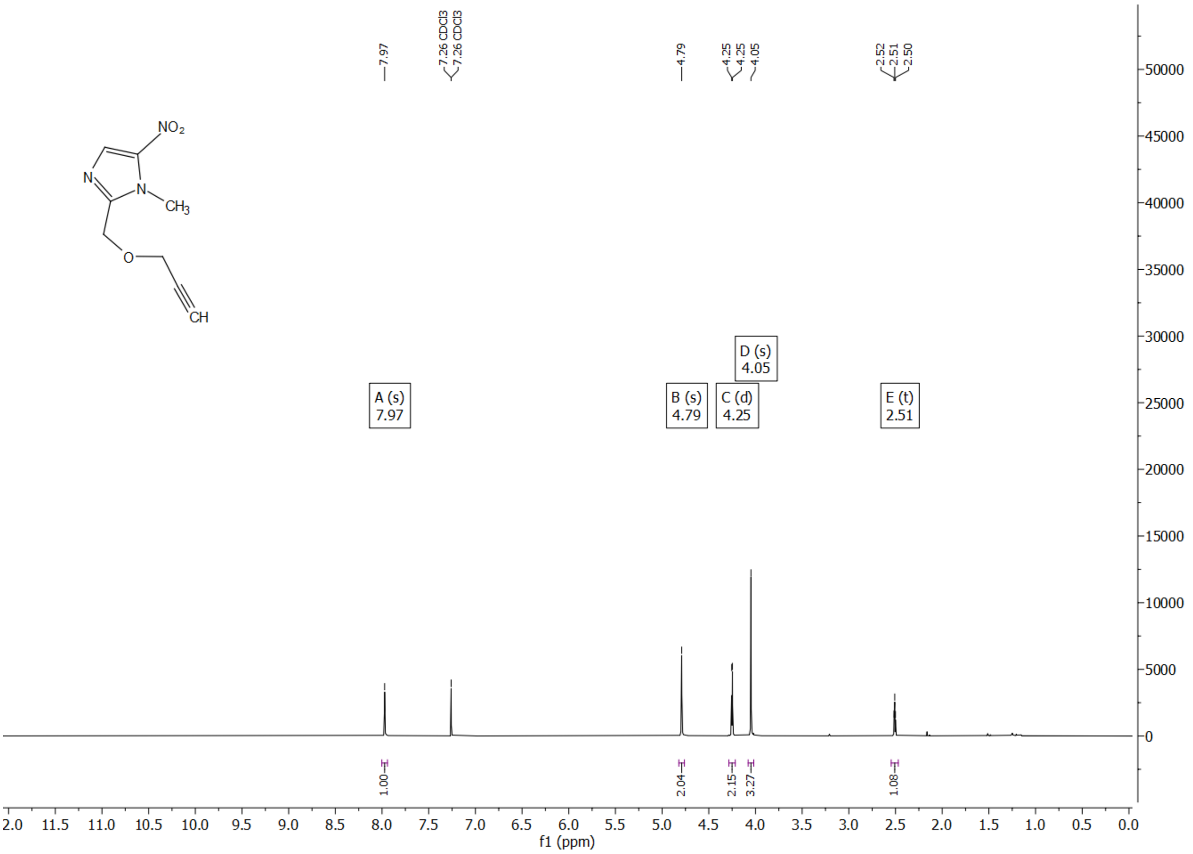

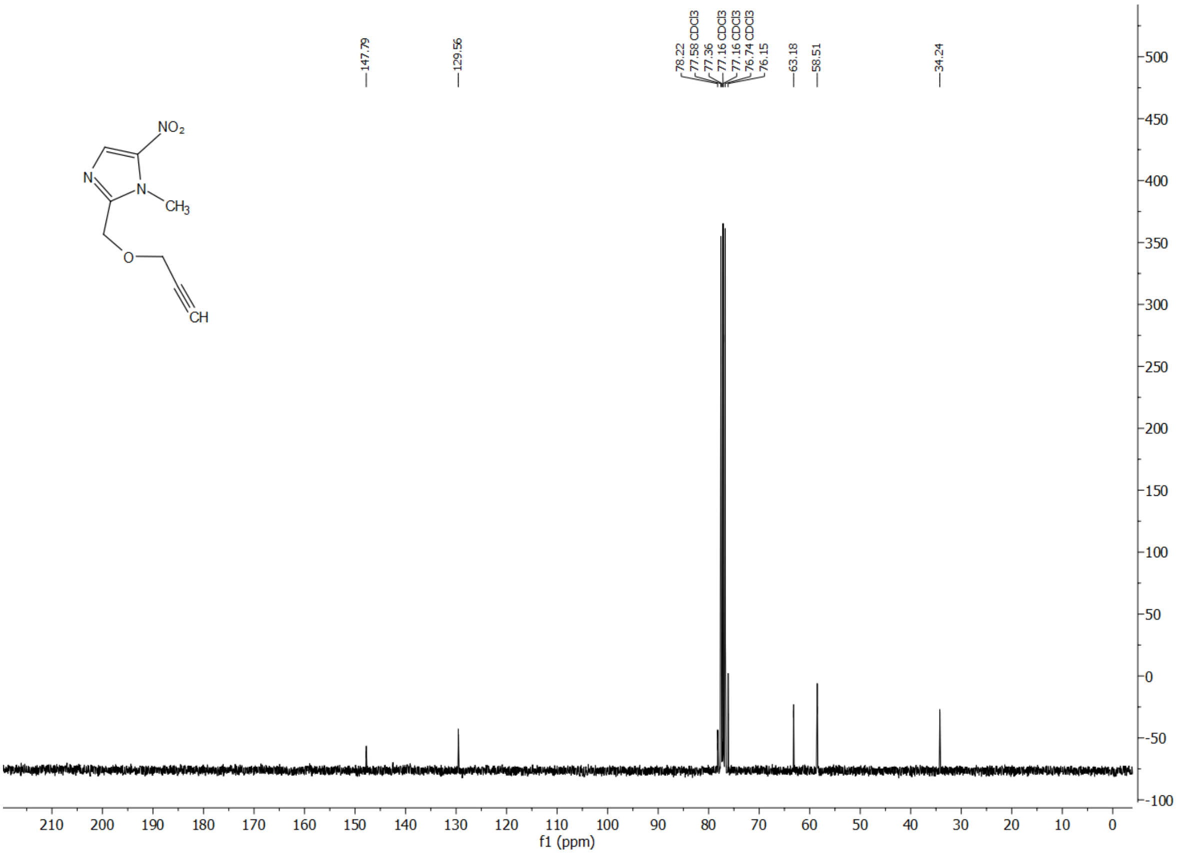

^{1}H and ^{13}C NMR spectra of **Metro-P3**.

Metro-P4

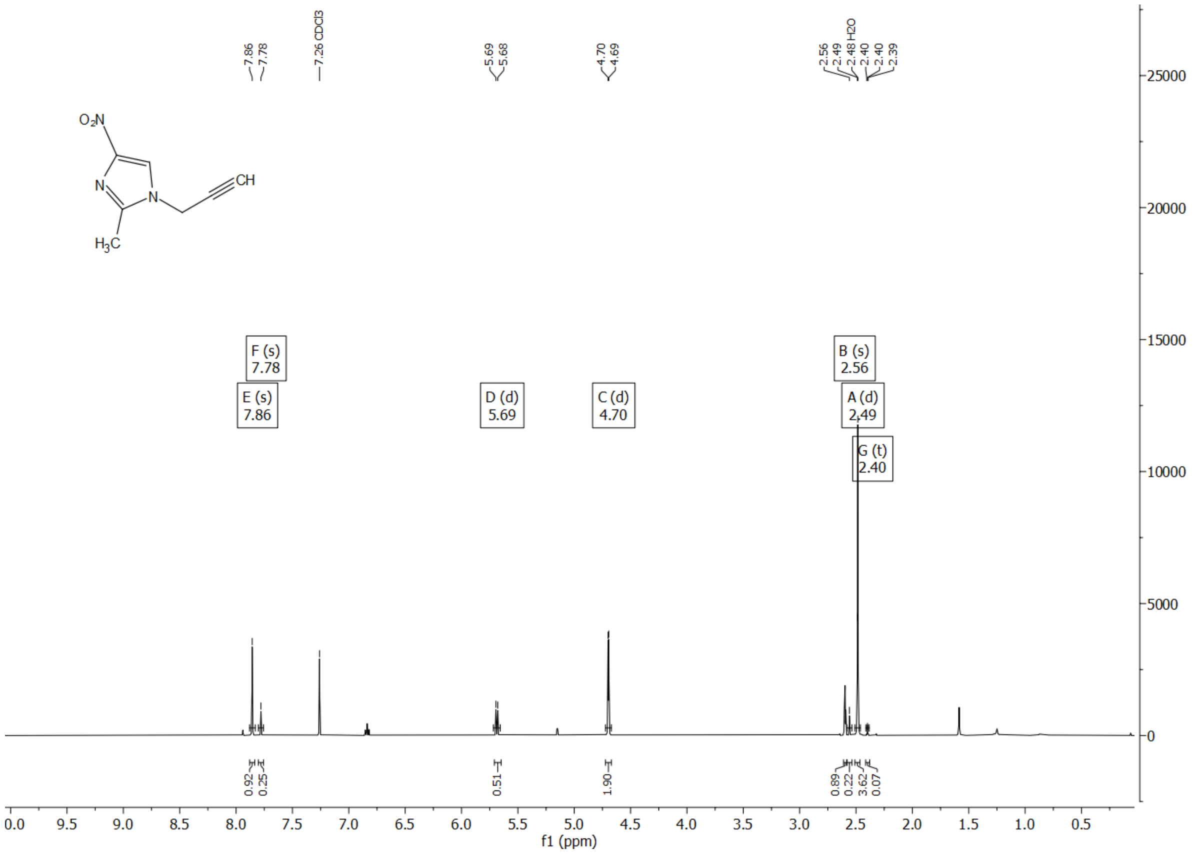

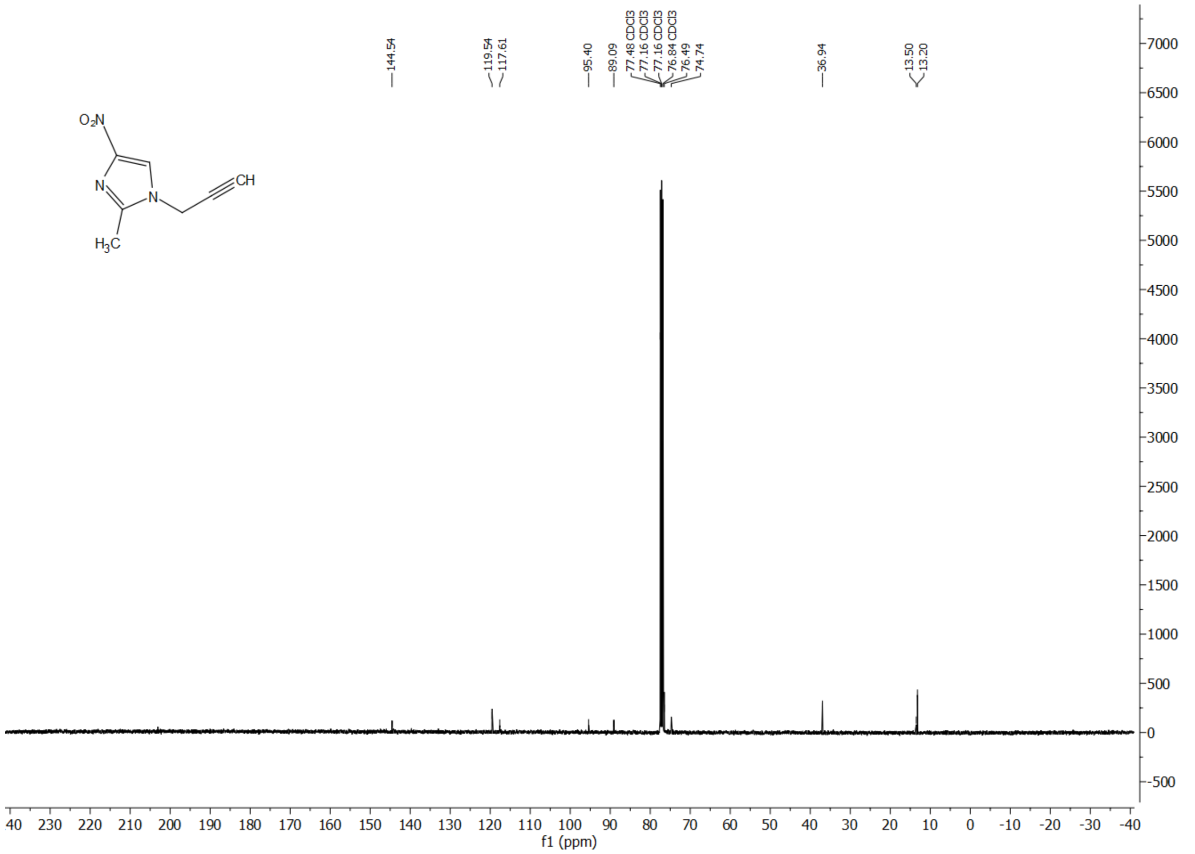

^{1}H and ^{13}C NMR spectra of **Metro-P4**.

MF-01

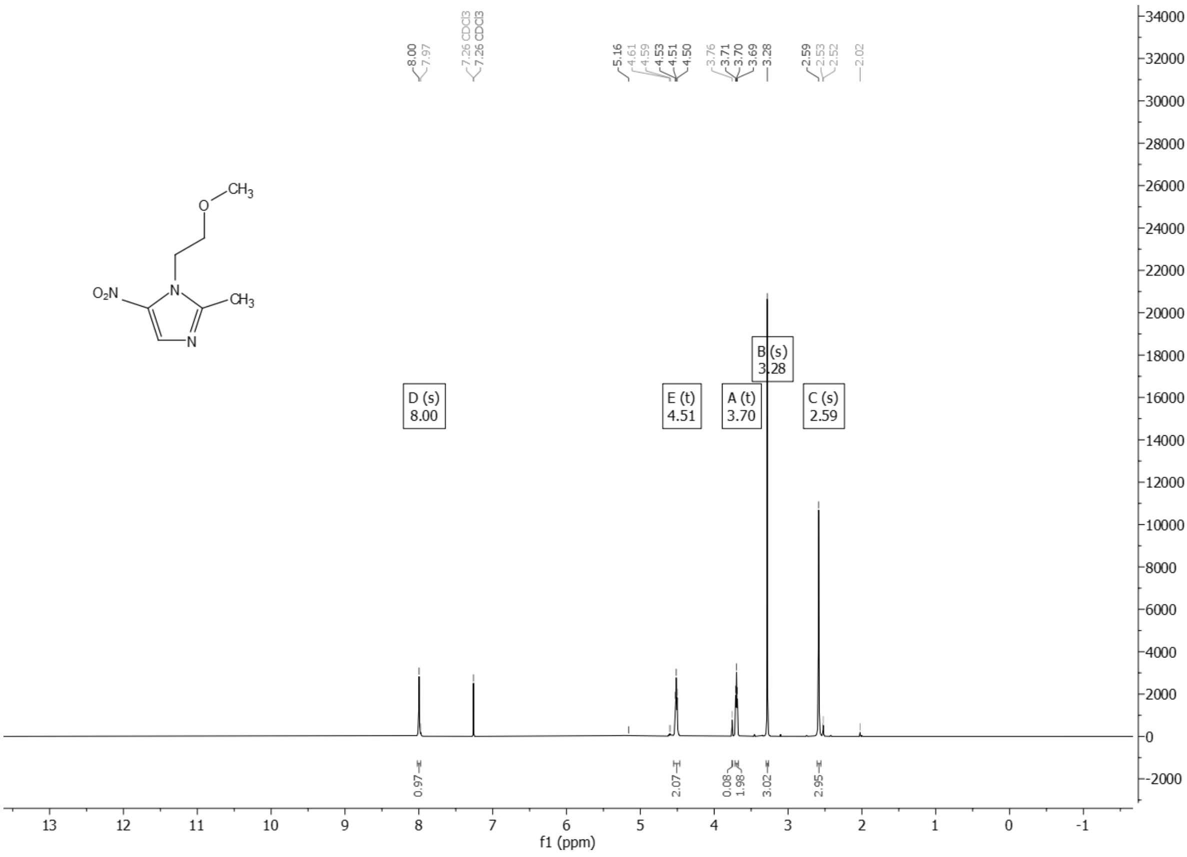

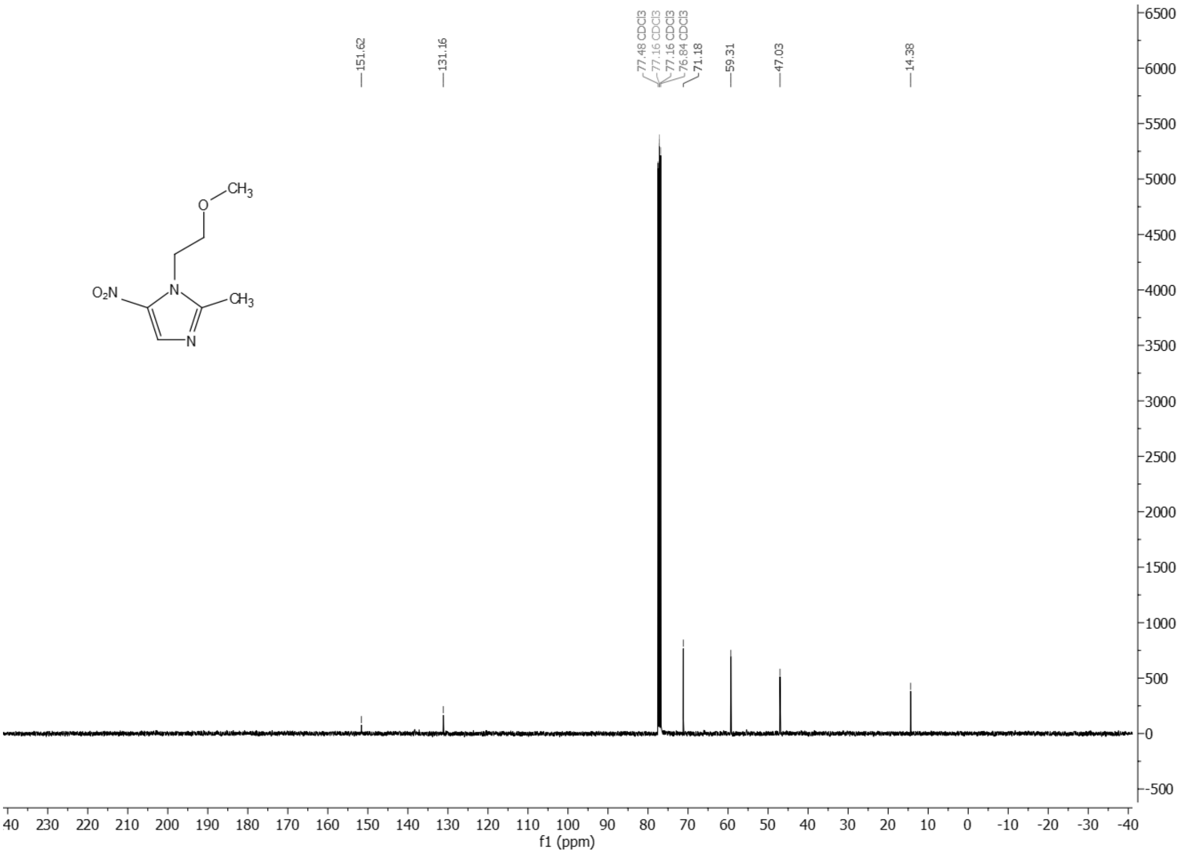

^{1}H and ^{13}C NMR spectra of **MF-01**.

MF-02

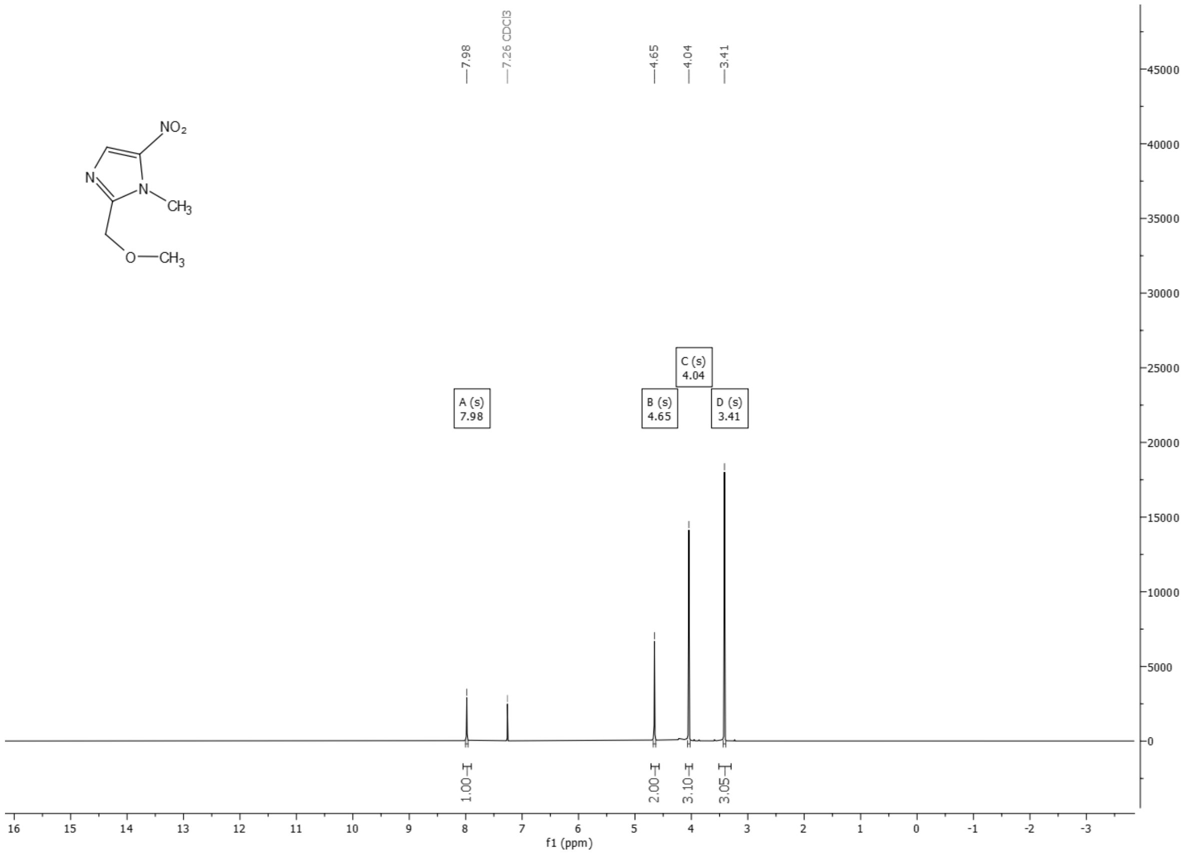

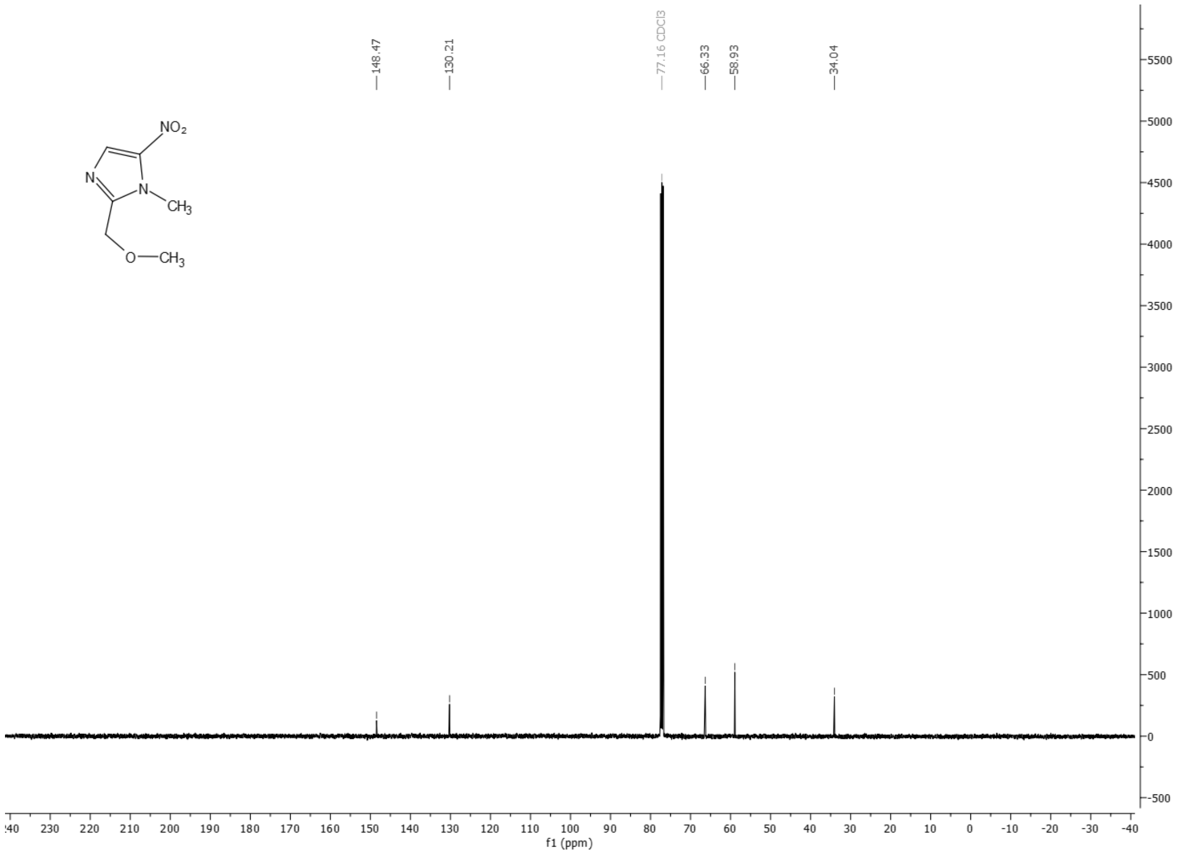

^{1}H and ^{13}C NMR spectra of **MF-02**.

MF-03

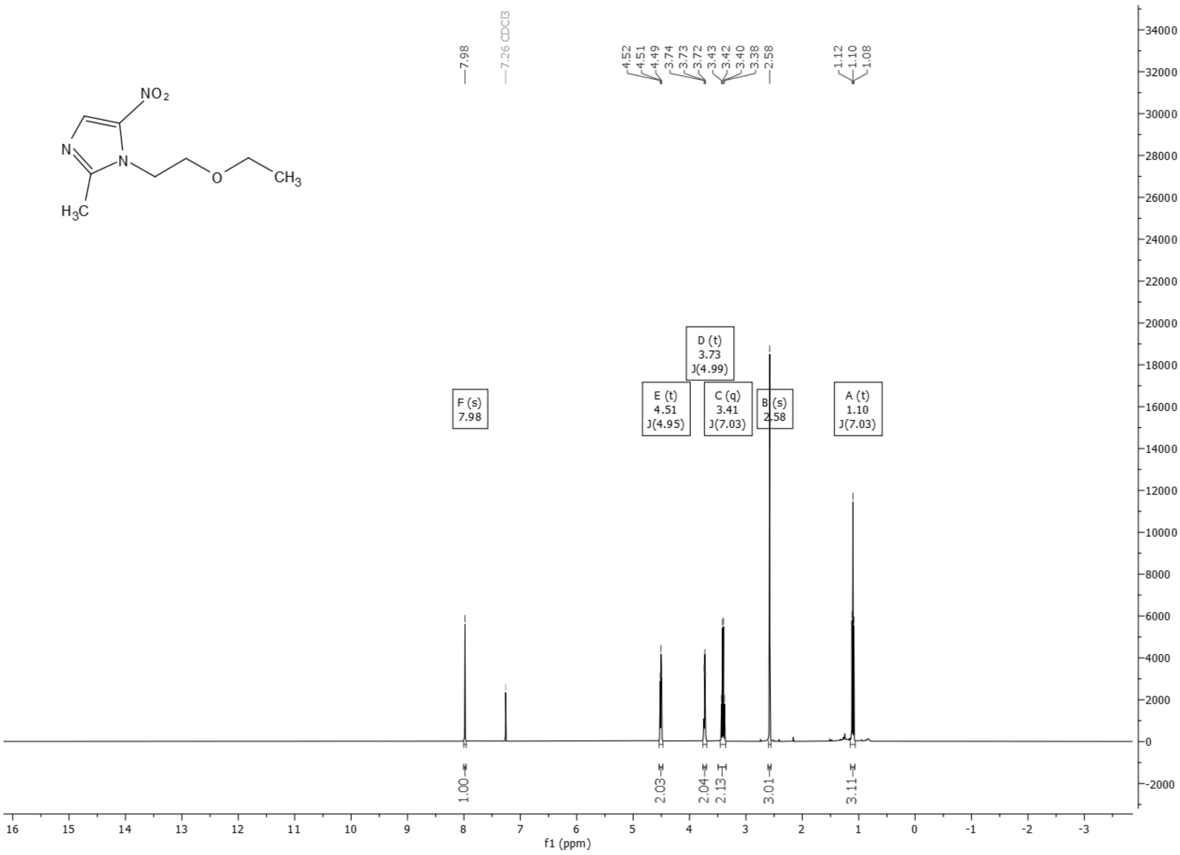

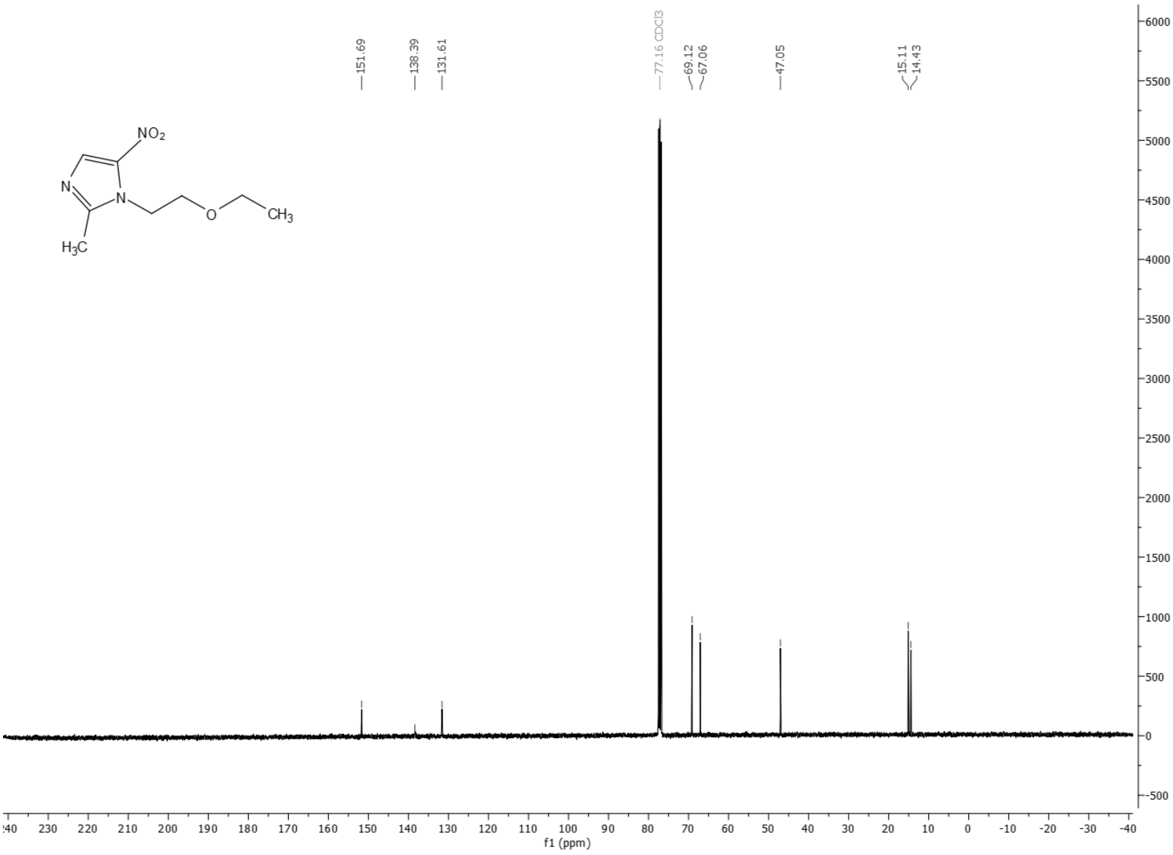

^{1}H and ^{13}C NMR spectra of **MF-03**.

MF-04

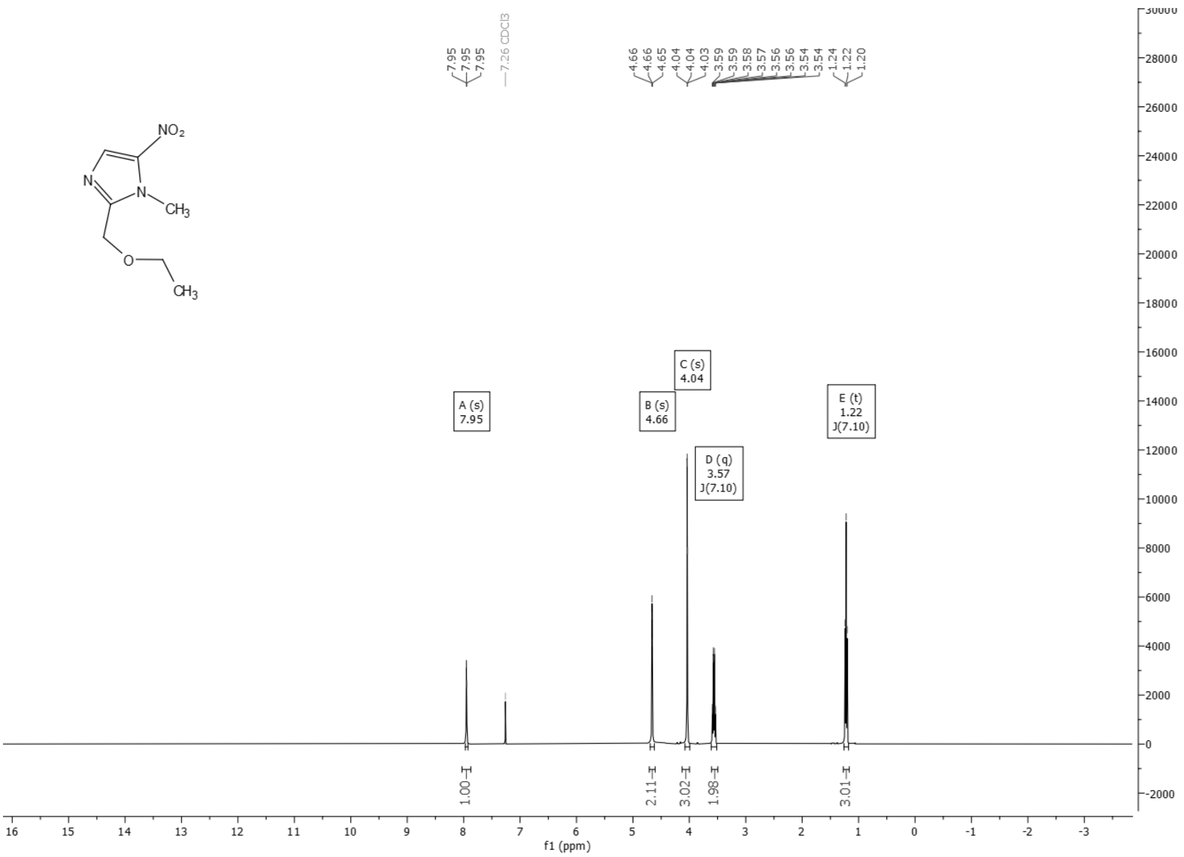

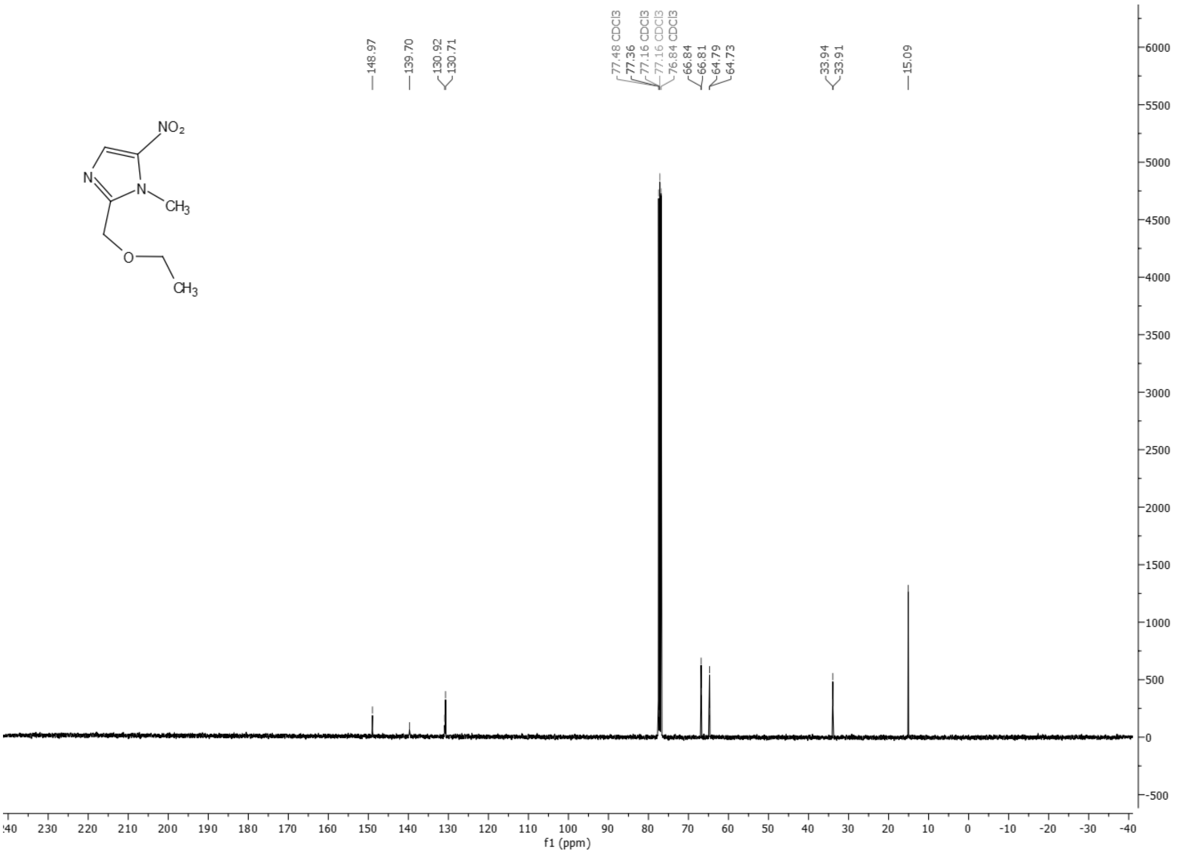

^{1}H and ^{13}C NMR spectra of **MF-04**.

MF-05

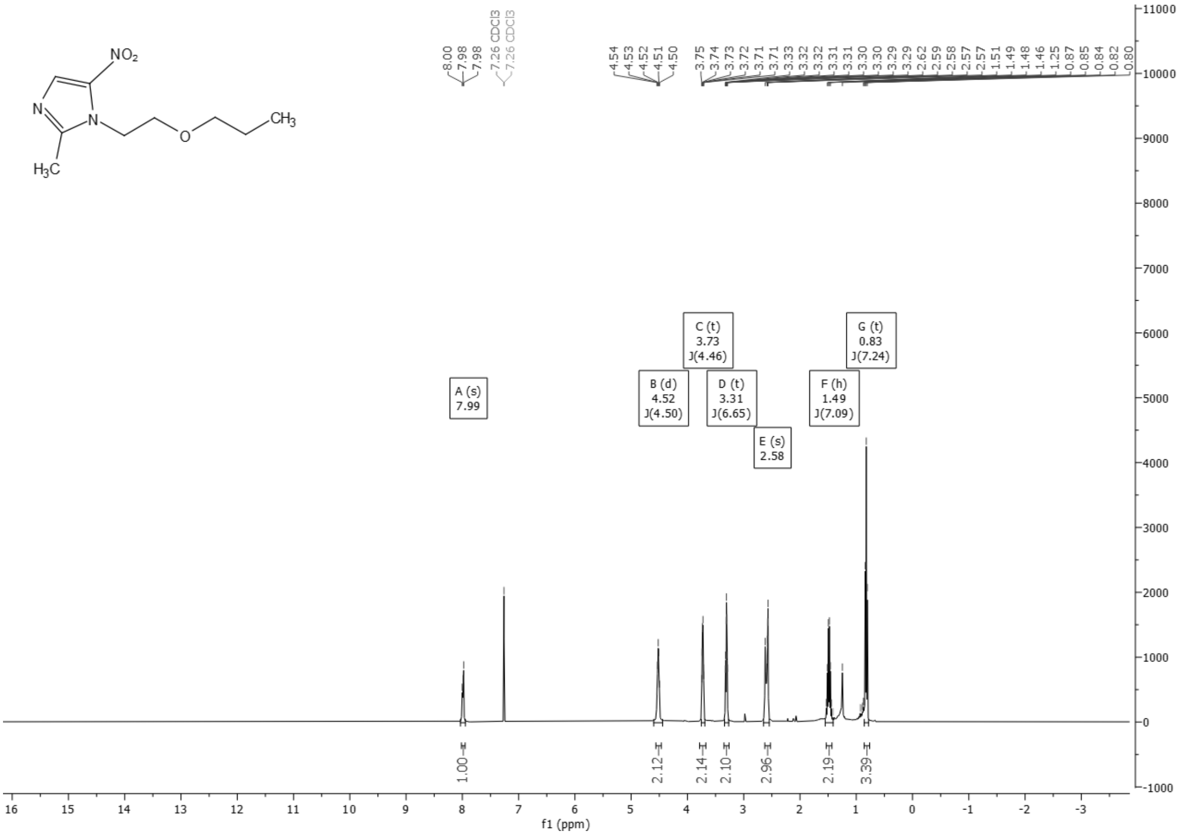

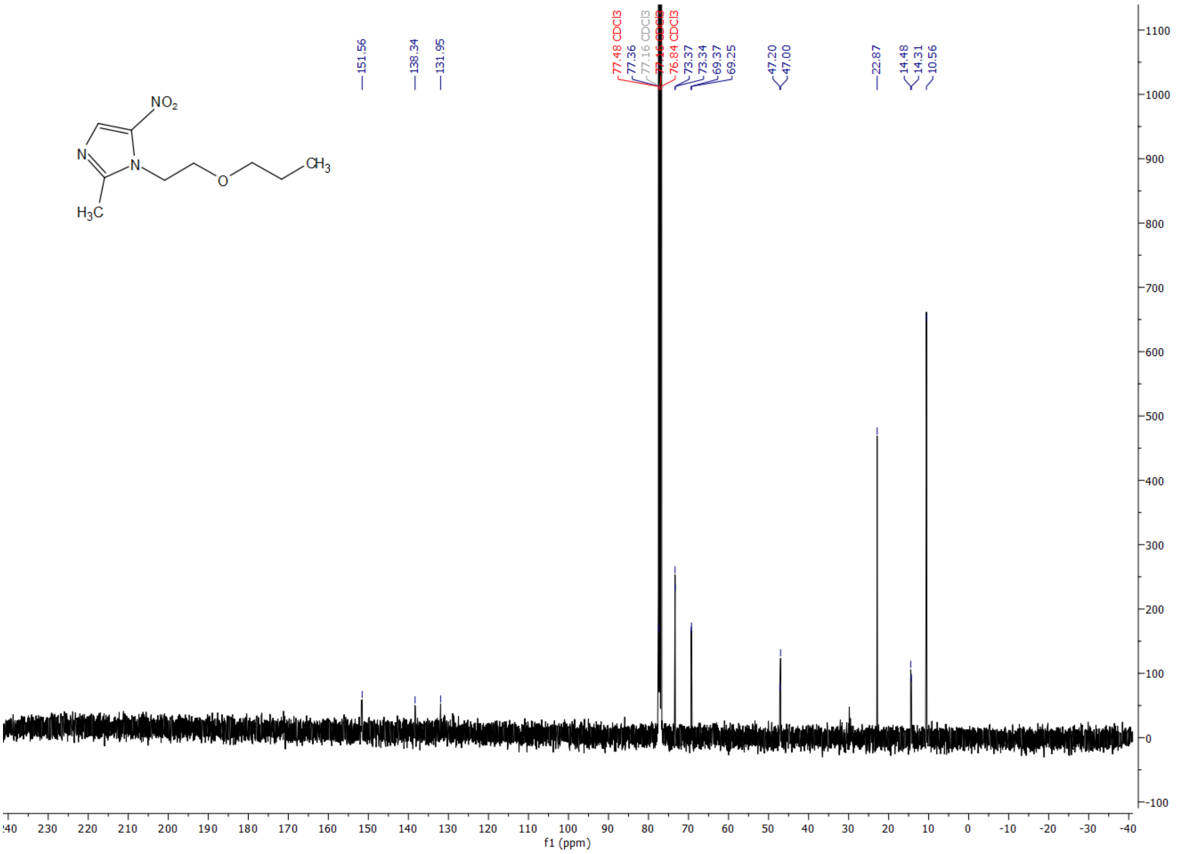

^1H and ^{13}C NMR spectra of **MF-05**.

MF-06

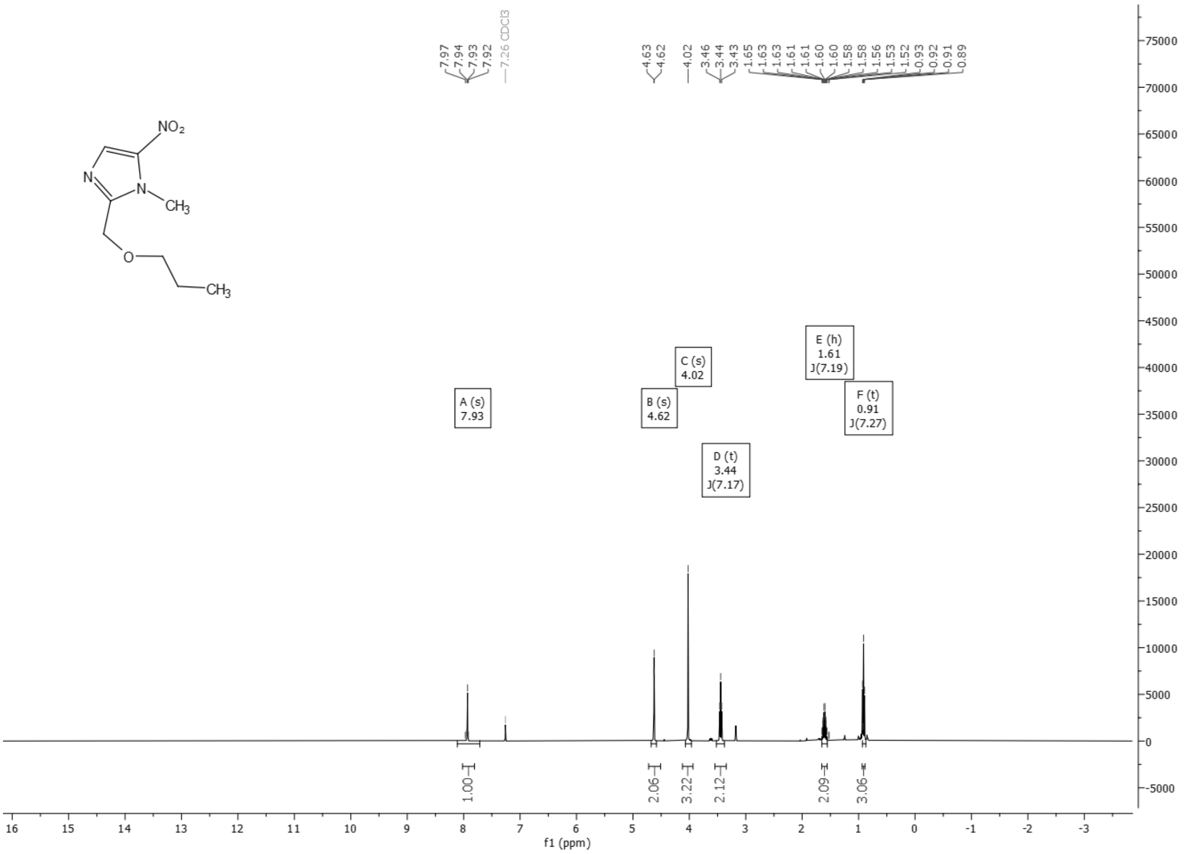

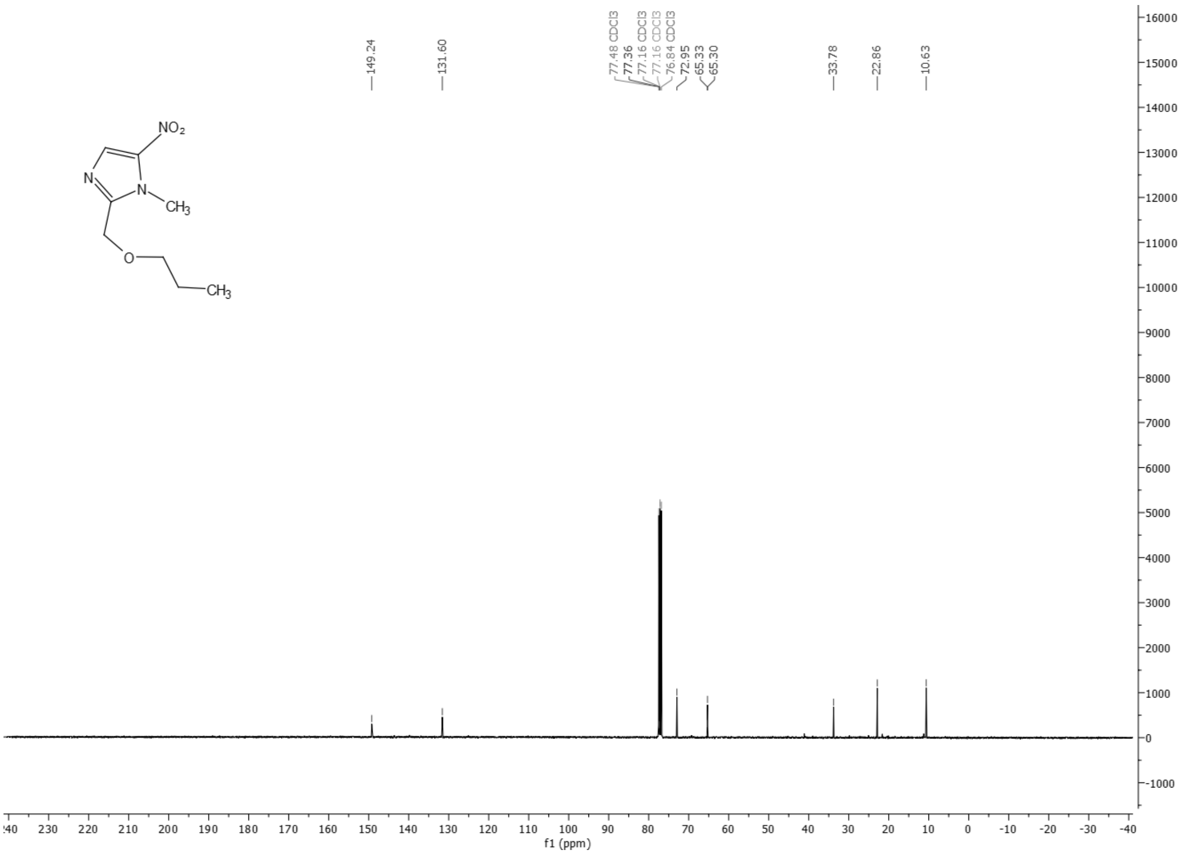

^{1}H and ^{13}C NMR spectra of **MF-06**.

MF-07

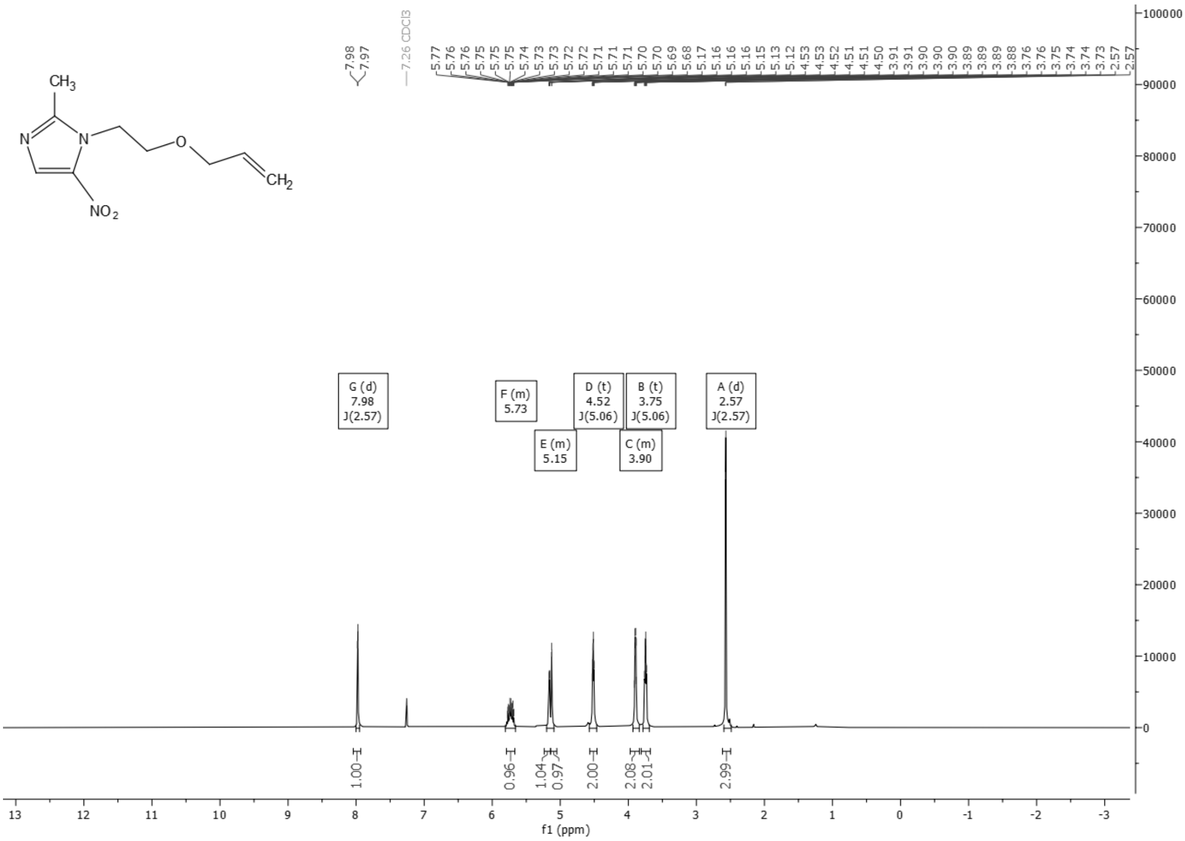

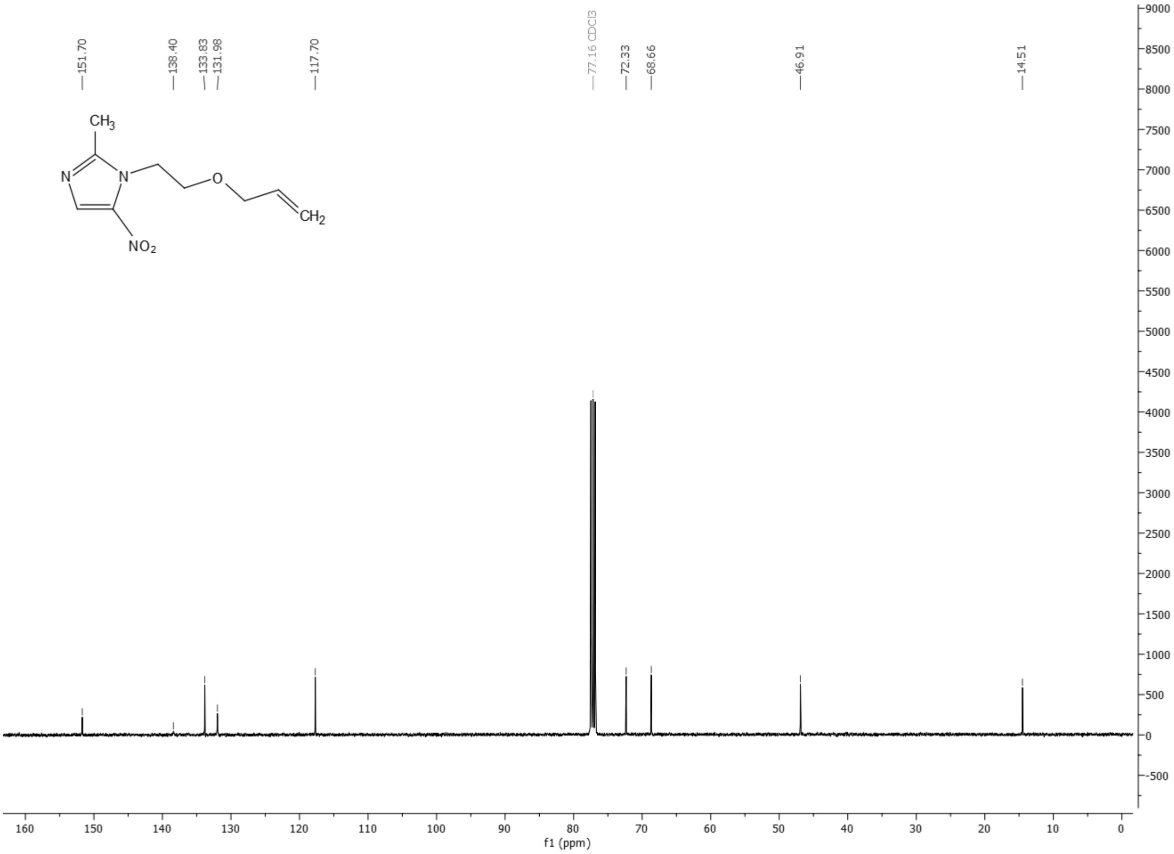

^{1}H and ^{13}C NMR spectra of **MF-07**.

MF-08

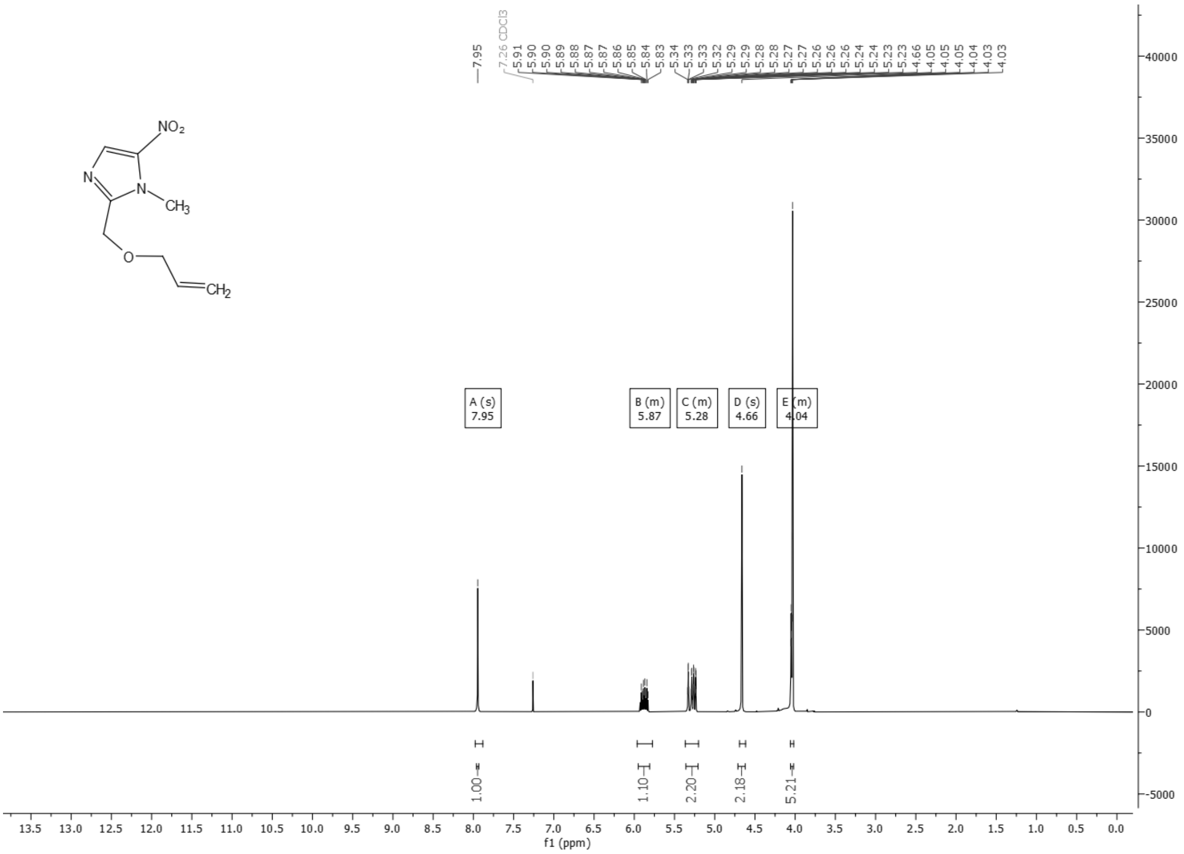

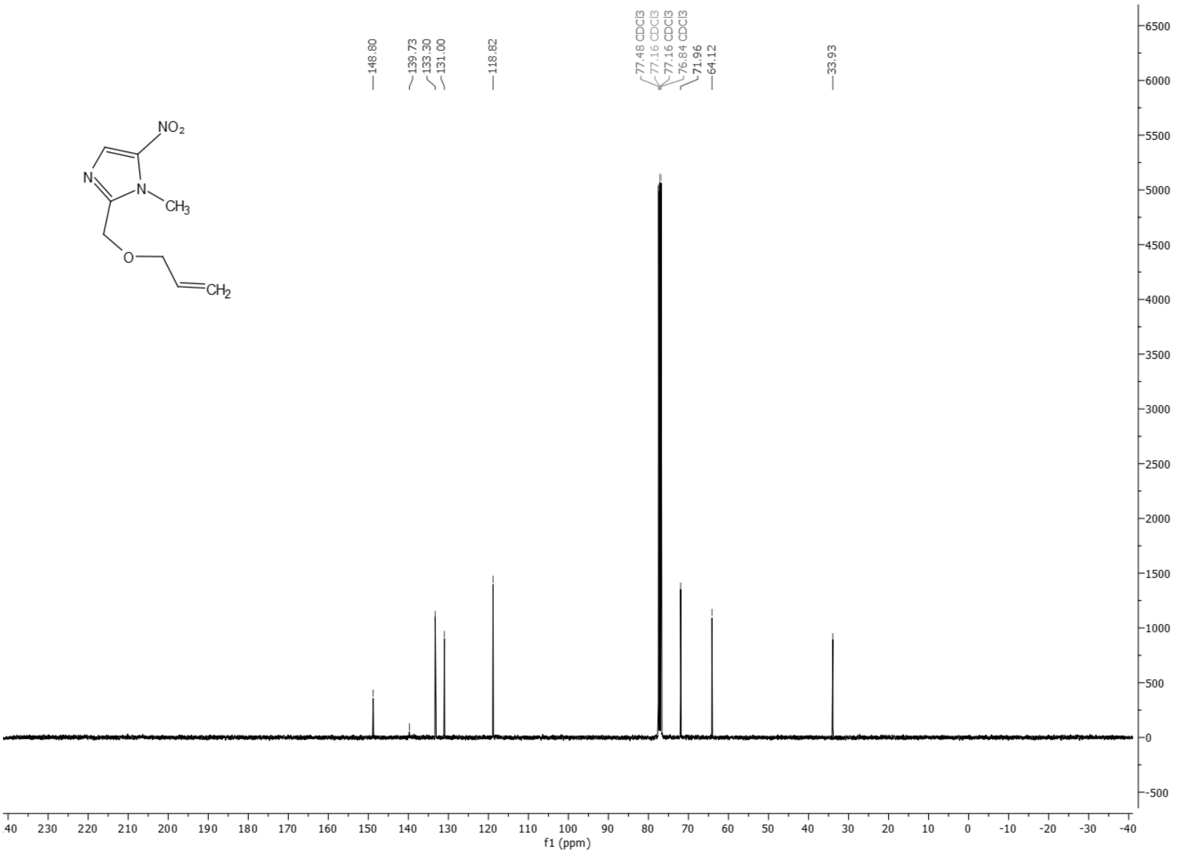

^{1}H and ^{13}C NMR spectra of **MF-08**.

MF-09

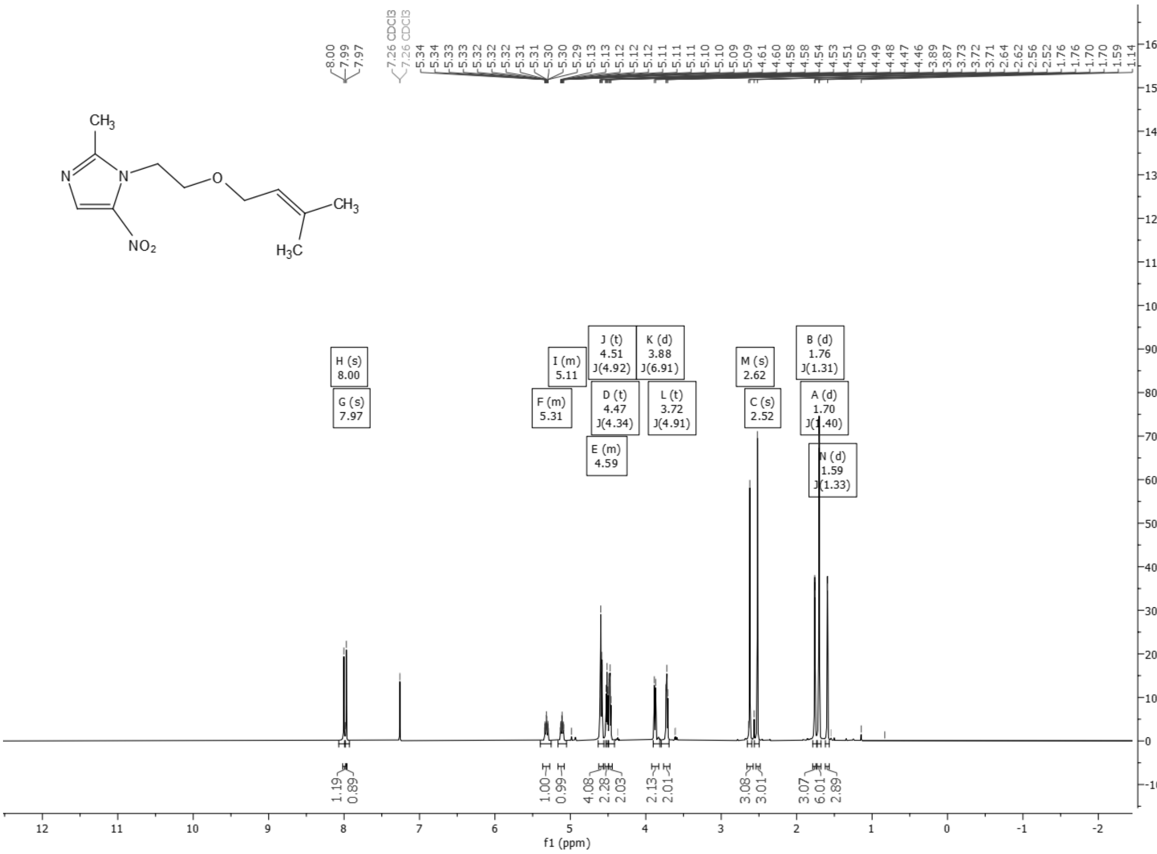

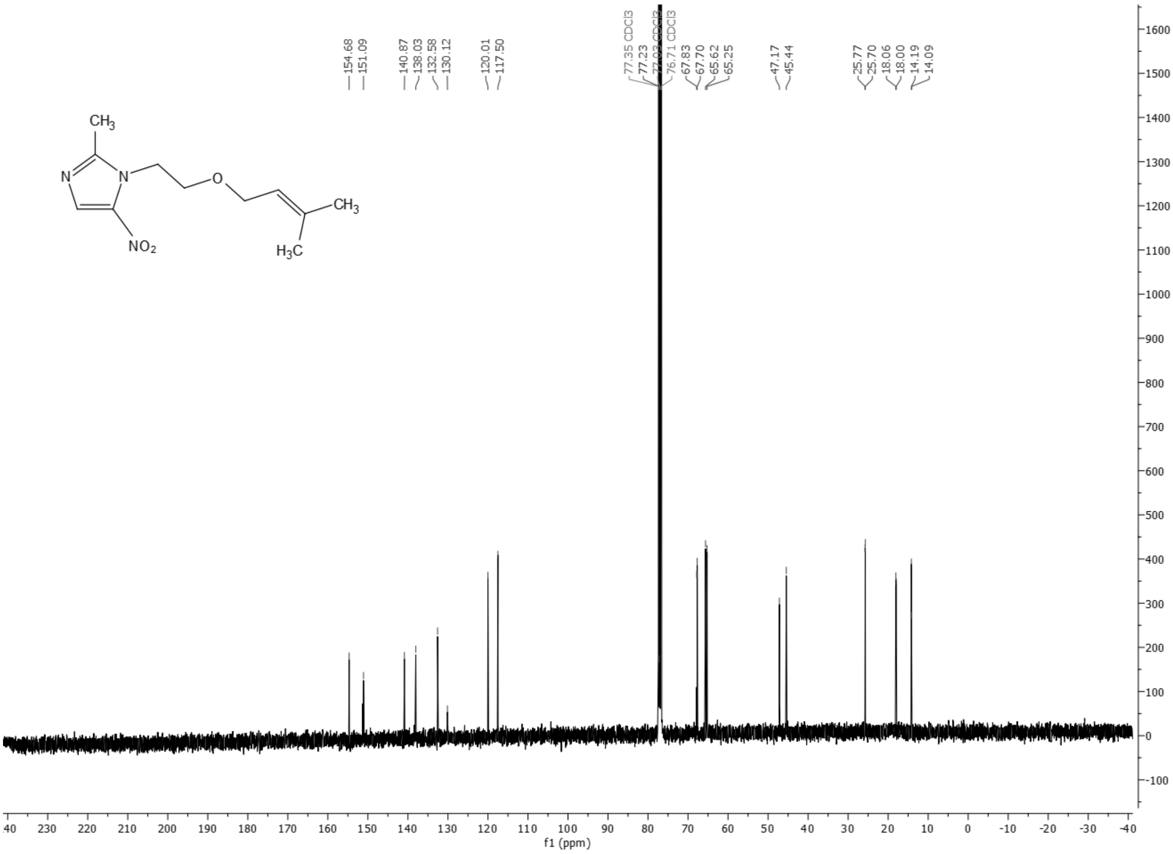

^{1}H and ^{13}C NMR spectra of **MF-09**.

MF-10

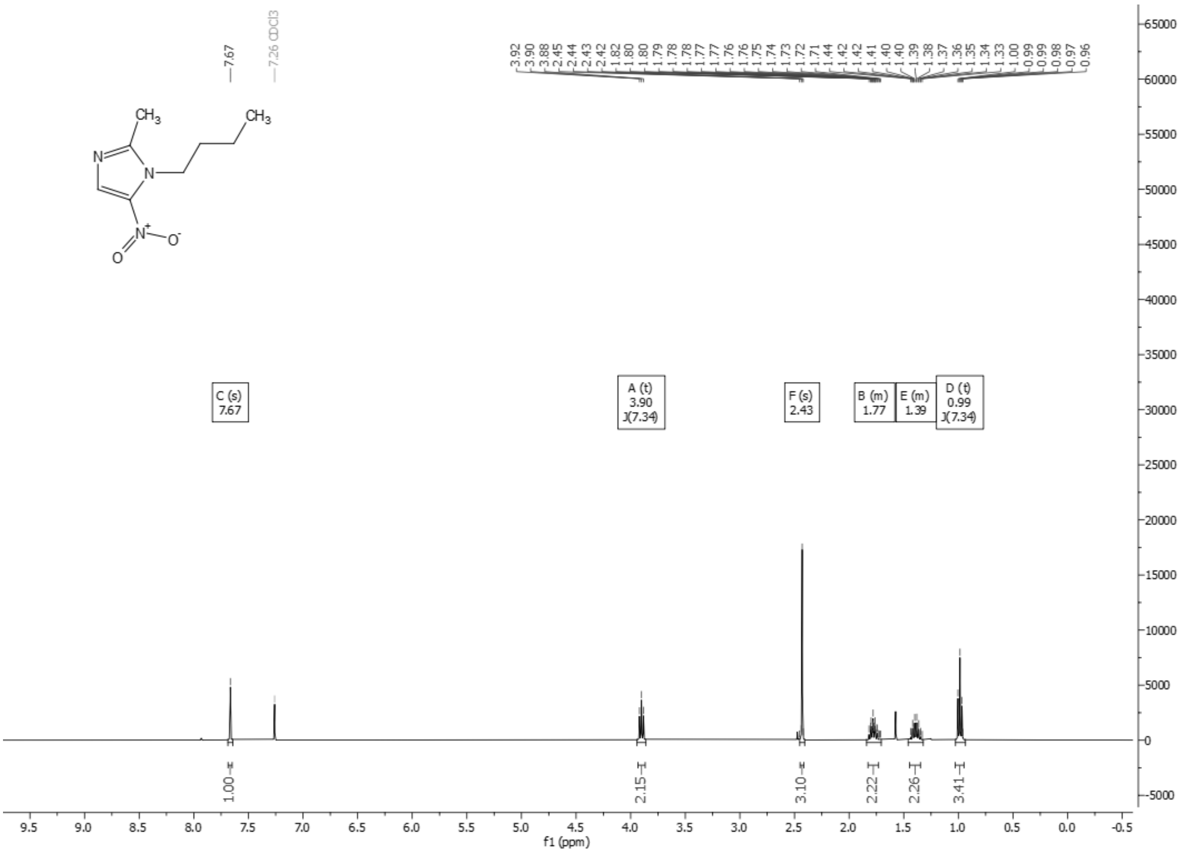

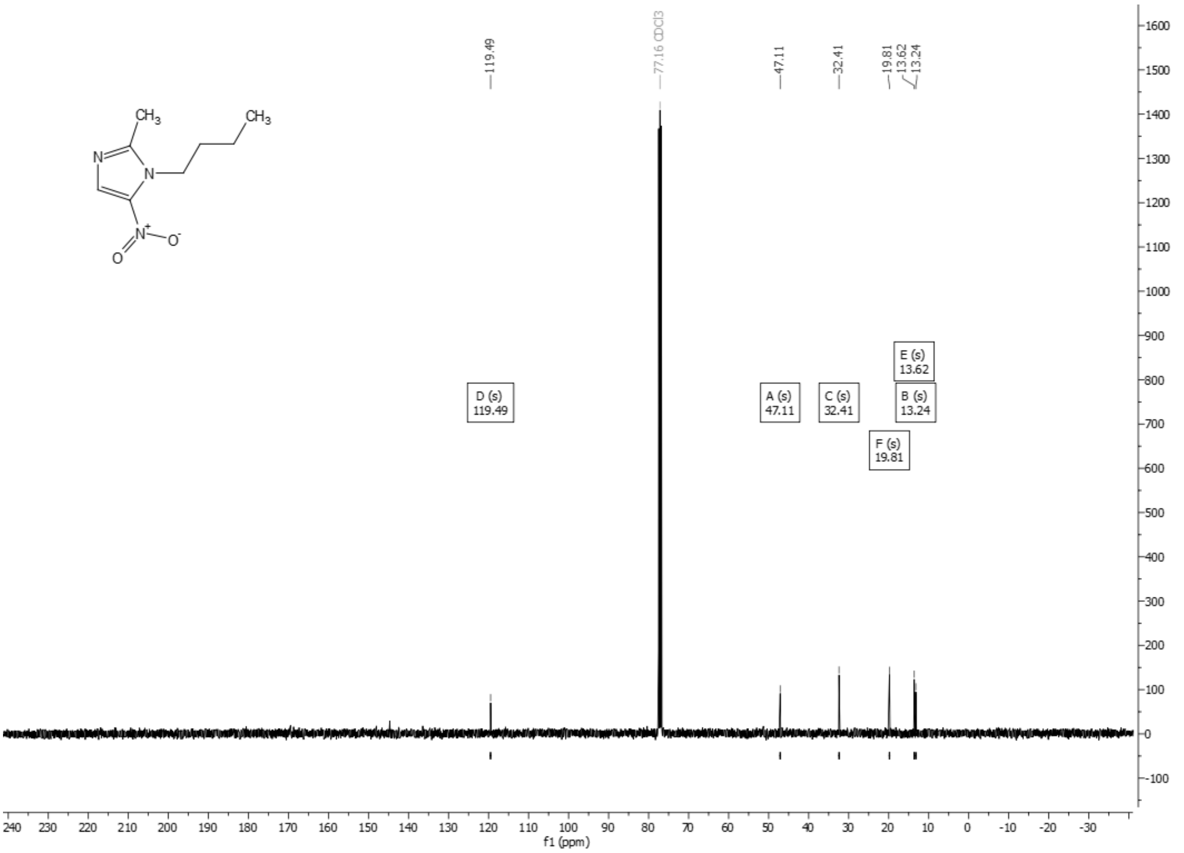

^{1}H and ^{13}C NMR spectra of **MF-10**.

MF-11

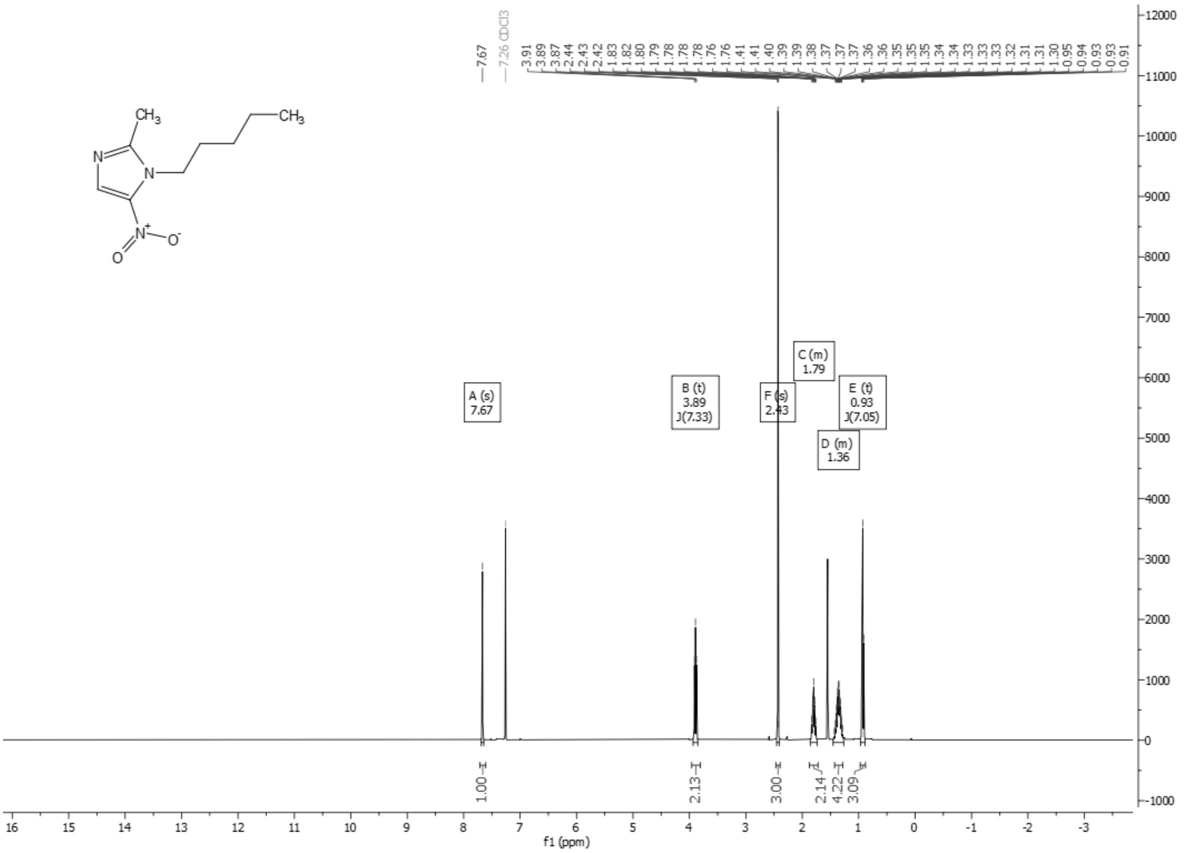

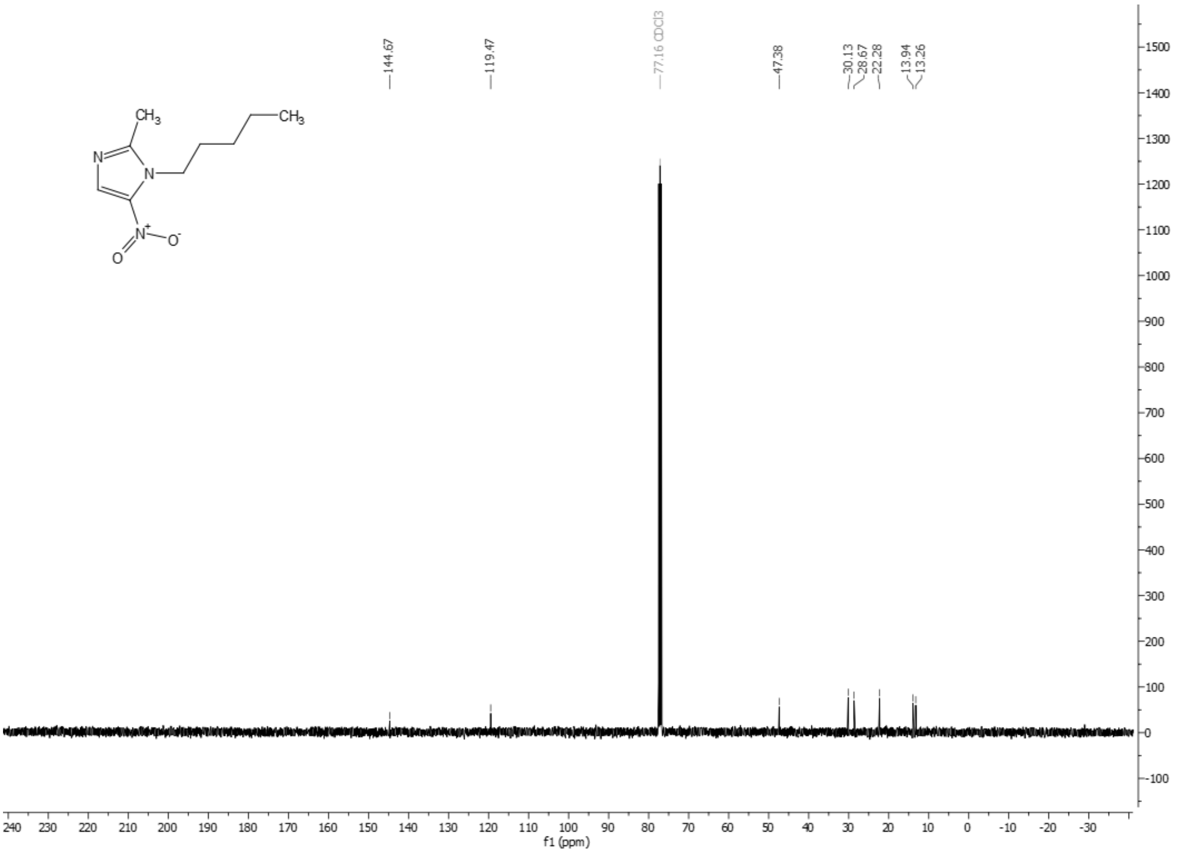

^1H and ^{13}C NMR spectra of **MF-11**.
