# Supplementary figures and images for: Metronidazole and ether derivatives target Helicobacter pylori via simultaneous stress induction and inhibition
Source: Nat Microbiol. 2026 Mar 18;11(4):1049–63. doi: 10.1038/s41564-026-02291-w (PMC13056558; doi:10.1038/s41564-026-02291-w)

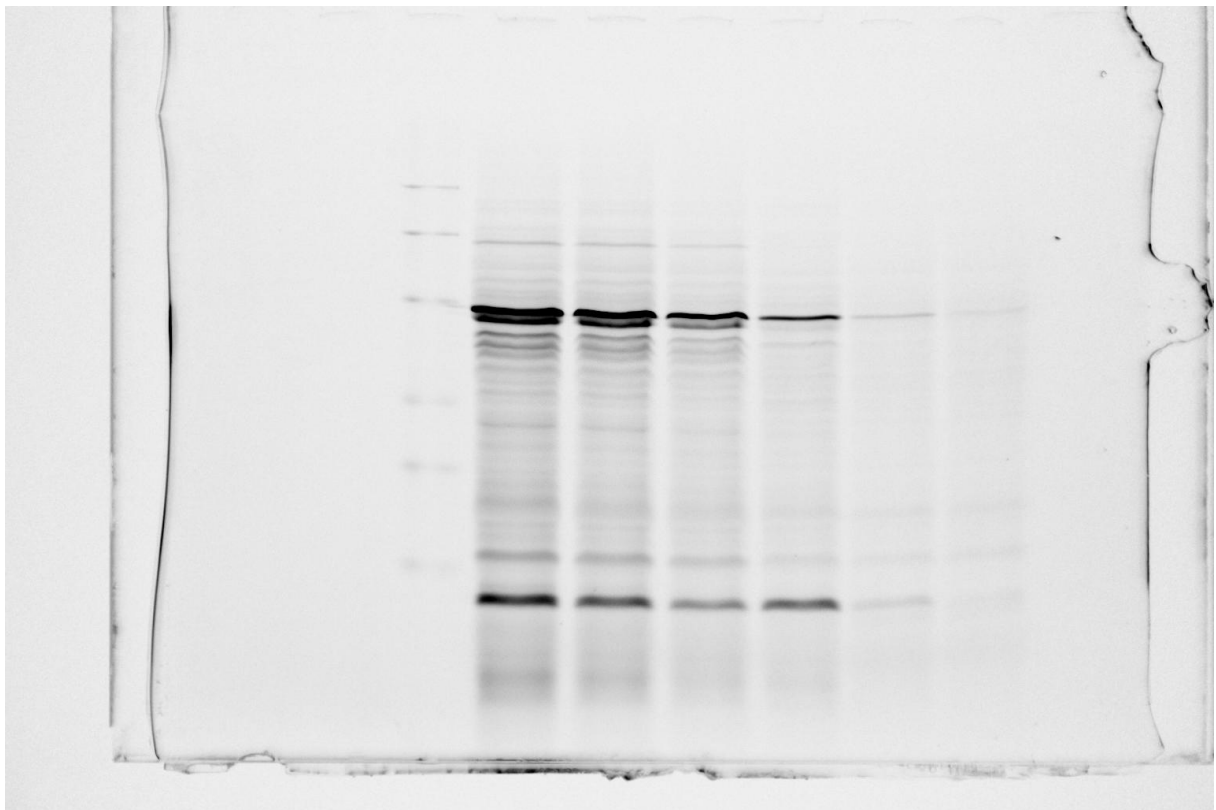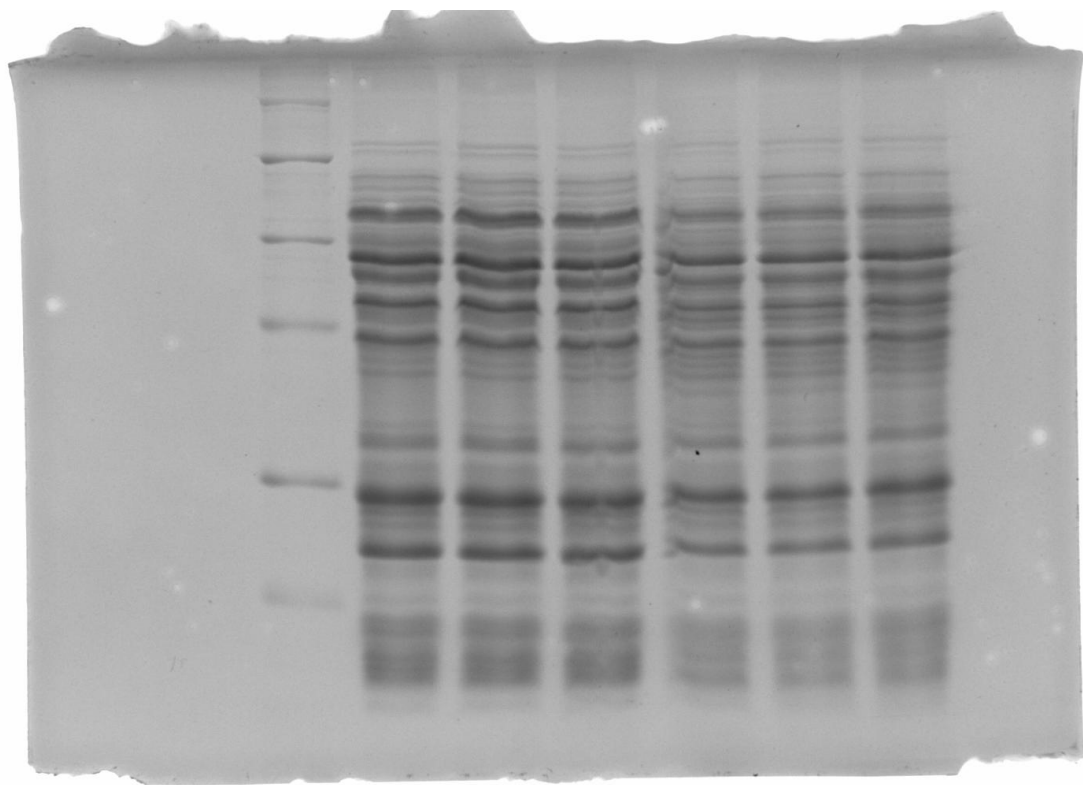

Figure 2C

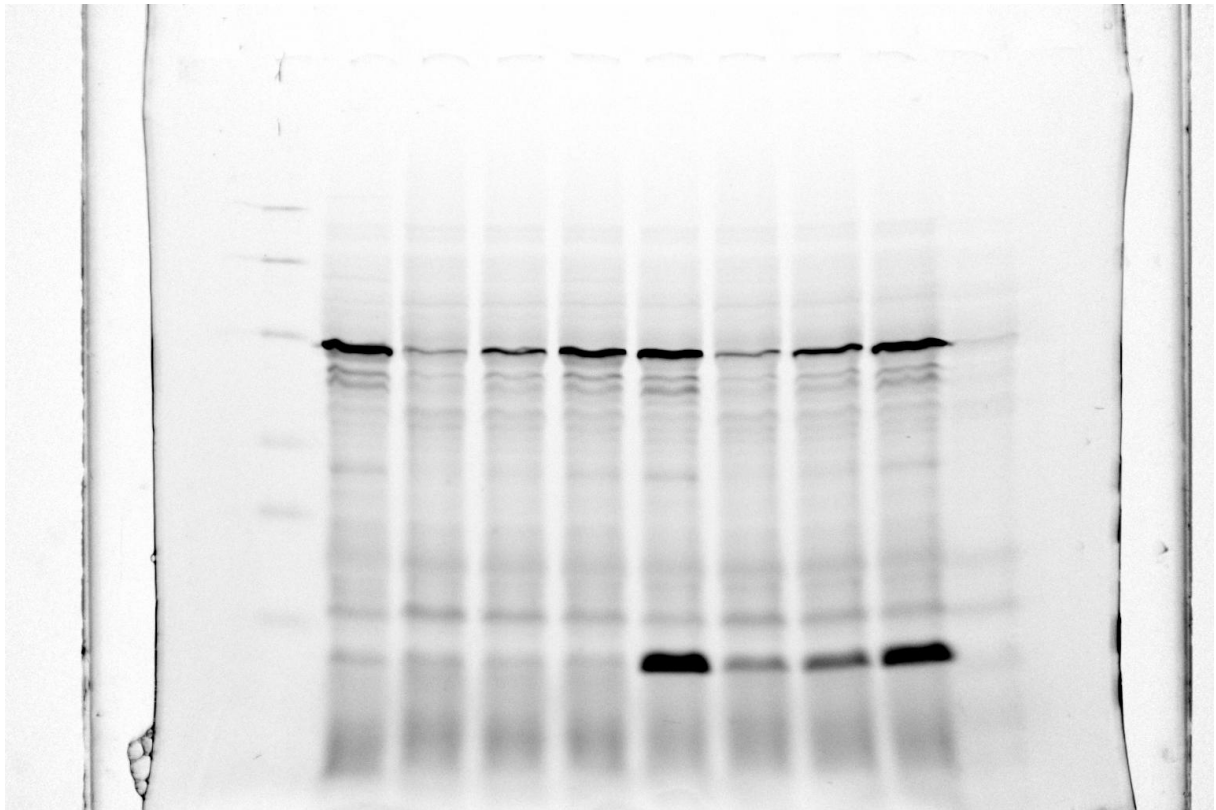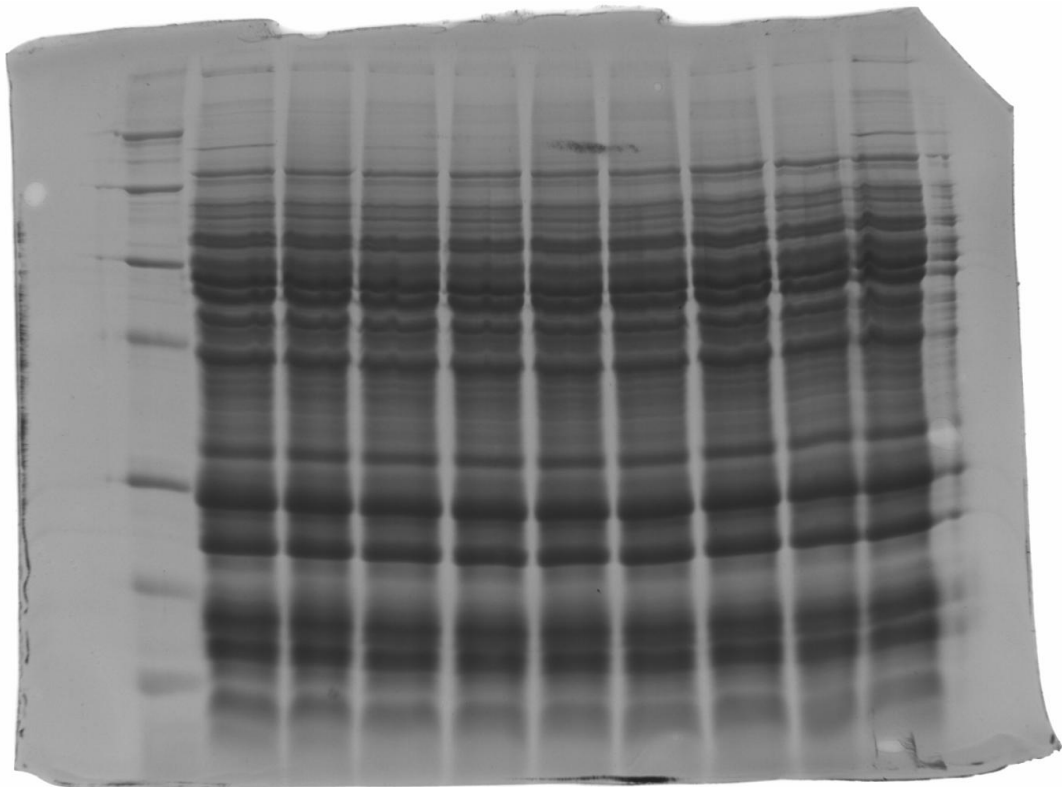

Figure 2C

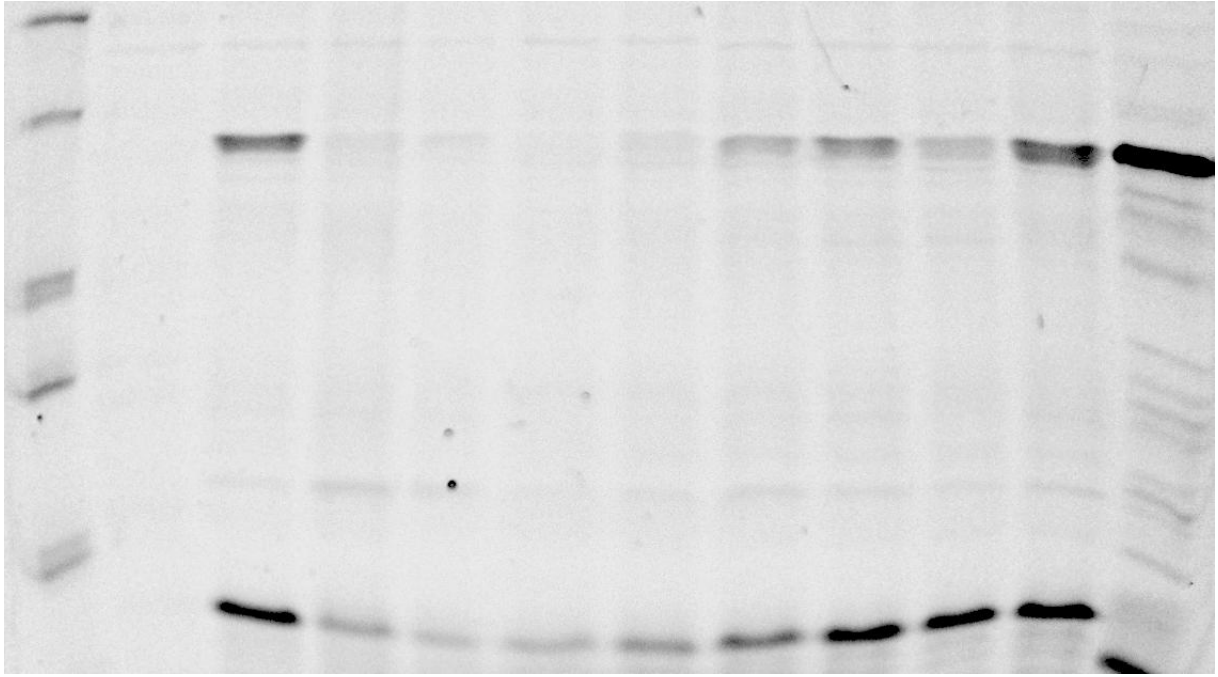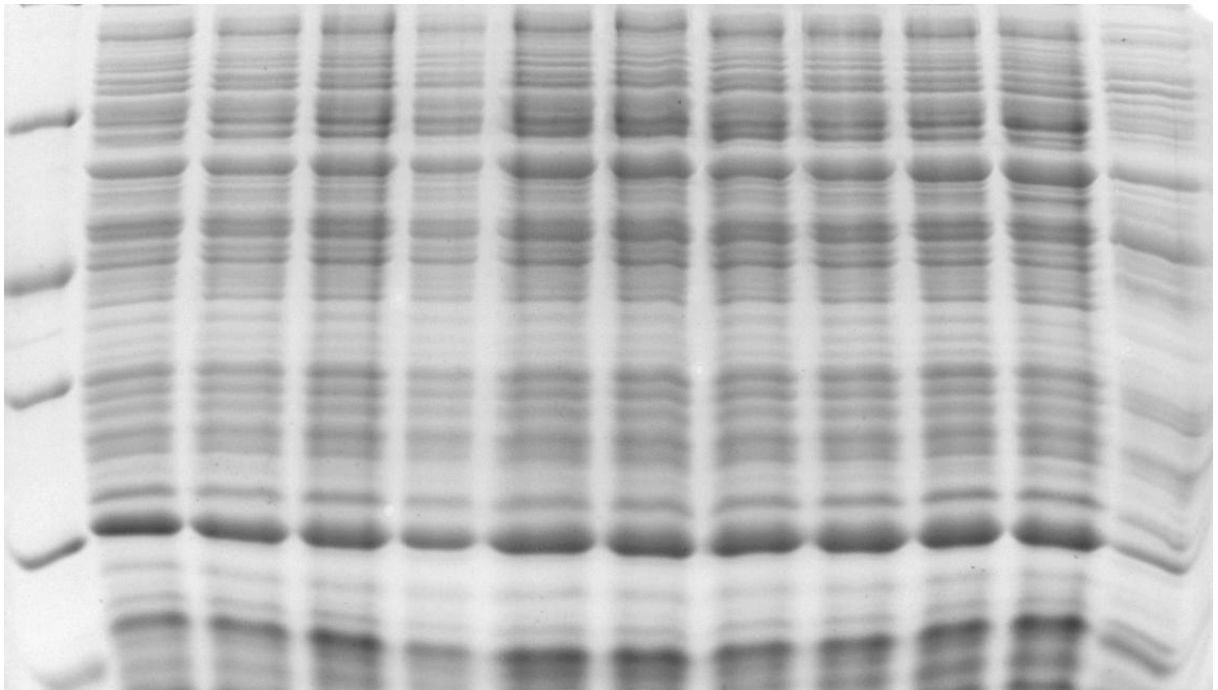

Figure 2E

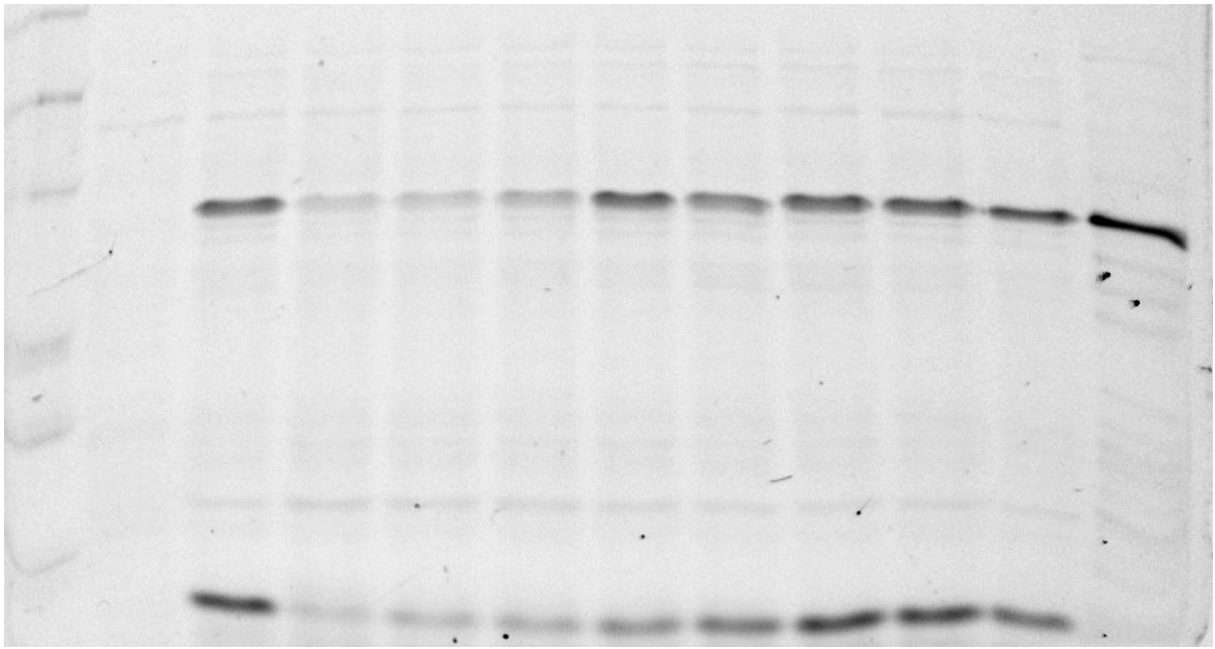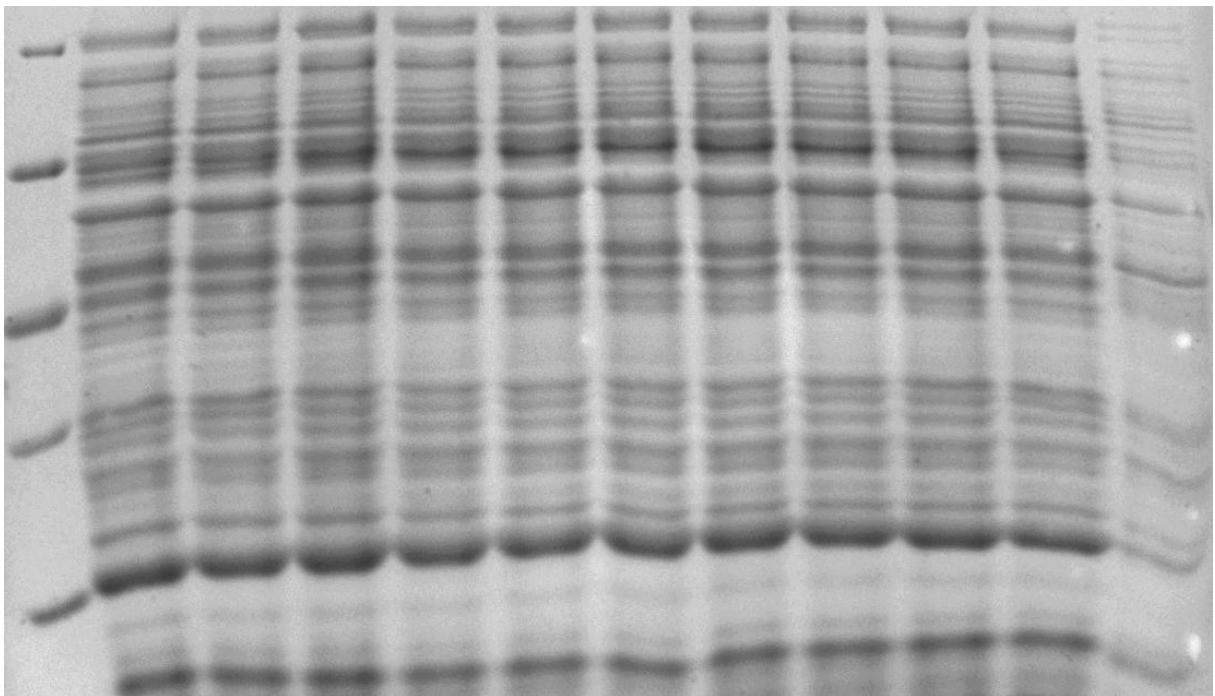

Figure 2F

Supplement: Supplementary file 13 — Unprocessed gels. [file 41564_2026_2291_MOESM13_ESM.pdf]
